# Hyperhardness and hypersoftness of atoms and their ions

**DOI:** 10.1007/s00894-024-06136-3

**Published:** 2024-09-21

**Authors:** Jarosław Zaklika, Piotr Ordon, Ludwik Komorowski

**Affiliations:** 1 Department of Physical and Quantum Chemistry, Wrocław University of Science and Technology, Wyb. Wyspiańskiego 27, 50-370 Wrocław, Poland; 2 Department of Physics and Biophysics, Wrocław University of Environmental and Life Sciences, Ul. Norwida 25, 50-373 Wrocław, Poland

**Keywords:** Atoms, Hyperhardness, Hypersoftness

## Abstract

**Context:**

The theory of reactivity based on cDFT has been supplemented with the new method of calculating the atomic and local indices. With the use of previously derived relationship of the electron density gradient to the softness kernel and to the linear response function, we deliver theoretical analysis to obtain significant reactivity indices—the electron density derivatives: local softness and local hypersoftness together with the global hyperhardness index and the derivative of the global softness with respect to the number of electrons. The local derivatives have been applied in the calculation of responses of atoms to perturbation by an external potential by the alchemical approach. The vital role of the local softness has been confirmed; the potential role of the hypersoftness has been indicated.

**Method:**

Our original theoretical scheme has been numerically illustrated with the results obtained with electron density calculations with B3LYP method implemented in Gaussian 16 package. The *aug-cc-pvqz* basis set has been routinely applied, except for the Ca atom (*cc-pvqz*). Using the *pVTZ* basis set recommended by Sadlej was necessary for the potassium atom.

**Graphical Abstract:**

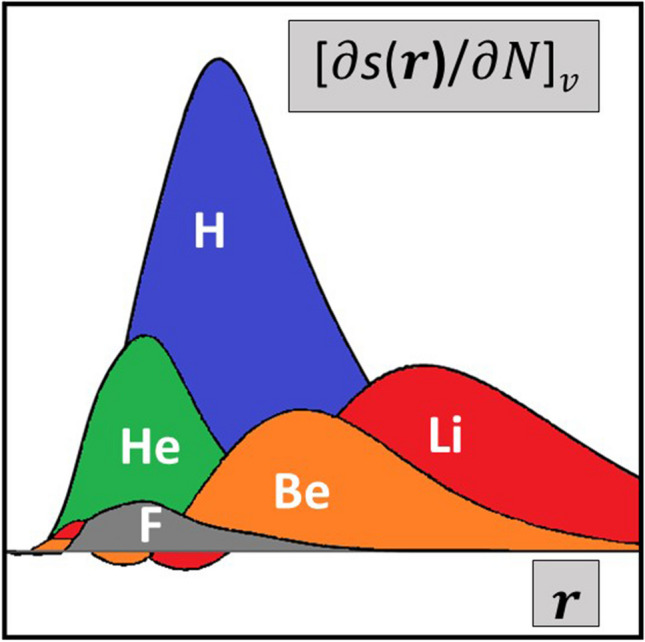

**Supplementary Information:**

The online version contains supplementary material available at 10.1007/s00894-024-06136-3.

## Introduction

The conceptual density functional theory (cDFT) has been widely recognized as the potential apparatus for theoretical exploration aiming at a priori prediction of chemical reactivity of atoms and molecules. Its birth was marked by the landmark paper by Parr et al. [1] where chemical potential derived from the Hohenberg and Kohn theorem [2] has been identified as electronegativity. Further development was clearly divided between the theory and application. In the theoretical advances, the significant step has been done with the paper by Perdew et al. [3] where Hohenberg–Kohn theory was formulated for fractional number of electrons, for systems described by a quantum mixed state. The practical applications of these theoretical achievements were remarkably initiated by the paper by Parr and Pearson [4]. While innumerable successes have been reported in both directions, coupling the two fields together into the coherent system of “molecular thermodynamics” is still in progress [5, 6].

The proposal of axiomatic approach to the chemical concepts by Ayers et al. [7] exposed once again the ever-growing disparity between the language of chemistry with its traditional concepts (atoms, bonds) and the wealth of quantum chemistry tools. The short paper with the exhausting bibliography has confirmed the longstanding interest of the group in bridging the fields of practical chemistry and quantum theory [8, 9]. Interesting practical guidelines to possible merging these two separately developed and powerful methodologies have been presented: the role of observables, the free choice of basic axioms, and the role of their mathematical formulation for the concept based upon them. Interestingly, the authors concentrate on the conceptual integrity of the possible results of that approach, rather than on the computational accuracy on the output from a theoretical effort.

An example of the intuitive, independently developed application of the approach along those lines has been presented by Ordon and Komorowski on the ground of conceptual density functional theory (cDFT) [10] The Hohenberg and Kohn theorem [2] on the electron density and the existing mathematical apparatus of the DFT have been applied to construction of the density functional (DF) connectivity matrix for any molecular system in its stationary state [11, 12]. The matrix elements are given as divergences of the atomic Hellmann–Feynman forces [13] and are numerically obtained from the energy Hessian of the system. They have strict physical interpretation as the cumulative (or effective [14–16]) force constants between any pair of localized nuclei, providing the clear and unique physical measure for the bonding status between atoms situated in a molecular array. Individual bonded atoms have been characterized accordingly: the diagonal terms of the DF connectivity matrix reflect the valence of the atom in a molecule and the variation of the elements of the DF connectivity matrix with the reaction progress has been demonstrated to provide the much appealing picture of the modification of the bonding status of atoms along IRC (reaction fragility spectra) [17–20]. The vibrational atomic modes emerging from this analysis [10] are in close relation to the calculated adiabatic internal vibrational modes AIMO by Kraka et al. [21, 22]. Review of many other attempts and approaches to the reaction force constant may be found in the recent papers [23, 24].

The results of the reaction fragility spectra have prompted us to developing the theoretical concept even further. We have derived the following gradient theorem from the basic properties of the connectivity matrix: [10, 25].

1 $$\nabla \rho \left(\mathbf{r}\right)=\int \omega \left(\mathbf{r},{\mathbf{r}}^{\mathbf{^{\prime}}}\right){\varvec{\upvarepsilon}}\left(\mathbf{r}\mathbf{^{\prime}}\right)d\mathbf{r}\mathbf{^{\prime}}=-\int s\left(\mathbf{r},{\mathbf{r}}^{\mathbf{^{\prime}}}\right){\varvec{\upvarepsilon}}\left(\mathbf{r}\mathbf{^{\prime}}\right)d\mathbf{r}\mathbf{^{\prime}}$$

where

$$\omega \left(\mathbf{r},{\mathbf{r}}^{\mathbf{^{\prime}}}\right)={\left[\frac{\delta \rho (\mathbf{r})}{\delta v\left(\mathbf{r}\mathbf{^{\prime}}\right)}\right]}_{N}$$ 
is the linear response function,
$${\varvec{\upvarepsilon}}\left(\mathbf{r}\right)=\sum_{A}{{\varvec{\upvarepsilon}}}_{A}\left(\mathbf{r}\right)$$ 
is the total external electric field, and
$$s\left(\mathbf{r},{\mathbf{r}}^{\mathbf{^{\prime}}}\right)={\left[\frac{\delta \rho (\mathbf{r})}{\delta v\left(\mathbf{r}\mathbf{^{\prime}}\right)}\right]}_{\mu }$$ 
is the softness kernel.

This exact formula represents the much-needed relation between the electron density and the electric field from the nuclei in every point of a system ${\varvec{\upvarepsilon}}\left(\mathbf{r}\right),$ very much in the spirit of the analysis by Bader [26]. The clear mathematical form of this gradient theorem and the unveiled equivalence between the use of the linear response function $\omega \left(\mathbf{r},{\mathbf{r}}^{\mathbf{^{\prime}}}\right)$ and the softness kernel $s\left(\mathbf{r},{\mathbf{r}}^{\mathbf{^{\prime}}}\right);$ the nonlocal electronic response functions [27–30]  Eq. 1 has opened a way to exploration of its further consequences. We combined it with another cDFT axiom—the nearsightedness of matter, that can be applied to $s\left(\mathbf{r},{\mathbf{r}}^{\mathbf{^{\prime}}}\right)$ but not to $\omega \left(\mathbf{r},{\mathbf{r}}^{\mathbf{^{\prime}}}\right)$ [31–33]. Vela and Gázquez [34] proposed the approximate expression for the softness kernel:

2a $$s\left(\mathbf{r},{\mathbf{r}}^{\mathbf{^{\prime}}}\right)\approx \delta \left(\mathbf{r}-{\mathbf{r}}^{\mathbf{^{\prime}}}\right)s\left({\mathbf{r}}^{\mathbf{^{\prime}}}\right)$$

Substituting to Eq. 1 and the integration of the delta function with the local softness and the electric field gives obvious result:

2b $$\int \delta \left(\mathbf{r}-{\mathbf{r}}^{\mathbf{^{\prime}}}\right)s\left({\mathbf{r}}^{\mathbf{^{\prime}}}\right){\varvec{\upvarepsilon}}\left({\mathbf{r}}^{\mathbf{^{\prime}}}\right)d{\mathbf{r}}^{\mathbf{^{\prime}}}=s\left(\mathbf{r}\right){\varvec{\upvarepsilon}}\left(\mathbf{r}\right)$$

Finally, this resulted in the recently published Ansatz of great potential utility (albeit no longer exact) allowing for calculation of the local softness: [10, 25].

2c $$\nabla \rho \left(\mathbf{r}\right)=-s\left(\mathbf{r}\right){\varvec{\upvarepsilon}}\left(\mathbf{r}\right)$$

The route to calculation of the local softness $s\left(\mathbf{r}\right)={\left[\frac{\partial \rho \left(\mathbf{r}\right)}{\partial \mu }\right]}_{v}=-{\left[\frac{\delta N}{\delta v\left(\mathbf{r}\right)}\right]}_{\mu }$ describing the response of the electron density to the electric field has been opened. The necessary test of this approach was achieved by calculation of the global softness (*S*) of atoms and ions directly by integration of the local softness *s*(**r**) resulting from Eq. 2 and calculated from the computable electron density alone. Consequently, another important local response function has also become available directly from the density function: the Fukui function *f*(**r**) = *s*(**r**)/*S* [25].

The step towards the search for higher response functions has been made by the analytical calculation of the derivatives ${[\partial f\left(\mathbf{r}\right)/\partial N]}_{v}\;\text{and}\; {[\partial s\left(\mathbf{r}\right)/\partial N]}_{v}$ for the hydrogenic orbitals [35]. This present work delivers the theoretical and computational scheme to obtain the local hypersoftness of atoms and their ions. This is possibly non-negligible reactivity index that contributes to the global changes of the state functions (Δ*N*, Δ*E*, Δ*μ*), formally replacing the nonlocal response functions, as demonstrated in our previous paper [35]. Numerical tests of this concept present a collection of results for atoms and ions, and they are aimed at estimation of the role of first and second local derivatives of their density over *N* for the physically observable effects of change Δ*N*, Δ*E*, and Δ*μ* induced in chemical reactions.

## Hyperhardness and hypersoftness

Nalewajski has developed and presented a complete theory of chemical reactivity derived from DFT and from the information theory [5, 36]. However, hyperhardness, the global energy derivative of the third order over the number of electrons $\gamma ={\left[\partial \eta /\partial N\right]}_{v}$ and other third energy derivatives, has rarely attracted attention in cDFT [37–39]. The first attempt for calculation $\gamma$ has been provided by Fuentealba and Parr [40]. Ordon and Tachibana have published original approach to obtaining $\gamma$ with the use of the nuclear reactivity indices and the Maximum Hardness Principle [41]. Later, another in-depth analysis of the local energy derivatives of higher orders has been presented in the work by Cardenas et al. [42] and the systematic and explicit approach to the whole body of cDFT energy derivatives was presented by Heidar-Zadeh et al. [43] However, no attempt to tracing a general way to their numerical values has been indicated. The widely recognized derivative proposed by Morell ${f}^{\left(2\right)}\left(\mathbf{r}\right)\equiv {\left[\frac{\partial f\left(\mathbf{r}\right)}{\partial N}\right]}_{v}={\left[\frac{{\partial }^{2}\rho \left(\mathbf{r}\right)}{\partial {N}^{2}}\right]}_{v}$ (the dual descriptor) plays a significant role within the theory of nuclear reactivity indices [44–46]. It also represents the third energy derivative and has been calculated by the finite difference method applied to the lower order derivatives [47–49]. ${f}^{\left(2\right)}\left(\mathbf{r}\right)$ has been tested in many chemical applications ever since [50–52]. Another work by Cardenas et al. provided formal arguments for application of the dual descriptor as the useful tool for predicting the regioselectivity and nucleophilic attack simultaneously [53]. However, better predictive power of the softness/hypersoftness over FF/dual index has recently been advocated by Martinez-Araya [54]: “With the use of *s*(**r**) and *s*^(2)^(**r**) we have sufficient certainty that we are carrying out a more appropriate analysis since these two local reactivity descriptors are not affected by differences in size of the systems (…)”.

The local hypersoftness ${s}^{\left(2\right)}\left(\mathbf{r}\right)\equiv {\left[\frac{\partial s\left(\mathbf{r}\right)}{\partial \mu }\right]}_{v}={\left[\frac{{\partial }^{2}\rho \left(\mathbf{r}\right)}{\partial {\mu }^{2}}\right]}_{v}$ defined in the grand canonical ensemble has not yet been calculated for atoms, either free or bonded; analytical approach to this index for the hydrogenic orbitals has been presented in our previous paper [35].

## Theoretical approach

Equation 2c allows for an alternative approach to the third energy derivatives. The basic equation for direct calculation of the local softness reads (Eq. 3): [25, 35]

3 $$s\left(\mathbf{r}\right)=-\frac{\nabla\uprho \left(\mathbf{r}\right)\cdot {\varvec{\upvarepsilon}}\left(\mathbf{r}\right)}{{\left|{\varvec{\upvarepsilon}}\left(\mathbf{r}\right)\right|}^{2}}$$

Hence:

4 $${\left[\frac{ds(\mathbf{r})}{dN}\right]}_{v}=-\frac{\nabla f\left(\mathbf{r}\right)\cdot {\varvec{\upvarepsilon}}\left(\mathbf{r}\right)}{{\left|{\varvec{\upvarepsilon}}\left(\mathbf{r}\right)\right|}^{2}}$$

This is transformed into working equation: [35]

5 $${\left[\frac{ds(\mathbf{r})}{dN}\right]}_{v}=-\frac{1}{S}\frac{{\nabla }^{2}\uprho \left(\mathbf{r}\right)}{{\left|{\varvec{\upvarepsilon}}\left(\mathbf{r}\right)\right|}^{2}}$$

This result is computable once the global softness $S$ has been determined at the preceding step by integration of the local softness (Eq. 3); the global derivative ${\left[\partial S/\partial N\right]}_{v}$ is obtained from Eq. 5 accordingly. Calculation of this global derivative is sufficient for finding the global hyperhardness [41]:

6a $$\gamma ={\left[\frac{\partial \eta }{\partial N}\right]}_{v}=-\frac{1}{{S}^{2}}{\left[\frac{\partial S}{\partial N}\right]}_{v}$$

Hyperhardness is defined within the canonical ensemble (closed-system representation [42, 47]) with the energy function as canonical potential, dependent on the external potential and the number of electrons $E[v\left(\text{r}\right), N]$. On the other hand, the definition of the hypersoftness reads:

6b $${{S}^{(2)}\equiv \left[\frac{\partial S}{\partial \mu }\right]}_{v}= \, {-\left[\frac{{\partial }^{3}\Omega }{\partial {\mu }^{3}}\right]}_{v}$$

As the global softness itself, *S*^(2)^ is defined in the grand canonical ensemble (open-system representation [42, 47]), where the chemical potential *μ* is an independent variable for the grand potential: $\Omega [v(\mathbf{r}),\mu ]=E-\mu N$ [42, 47]*.* Since the variables *N* and *μ* are related, the hypersoftness ${S}^{(2)}$ may be expressed in both ensembles: [35]

7 $${S}^{(2)} \, {=\left[\frac{\partial S}{\partial \mu }\right]}_{v}\text{=}{\left[\frac{\partial S}{\partial N}\right]}_{v}S$$

The relationship of hypersoftness with the hyperhardness follows by combining Eq. 7 with Eq. 6a.

8 $${S}^{(2)}=-\gamma {S}^{3}$$

The local hypersoftness is defined and expressed accordingly:

9 $${s}^{(2)}\left(\mathbf{r}\right)\equiv {\left[\frac{\partial s\left(\mathbf{r}\right)}{\partial \mu }\right]}_{v}={\left[\frac{{\partial }^{2}\rho \left(\mathbf{r}\right)}{\partial {\mu }^{2}}\right]}_{v}={\left[\frac{\partial s\left(\mathbf{r}\right)}{\partial N}\right]}_{v}S$$

It has been demonstrated in the preceding work that the dual descriptor can be expressed in a similar form: [35]

10 $${f}^{(2)}\left(\mathbf{r}\right)=\frac{1}{S}{\left[\frac{\partial s\left(\mathbf{r}\right)}{\partial N}\right]}_{v}-\frac{f\left(\mathbf{r}\right)}{S}{\left[\frac{\partial S}{\partial N}\right]}_{v}$$

As evidenced by Eqs. 7–9, the computational access to the single derivative ${\left[\frac{\partial s\left(\mathbf{r}\right)}{\partial N}\right]}_{v}$ is sufficient for reproducing both the global hyperhardness *γ* and hypersoftness ${S}^{(2)}$; hence for both ensembles, we obtain the operating formulas for the whole collection of reactivity indices being the third-order energy derivatives [35].

## cDFT analysis of the state functions

In the previous papers [25, 35], we have obtained analytical results for global parameters, vital for the description of the chemical reaction: $\Delta N$, $\Delta E$, and $\Delta \mu$. They were calculated within the alchemical approach [55–57], up to the second order for both open and closed systems, using the operating approximations to the local softness (Eq. 3) and its derivative over *N* (Eq. 5) [35]. This has motivated the computational results of this present paper. With the use of $s\left(\mathbf{r}\right)$ and ${[\partial s\left(\mathbf{r}\right)/\partial N]}_{v}$, we have derived new formulas for hyperhardness and hypersoftness that come from Eq. 6a and Eq. 6b and we have tested them for atoms and ions.

The local approximation [25] and the Berkowitz-Parr formula [27] allowed for replacing the kernels by the local derivatives in the Taylor expansion into the derivatives over $v\left(\mathbf{r}\right)$. Canonical ensemble expansion has been presented for energy $\Delta {E}_{N}$ and chemical potential $\Delta {\mu }_{N}$ at constant *N* (closed system), while for $\Delta {N}_{\mu }$, the grand canonical ensemble/open system with the constraint of constant *μ* was appropriate. The results are: [35]

11 $$\Delta {E}_{N}=\int \rho (\mathbf{r})\Delta v(\mathbf{r})d\mathbf{r}-\frac{1}{2}\left\{\int s(\mathbf{r}){\left[\Delta v(\mathbf{r})\right]}^{2}d\mathbf{r}-\frac{1}{S}{\left[\int s(\mathbf{r})\Delta v(\mathbf{r})d\mathbf{r}\right]}^{2}\right\}$$

12 $$\begin{aligned}\Delta {\mu }_{N}&=\frac{1}{S}\int s(\mathbf{r})\Delta v(\mathbf{r})d\mathbf{r}+\\&+\frac{1}{2}\left\{\begin{array}{l}-\int {\left[\frac{\partial s(\mathbf{r})}{\partial N}\right]}_{\mu }{\left[\Delta v(\mathbf{r})\right]}^{2}d\mathbf{r}+\\ +\gamma {\left[\int s(\mathbf{r})\Delta v(\mathbf{r})d\mathbf{r}\right]}^{2}+\\ +\frac{2}{S}\int {\left[\frac{\partial s(\mathbf{r})}{\partial N}\right]}_{\mu }\Delta v(\mathbf{r})d\mathbf{r}\int s(\mathbf{r}^{\prime})\Delta v(\mathbf{r}^{\prime})d\mathbf{r}^{\prime}\end{array}\right\}\end{aligned}$$

13 $$\Delta {N}_{\mu }=-\int s(\mathbf{r})\Delta v(\mathbf{r})d\mathbf{r}-\frac{S}{2}\int {\left[\frac{\partial s\left(\mathbf{r}\right)}{\partial N}\right]}_{v}{\left[\Delta v(\mathbf{r})\right]}^{2}d\mathbf{r}$$

The energy formula (Eq. 11) does not involve the third-order density derivatives in the expansion to the second order, since its first derivative over *v*(**r**) is the density itself.

Within the alchemical approach to atoms and atomic ions, the variation of the potential $\Delta v\left(\mathbf{r}\right)$ comes uniquely from the variation of the atomic number $\Delta Z$ [35]. The integrals necessary in Eqs. 11–13 are transformed and the brief notation for the five new necessary integrals has been introduced (Eqs. 14–19):

14 $$\int \rho \left(\mathbf{r}\right)\Delta v\left(\mathbf{r}\right)d\mathbf{r}=-\Delta Z\int \frac{\rho \left(r\right)}{r}dr=-\Delta Z\ {I}_{\uprho }$$

15 $$\int s(\mathbf{r})\Delta v(\mathbf{r})d\mathbf{r}=-\Delta Z\int \frac{s\left(r\right)}{r}dr=-\Delta Z\ {I}_{s}$$

16 $$\int s(\mathbf{r}){\left[\Delta v(\mathbf{r})\right]}^{2}d\mathbf{r}=-\Delta {Z}^{2}\int \frac{s\left(r\right)}{{r}^{2}}dr=-\Delta {Z}^{2}\ {I}_{\text{s}2}$$

17 $$\int {\left[\frac{\partial s\left(\mathbf{r}\right)}{\partial N}\right]}_{\mu }\Delta v\left(\mathbf{r}\right)d\mathbf{r}=-\Delta Z\int {\left[\frac{\partial s\left(r\right)}{\partial N}\right]}_{\mu }\frac{1}{r}dr=-\Delta Z\ {I}_{N}$$

18 $$\int {\left[\frac{\partial s(\mathbf{r})}{\partial N}\right]}_{\mu }{\left[\Delta v(\mathbf{r})\right]}^{2}d{\varvec{r}}=-\Delta {Z}^{2}\int {\left[\frac{\partial s(r)}{\partial N}\right]}_{\mu }\frac{1}{{r}^{2}}dr=-\Delta {Z}^{2} {I}_{N2}$$

Three basic equations (Eqs. 11–13) may now be transformed to the explicit functions of the atomic number Δ*Z*.

19 $$\Delta {E}_{N}=-\Delta Z {I}_{\uprho }+\frac{1}{2}\left[{I}_{\text{s}2}+\frac{1}{S}{\left({I}_{\text{s}}\right)}^{2}\right]\Delta {Z}^{2}={A}_{E}\Delta Z+{B}_{E}\Delta {Z}^{2}$$

20 $$\Delta {\mu }_{N}=-\Delta Z \frac{{I}_{\text{s}}}{S}+\frac{1}{2}\left[{I}_{N2}-\gamma {\left({I}_{\text{s}}\right)}^{2}+\frac{2}{S}{I}_{N}{I}_{\text{s}}\right]\Delta {Z}^{2}={A}_{\mu }\Delta Z+{B}_{\mu }\Delta {Z}^{2}$$

21 $$\Delta {N}_{\mu }=\Delta Z {I}_{\text{s}}+\frac{S}{2}{I}_{N2}\Delta {Z}^{2}\hspace{0.17em}=\hspace{0.17em}{A}_{N}\Delta Z+{B}_{N}\Delta {Z}^{2}$$

The significance of $s\left(\mathbf{r}\right)$ and ${[\partial s\left(\mathbf{r}\right)/\partial N]}_{v}$ is clearly exposed in Eqs. 11–13: they both are sufficient to obtain all necessary integrals (Eqs. 14–18). The abbreviated symbols for various integrals within the alchemical approach have been introduced for the sake of clarity (Eqs. 14–18). The results for $\Delta {E}_{N}$, $\Delta {\mu }_{N}$, and $\Delta {N}_{\mu }$ are all parabolic functions of $\Delta Z$ with the local hypersoftness function contributing to the variation of each state function in the second order only. The significance of this term for the quantitative results of $\Delta {E}_{N}$, $\Delta {\mu }_{N}$, and $\Delta {N}_{\mu }$ has been analyzed in detail for the group of atoms representing the highest values of global softness *S* and global hypersoftness and ${[\partial S/\partial N]}_{v}$.

## Numerical results

Computational results of this present work have been obtained for the 36 atoms (1–4 row of the periodic table) and corresponding cations and anions [25].

### Computational methods

Numerical analysis was executed with the Gaussian 16 code [58]. The B3LYP method has been chosen, following its former successful tests in the electron density calculations in atoms [59–61]. The *aug-cc-pvqz* basis set has been routinely applied, except for the Ca atom (*cc-pvqz*). Using the *pVTZ* basis set recommended by Sadlej was necessary for the potassium atom [62]. The ground states of atoms have been identified by the specification of atomic electronic terms [25, 63].

The numerical DFT method has been used to calculate the electron density and electron density gradient to overview the local softness of atoms and ions. The effect of degeneracy of frontier orbitals has been avoided by using the integral electron density for every atom and ion. As it has been proved by Kohn, the ground state density is unique even for systems, where frontier orbitals are degenerate [64, 65]. The simple practical method has been applied to circumvent another well-known difficulty in reproducing spherical symmetry of the electron density in atoms. Two steps have been involved in this procedure. Integration of the electron density has been routinely made by the spherical algorithm for a density variable in one direction only. The result of integration to proper number of electrons served as evidence of a spherical symmetry of the density. For atoms whose basic electronic terms were other that *S* type, the numerical averaging procedure over principal coordinate axis and all diagonal directions has been applied to the raw computational results. The subsequent integration provided proof for the sufficient quality (symmetry) of the averaged density for the purpose of this study. The density gradient has only been calculated in one direction from the spatially averaged numerical density data. Equation 3 and Eq. 5 serve as basic computational algorithms.

The integration of the electron density and its derivatives has been obtained with the grid of the density function. The accuracy of the grid was controlled by the integration of the resulting radial distribution of the density to the proper number of electrons. The grid for atoms/ions in the 1st and 2nd periods was 0.05*a*_o_ with the integration radius 15 *a*_o_. For the 3rd and 4th periods, the grid was 0.02 *a*_o_ within the radius 20 *a*_o_.

In the traditional cDFT formalism, the global derivatives of energy over *N* have been expressed in units of energy, typically in eV. This comes from the basic formulation of the electron density, hence also the number of electrons as dimensionless and this method makes the formalism clear. However, it has been noted by March that it leads to non-physical sign of the energy ( +) while the binding energy of any system of electrons and nuclei is negative. The correction, by introducing the charge factor with the electron density: $-e\rho \left(\mathbf{r}\right)$, had been proposed by March [66, 67], following the earlier analysis by Feynman [13]. The units of electric field are clearly V/m and the local approximation leading to working equation (Eq. 2) does not affect the physical meaning of the local softness *s*(**r**). By integrating *s*(**r**) to global softness *S*, the physical unit for global softness is disclosed: *S* [e/V], equivalent to the units of capacity [*C*/*V*] = [*F*] = [*m*]. The unit of the same origin had once been proposed within the chemical approximation to the basic cDFT concepts [68–72]. Neglecting the corrections indicated by Feynman and March would lead to global softness in [V^−1^], hardly physical, in terms of the meaning of this quantity [73].

The above conclusion calls for reconciliation between the physical rigor and the widely accepted practice in cDFT. It does not seem reasonable to abandon the custom of expressing the chemical potential, global hardness (and further global derivatives in the canonical ensemble) in the specific units of energy (eV); their conversion to a.u. is evident. Therefore, since the units for softness have not yet been consolidated, it seems practical to propose keeping *S* and its derivatives in atomic units; the inconsistence between units of hardness and softness becomes immaterial in a.u.: $S \left[\text{au}\right]={\left\{\frac{\eta \left[e\text{V}\right]}{27.211 [e\text{V}/\text{au}]}\right\}}^{-1}$. This method has been applied in the presentation of the results of this work.

### Local hypersoftness calculated for atoms and ions

**Fig. 1:**
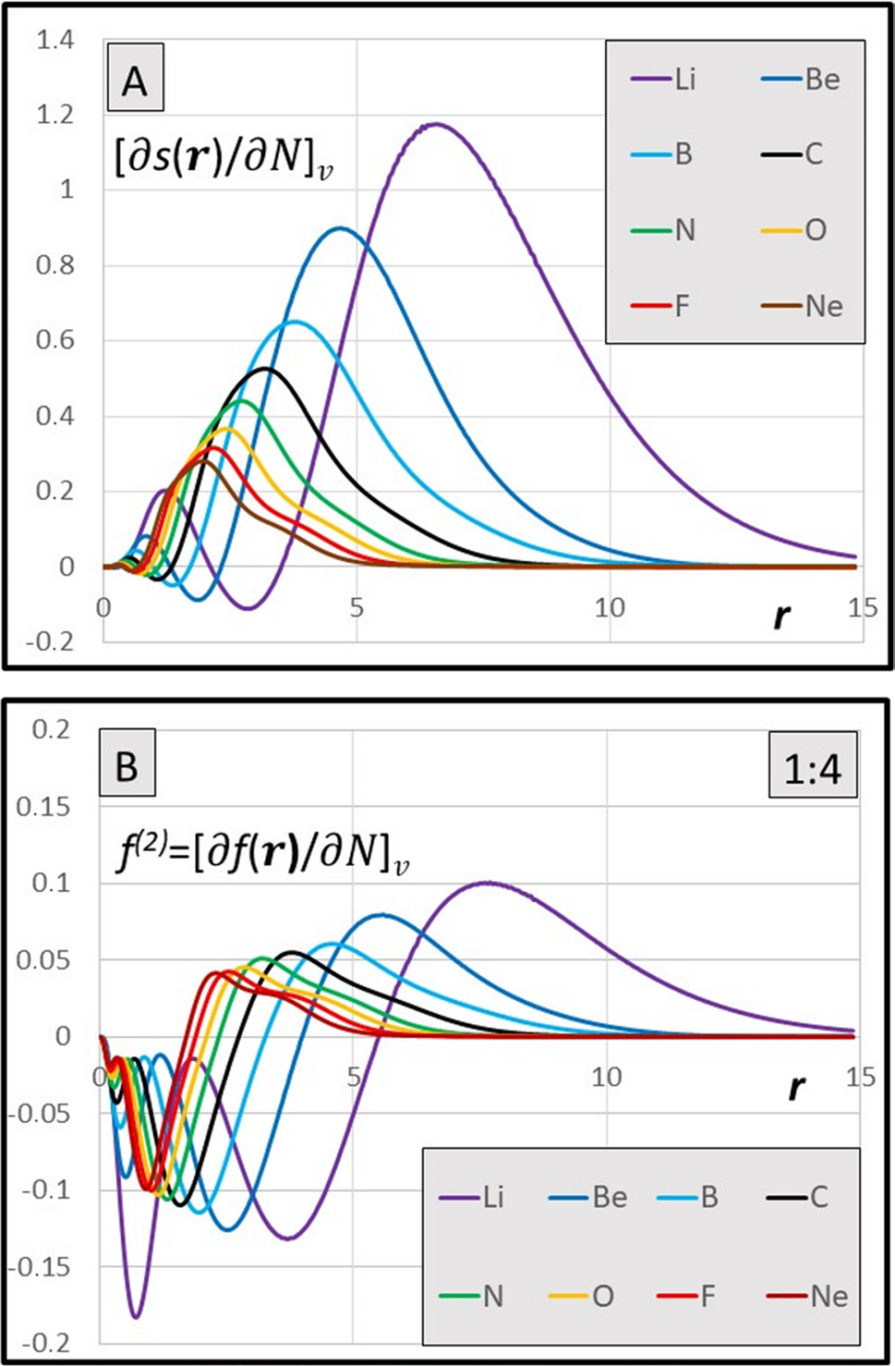
**A** Radial distribution of the derivative ${\left[\frac{ds\left(r\right)}{dN}\right]}_{v}\text{ for atoms}\; \text{in a}.\text{u}.$ (Eq. 5). **B** Radial distribution of the dual descriptor ${{f}^{(2)}\left(r\right)=[df\left(r\right)/ dN]}_{v}$ $\text{for atoms}\; \text{in}$ a.u. (Eq. 10). Note the expansion of the ordinate axis by 1:4 ratio

**Fig. 2:**
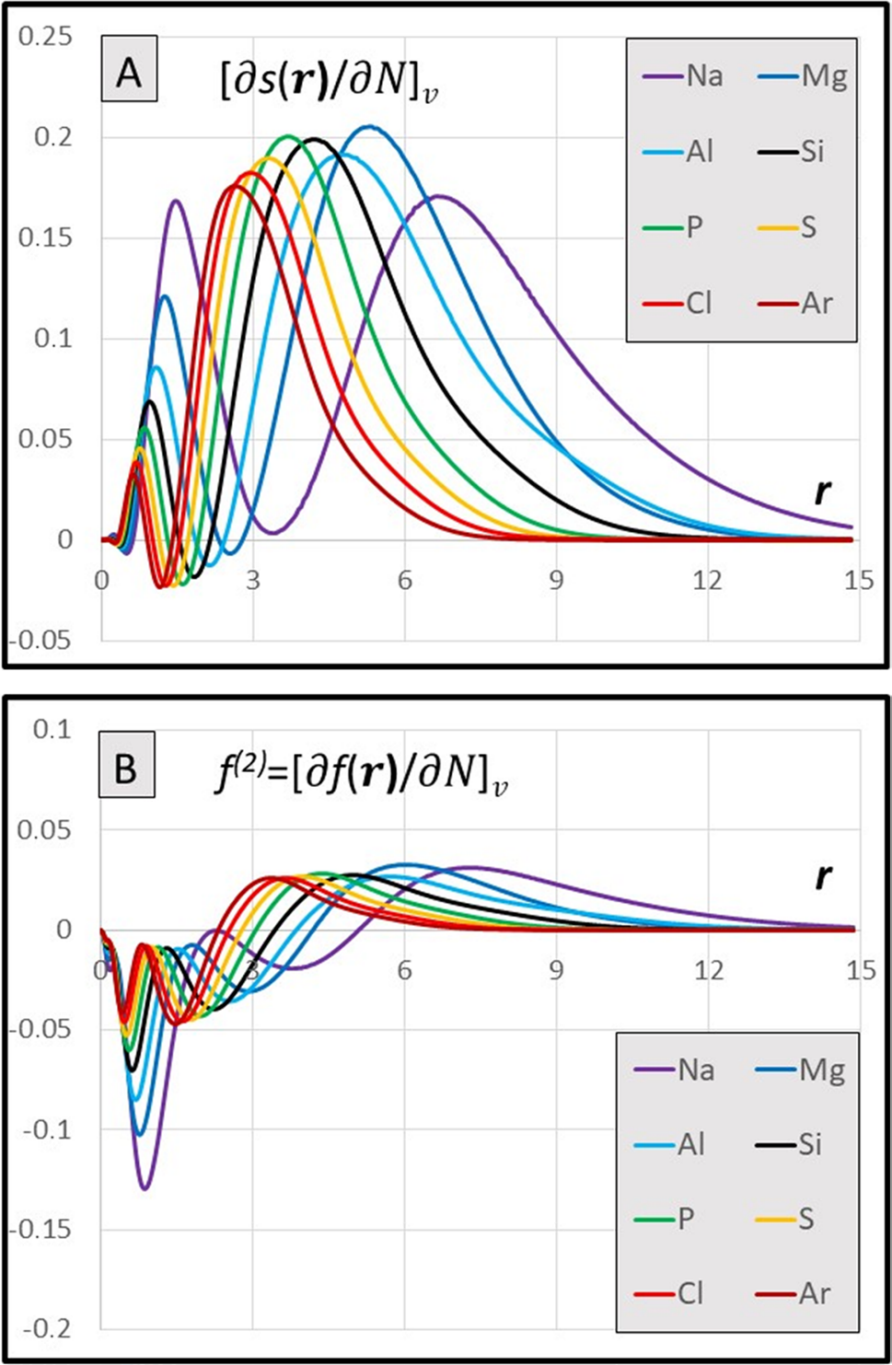
**A** Radial distribution of the derivatives ${[\partial s\left(r\right)/\partial N]}_{v}$ $\text{for atoms in a}.\text{u}.$ (Eq. 5). **B** Radial distribution of the dual descriptor ${{f}^{(2)}\left(r\right)=[\partial f\left(r\right)/\partial N]}_{v}\; \text{for atoms in a}.\text{u}$ ( Eq. 10)

The basic derivatives are presented in Figs. 1 and 2: ${\left[\partial s\left(r\right)/\partial N\right]}_{v}={s}^{\left(2\right)}\left(r\right)/S$ and the dual descriptor ${f}^{(2)}\left(r\right)={[df(r)/dN]}_{v}$ for atoms in rows 2 and 3 of the periodic table, respectively. The derivatives are interconnected by Eq. 10.

The diagrams of the ${[\partial s\left(r\right)/\partial N]}_{v}$ functions for 36 elements are presented in the Supporting Material, together with the diagrams of the dual descriptors of atoms; results for cations and anions have also been included. Except the rows 1 and 2, the derivatives ${[\partial s\left(r\right)/\partial N]}_{v}$ tend to be flat functions reaching to considerably greater distance than the local softness functions [25]. They all span positive values, except for narrow area near the nucleus, where shallow minima are typically observed (Fig. 1A and Fig. 2A). This represents the meaningful feature as compared to the dual descriptor, the analogous function that is integrated to zero, hence showing the minima and maxima of equal importance (Fig. 1B and Fig. 2B). The global values [∂*S*/∂*N*]_*v*_ resulting from integration are all positive numbers, typically reaching no more than 30% of the global softness (also positive), except the very light elements (rows 1 and 2), cf. Figure 5A and Fig. 6A and 6.

### Global hyperhardness γ and hardness η

**Fig. 3:**
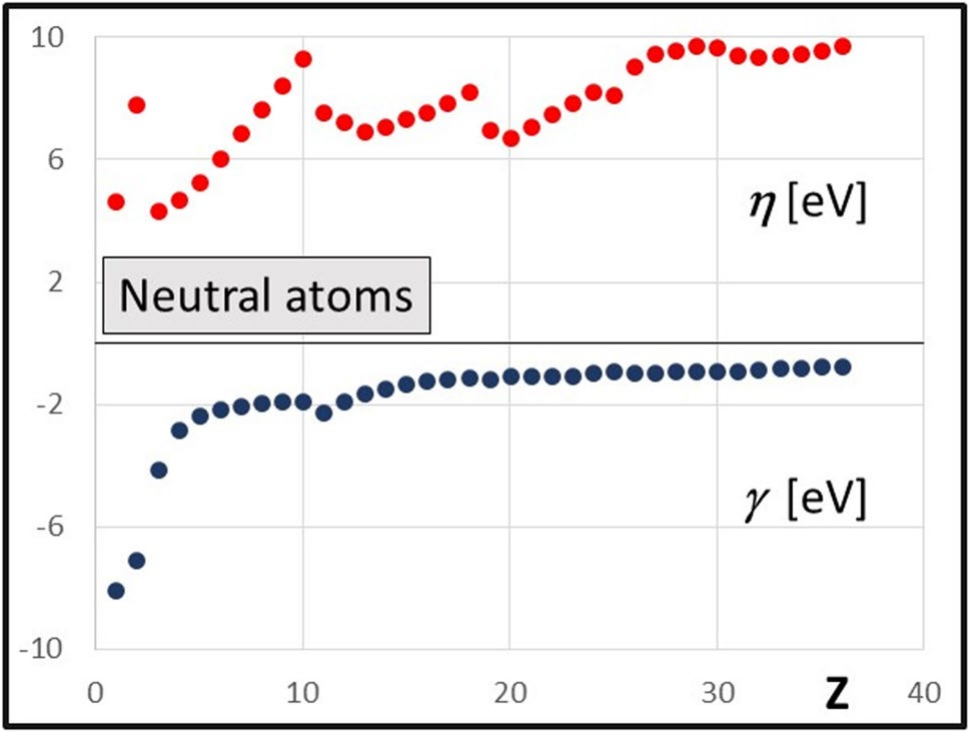
Hardness (*η* = 1/*S*) and hyperhardness (*γ*, Eq. 6) for atoms calculated from electron density in eV

**Fig. 4:**
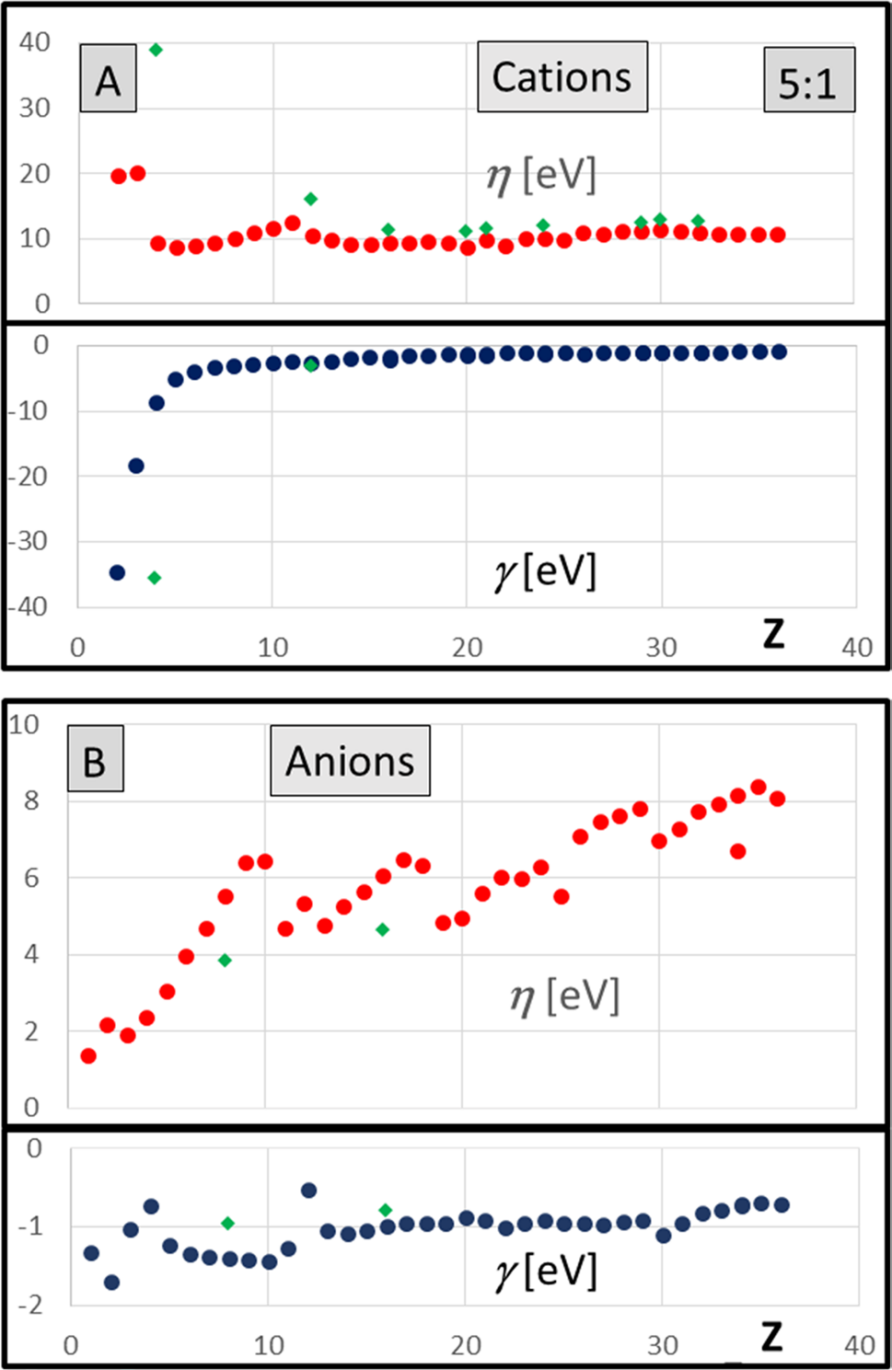
Hardness (*η* = 1/*S*) and hyperhardness (*γ*, Eq. 6) for cations (**A**) and for anions (**B**) in eV. The common di-cations and di-anions have also been marked by the green diamonds

Hyperhardness for atoms and ions is calculated by Eq. 6a. The comparison of hardness (positive values) and hyperhardness (negative values) has been presented as two functions of the atomic number in joint diagrams for 36 neutral atoms (Fig. 3) and for their cations (Fig. 4A) and anions (Fig. 4B). The range on the ordinate axis has been adjusted to expose the details of the diagrams. Several common di-cations and di-anions are also included in Fig. 4A and Fig. 4B, respectively. They provide an additional quick test for the rational character of the results: hardness increasing with further ionization for cations, decreasing for anions.

### Global hypersoftness

**Fig. 5:**
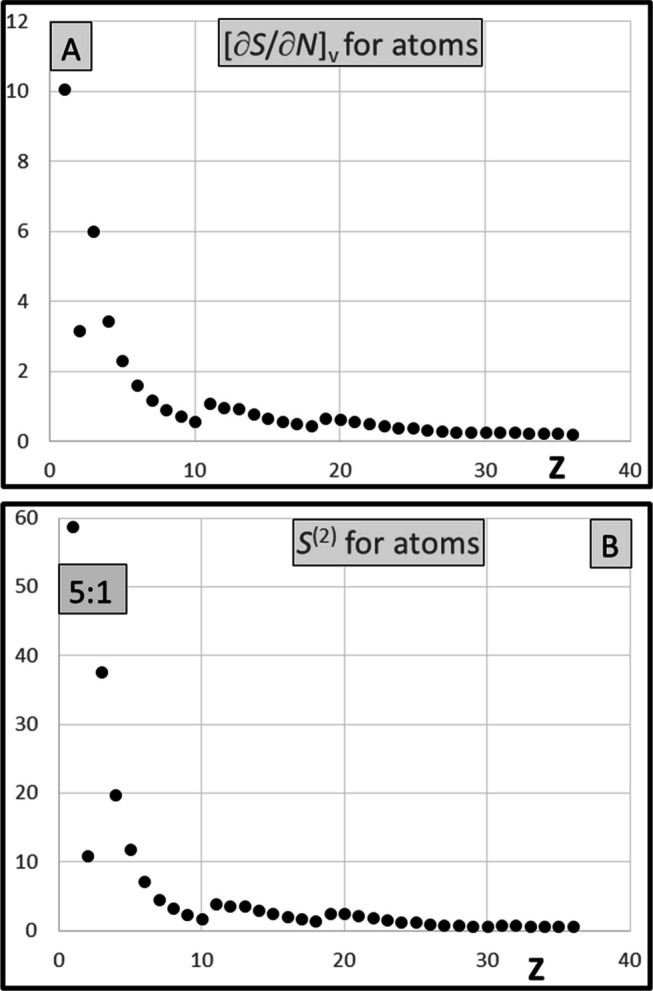
**A** [∂*S*/∂*N*]_*v*_ derivative for atoms in a.u. **B** Global hypersoftness for atoms* S*^(2)^ (Eq. 6. in a.u.). Note the contraction of the ordinate axis by 5:1 ratio

**Fig. 6:**
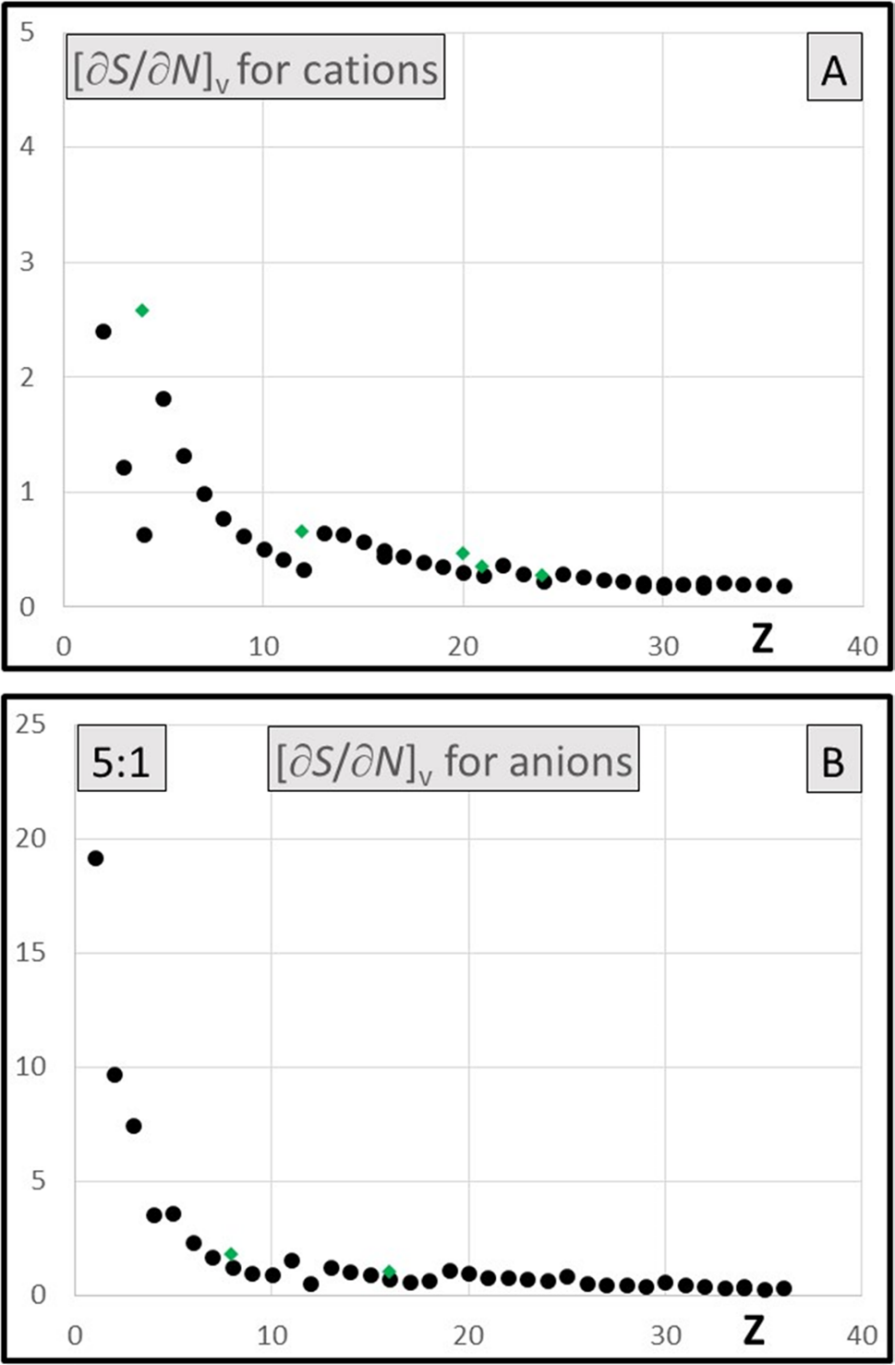
**A** [∂*S*/∂*N*]_*v*_ derivative calculated in a.u. for cations. **B** [∂*S*/∂*N*]_*v*_ derivative calculated in a.u. for anions. Note the contraction of ordinate axis by 5:1 ratio. Results for di-cations (in **A**) and di-anions (in **B**) have also been included (green diamonds) whenever substantially different from the univalent cation

Calculated derivative [∂*S*/∂*N*]_*v*_ has been confronted with the global hypersoftness sensu stricto* S*^(2)^ (Eq. 8) in Fig. 5A and Fig. 5B, respectively. The rough linear correlation between both quantities has been observed for of atoms and cations; the effect results from the interrelation between these two derivatives (Eq. 7). The global softness parameter linking both derivatives spans only modest range of 3 ÷ 6 a.u. for atoms and 1–3 a.u. for cations and it is slowly lowering with increasing *Z*. (In the group of anions, with *S* parameter 5–20 a.u., the effect is considerably less pronounced, though still observed.) For practical reasons, only the derivative [∂*S*/∂*N*]_*v*_ has been presented for cations (Fig. 6A) and for anions (Fig. 6B). Calculated [∂*S*/∂*N*]_*v*_ data for atoms have all been included with the diagrams available in the Supporting Material.

### Variations of the state functions ΔE_N_, Δμ_N_, ΔN_μ_

The local softness $s(r)$, its derivative ${\left[\partial s\left(r\right)/\partial N\right]}_{v}$, and the global softness *S* $\text{available}$ for atoms allow for testing the possible sensitivity of atoms/ions in the model conditions available in the alchemical analysis. Calculations have been limited to elements in rows 1 and 2 of the periodic table, as the well-recognized variations in electronic properties of atoms in this group allow for critical evaluation of the results. The integrals in Eqs. 14–18 have been calculated first (Table 1). In Table 2, the calculated parameters of the parabolic functions of Δ*Z* (*A* and *B* in Eqs. 19–21) have been collected.

**Table 1:** Calculated values of integrals introduced in Eqs. 14–18 (a.u.)

Atom	*Z*	*I*_*ρ*_	*I*_*s*_	*I*_*s2*_	*I*_*N*_	*I*_*N2*_
H	1	1.00	2.86	1.90	0.0259	0.0000237
He	2	3.36	2.76	3.08	0.0353	0.00196
Li	3	5.67	2.74	3.34	0.0141	0.0023
Be	4	8.32	2.72	3.54	0.0123	0.00418
B	5	11.20	2.70	3.70	0.0116	0.0066
C	6	14.37	2.68	3.85	0.0115	0.00964
N	7	17.81	2.66	4.00	0.0118	0.0131
O	8	21.47	2.64	4.14	0.012	0.0167
F	9	25.39	2.62	4.28	0.0122	0.0206
Ne	10	29.56	2.62	4.42	0.0127	0.0244

**Table 2:** Calculated coefficients for the relations: $\Delta {E}_{N}(\Delta Z)$, $\Delta {\mu }_{N}\left(\Delta Z\right),$ $\Delta {N}_{\mu }\left(\Delta Z\right)$ in Eqs. 19–21 (a.u.). The necessary values for integrals are listed in Table 1; global softness *S* and hyperhardness *γ* are from the table in the Appendix

Atom	Δ*E*_*N*_ ${=A}_{E}\Delta Z+{B}_{E}\Delta {Z}^{2}$ (Eq. 19)		Δ*μ*_*N*_ ${=A}_{\mu }\Delta Z+{B}_{\mu }\Delta {Z}^{2}$ (Eq. 20)		Δ*N*_*μ*_ ${=A}_{N}\Delta Z+{B}_{N}\Delta {Z}^{2}$ (Eq. 21)	
	${A}_{E}$	${B}_{E}$	${A}_{\mu }$	${B}_{\mu }$	${A}_{N}$	${B}_{N}$
H	− 1.00	1.26	− 0.490	33.0	2.86	0.0000690
He	− 3.36	2.90	− 0.793	27.0	2.76	0.00341
Li	− 5.67	2.56	− 0.436	15.5	2.74	0.00723
Be	− 8.32	2.86	− 0.471	10.4	2.72	0.0120
B	− 11.2	3.18	− 0.524	8.61	2.70	0.0170
C	− 14.4	3.57	− 0.596	7.73	2.68	0.0217
N	− 17.8	4.02	− 0.673	7.20	2.66	0.0259
O	− 21.5	4.48	− 0.742	6.85	2.64	0.0297
F	− 25.4	4.98	− 0.814	6.58	2.62	0.0332
Ne	− 29.6	5.56	− 0.897	6.44	2.62	0.0356

## Discussion

**Fig. 7:**
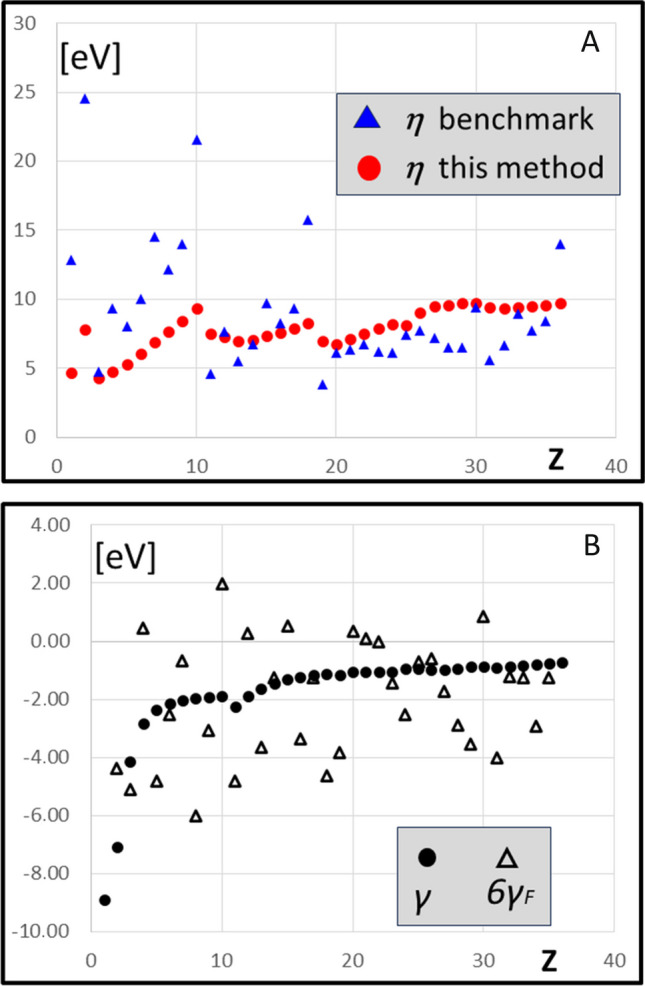
**A** Hardness of atoms *η* = *S*^−1^ (eV) calculated by this method (ref. 25) as compared to the benchmark values (ref. 76). **B** Hyperhardness of atoms (eV): *γ—*this work; 6*γ*_*F*_ by Fuentealba and Parr (ref. 40)

The results presented in this work are all founded on the extension of our gradient theorem (Eq. 1 and Eq. 2c). On the first step of this work, the softness of atoms and ions has been calculated from the electron density only. This is well in contrast to the standard method introduced by Parr and Pearson and founded on the energy parameters exclusively. The efforts to find another source of the atomic hardness parameters have long history [68–70]. The problem has been analyzed in depth by Ayers [74]; the old relations between the size and polarizability and softness/hardness have been reminded and many more arguments have been raised, leading to the conclusion that the original concept *η* ∝ *I-A* might be reconsidered. A solution has been proposed in the following papers by Cardenas et al. who introduced the benchmark values of the chemical hardness for atoms [75, 76]. These values are also based on the energy parameters exclusively; nevertheless, they represent most reasonable set of data to which our results might be compared. This has been demonstrated in Fig. 7A in the *η*(*Z*) relationship that shows the asymptotic behavior of the index resulting from this work. The change in hardness of atoms in rows 1 and 2 is parallel; in periods 3 and 4, this trend is less pronounced, but still visible. The turning points at completion of the electronic shells are reproduced in both approaches. In period 4 (*Z* > 18), the hardness parameters from the density (this method) are gradually increasing, while the benchmark data undergo stepwise variations with no actual explanation.

The review of global hardness data (*η*) for atomic cations and anions, as compared to those for atoms, provides the support for the coherence of the results (Fig. 3 and Fig. 4). The anions are all considerable softer that the neutral atoms (the di-anions are even softer still), while cations are all harder. With the increase of the atomic number, the difference between hardness of ions vs atoms is lowered.

The pioneering results for hyperhardness by Fuentealba and Parr ${\gamma }_{F}={1/6\left[{\partial }^{3}E/{\partial N}^{3}\right]}_{v}$ have been obtained by the sophisticated interpolation based on three experimental parameters: electron affinity *A*_1_ and ionization energies *I*_1_ and *I*_2_ [40]. The hyperhardness *γ*_*F*_ was found to be negative, except for the atoms in their *S* electronic state (e.g., noble gases), where exceedingly small, positive ${\gamma }_{F}>0$ was found. While no correlation between the present results (*γ*) and 6 ${\gamma }_{F}$ was observed (Fig. 7B), both values span over similar numerical data with neatly equivalent average values: < 6*γ*_*F*_ >  =  − 1.86 eV, < *γ* >  =  − 1.77 eV. This observation is meaningful, given the much different origin of the two sets of data. The notable advantage of the method applied hereby is rather regular change in *γ*(*Z*) function equally for neutral atoms (Fig. 3) and their ions (Fig. 4) whereas the dispersed ${6\gamma }_{F}(Z)$ data could not be fitted with any feasible rational interpolation (Fig. 7B).

The applied method of calculation of the global energy derivatives (*η*, *γ*) from the properties of the stationary electron density alone represents an explicit example that these indices are density functionals and that electron density is the basic variable in cDFT. The well-known problems with the degeneracy of the electronic states that invalidates the usual reactivity indicators associated with the cDFT have been circumvented [77, 78]. Although this approach leads merely to a lower bound to the response parameters, the simple availability of the profound DFT descriptor may be valuable for the chemical practice, once the phenomenological method of their use is available in calculation of the variations of the state functions $\Delta {E}_{N}$, $\Delta {\mu }_{N}$, $\Delta {N}_{\mu }$. Recent efforts by Miranda-Quintana et al. may bring a fresh interest in this matter [79, 80], and the problem of indexing the chemical properties by the density-based derivatives has also been focused upon in other recent papers [81, 82]. The interplay between the electric field and the electron density function has been analyzed in detail in the classical work by Liu and Hunt, where the set of new connections between molecular softness and the electromagnetic properties of molecules has been analyzed in detail [83]. Specifically, the role of the local softness and hypersoftness to long-range intermolecular forces has been exposed.

The hyperhardness calculated in the framework of the local approximation for atoms increases along the rows of the periodic table, but it tends to be rather smooth flat function of the atomic numbers for *Z* > 10 within their periods. The hardness data from the same method show distinct discontinuities at critical points for the electron number, corresponding to complete filling consecutive electronic shells (Fig. 3: *Z* = 2, 10, 18, 25). The hyperhardness for cations (Fig. 4A) is smaller as compared to the hardness of atoms except the lightest atoms. The contribution from hyperhardness may have considerable effect for anions (Fig. 4B), with absolute value of hyperhardness *γ* reaching ca. 10% of hardness itself.

The numerical values of the local function ${[\partial s\left(r\right)/\partial N]}_{v}$ for light atoms (*Z* = 1 ÷ 10) are considerably smaller than the values of the local softness $s\left(r\right)$ and their orbital structure is less exposed (cf. Supporting Material). For heavier atoms (*Z* > 10), the derivative ${[\partial s\left(r\right)/\partial N]}_{v}$ becomes negligibly small as compared to the local softness. Notably, they span much broader region around atoms than the local softness functions and their role for many anions may not be neglected; complete integration of these functions could not be achieved in the standard grid size [0 ÷ 20 a. u.].

The alchemical approach methodology [84–86] allows for assessing the role of softness and hypersoftness for the energy *E* and chemical potential *μ* for a closed system (polarization effect) and for the possible flow of charge $\Delta {N}_{\mu }$ to/from an atom and a reservoir at constant *μ* (an open system), the handy model for an electron exchange in a chemical reaction. The role of derivatives representing the susceptibilities of atoms to a disturbance of the external potential has been determined, by calculating the integrals involved in the expansion (Eqs. 14–18 and Table 1). Integrals *I*_*N*_ and *I*_*N2*_ involving the local function ${[\partial s\left(r\right)/\partial N]}_{v}$ (Eq. 17 and Eq. 18) are small as compared with other entries in this table. They appear to have little effect for the state functions, as their role for the coefficient *B*_*μ*_ and *B*_*N*_ is marginal. However, the disturbance in the second order cannot be entirely neglected, except for $\Delta {N}_{\mu }(\Delta Z)$ (Eq. 21). Calculated parameters for the $\Delta {E}_{N}\left(\Delta Z\right)\; \text{and}\; \Delta {\mu }_{N}\left(\Delta Z\right)$ relationships presented in Table 2 prove the role of the second-order derivatives, since the local softness *s*(*r*) plays quite significant role here. The second-order effect provides large term of the energy change and has been proved to dominate the effect *Δμ* in the chemical potential (at constant *N*). Only for the charge flow (at constant *μ*) it is limited to the first-order effect.

The local approximation systematically explored in this work allows also to estimate the role of the third-order effects on the energy Δ*E*_*N*_, associated with the disturbance of the electric field on the electron density of an atom. The third derivative of the energy *E*[*v*(**r**), *N*] over *v* is transformed to the density derivative, as demonstrated in the work by Heidar-Zadeh et al. [43] ${E}^{{\prime}{\prime}{\prime}}={\left[\frac{\delta \omega ({r}_{1{\prime}}{r}_{2})}{\delta v({r}_{3})}\right]}_{N}$. By exploring the Berkowitz and Parr relation followed by the local approximation, the final result for Δ^(3)^*E*_*N*_ effect involves the same set of integrals as listed in Eqs. 15–18, with a dominant role of the *I*_*N*_ and *I*_*N2*_ that have been proved to play only the minor role (Table 1). The complete result is presented in the Appendix. At the level of alchemical approach adopted in this work, including the third-order correction to the energy expansion leads to the supplemented Δ*E*_*N*_(Δ*Z*) function of the second order.

The characteristic feature of the $\Delta N(\Delta Z)$ relationship represented by its slope for the limit of $\Delta Z\to 0$ was found to be unique for every orbital $\Delta N/\Delta Z\to 3 \text{a}.\text{u}.$ [35]. The results for actual atoms were found reasonably close to this prediction $\Delta N/\Delta Z=>{A}_{N}$ (Table 2), with 2.62 < *A*_*N*_ < 2.86 (a.u.). The difference could be attributed to the effect of electron–electron interaction, as the prediction for orbitals was for single occupied orbitals only.

These results help to understand the effects of the actual reactions in some indirect way. In the alchemical method, the disturbing potential leading to changes in a system originates from a hypothetical change $\Delta Z$ that occurs at the center of symmetry of the electron density. The response to this disturbance involves preferentially the inner part of the electron density [55]. This is clearly observed by the integrals *I*_*ρ*_ in Table 1: 99% of their values come from the electron density within a sphere of radius not exceeding 5 a.u. (Li). In a real situation, the disturbing potential acts from a distance to an atom or molecule and the outer sphere of the electron density is affected. In this region, the contribution from the local softness *s*(*r*) will also be dominating the consequences of such disturbances. The role of hypersoftness ${[\partial s\left(r\right)/\partial N]}_{v}$ that contributes to the integrals *I*_*N*_ and *I*_*N2*_ (Eq. 17 and Eq. 18) will be exposed by the strengths of the external field dominating in the outer sphere of an atom and by the large volume of integration. The effects of confinement on a reaction are evident field for testing the role of this parameter [87]. This may be especially important for calculation of the chemical effect given by the charge flow (at constant *μ*) $\Delta {N}_{\mu }$, hence reaching further than the predictions exploring the dual descriptor, defined for the closed system (canonical ensemble).

Calculated derivatives of the local softness [∂*s*(**r**)/∂*N*]_*v*_ allow also for calculation of the new version of the double descriptor *f*^(2)^(**r**) via Eq. 10. Results for *f*^(2)^(**r**) in atoms in the 2nd and 3rd periods are demonstrated in Figs. 1B and 2B. The results are systematically negative at short distances from a nucleus and positive at the outer region around a nucleus; they fall to zero at infinity and they do integrate to zero, as required. However, unlike the original double finite-difference quantities Δ*f*(**r**) [48], the local derivatives *f*^(2)^(**r**) themselves are not expected to reflect the susceptibility of an atom to a nucleophilic or an electrophilic attack, neither by their sign, nor by their value, as it has been claimed for Δ*f*(**r**) descriptors in the original work of the authors [49]. The potential prognostic value of the present method is limited to the observables calculated for a given perturbance of the electric field in the space of an atom in electronic ground state: Δ*E*_*N*_—interaction energy at constant *N*; Δ*μ*_*N*_—the change in the chemical potential at constant *N*; Δ*N*_*μ*_—the modification of the population at constant chemical potential.

The fundamental requirement introduced by Eq. 2c is that the density gradient vector and electric field vector are parallel in every point in space. This condition is obviously fulfilled for free atoms and their ions. According to our preliminary computations, this condition is also met for the valence region of a molecule. On the other hand, the gradient theorem (Eq. 1) holds only for the density resulting from computational methods that comply to both Hellmann–Feynman theorem and Feynman electrostatic expression for atomic force [13]. The vital role of this condition for the accuracy of the quantum chemical computations has been discovered and discussed in other works from this laboratory [88, 89].

## Conclusion

The method described in this paper opens a new way to exploration of the vital cDFT derivatives: the linear response function $\omega \left(\mathbf{r},{\mathbf{r}}^{\mathbf{^{\prime}}}\right)$ and the softness kernel $s\left(\mathbf{r},{\mathbf{r}}^{\mathbf{^{\prime}}}\right)$. Applications $\omega \left(\mathbf{r},{\mathbf{r}}^{\mathbf{^{\prime}}}\right)$ in chemistry have been subject of many efforts [90, 91], hampered by the nonlocal character of this derivative. The Fukui function *f*(**r**) and the dual descriptor* f*^(2)^(**r**), another energy derivative of the third order, have been found more promising for chemical use, due to their local character [51, 53]. This present work has demonstrated that the non-local response functions may be replaced (to some extent) by exploring the local derivatives of higher order. This resulted not only in calculations of the local descriptors, but also the method for their phenomenological use has been proposed: calculation of the disturbances of the state functions $\Delta {E}_{N}$, $\Delta {\mu }_{N}$, $\Delta {N}_{\mu }$. While the first two describe merely the polarization of a system due to the external potential, $\Delta {N}_{\mu }$ describes the truly chemical effect: the charge flow. The nonlocal kernels $\omega \left(\mathbf{r},{\mathbf{r}}^{\mathbf{^{\prime}}}\right)$ and $s\left(\mathbf{r},{\mathbf{r}}^{\mathbf{^{\prime}}}\right)$ have not been neglected but replaced by the higher order local derivative: $[\partial s(\mathbf{r})/\partial {N]}_{v}$. Calculation thereof was then necessary for the completeness of the method.

The novel local approach for calculation of the basic derivative of the local softness $[\partial s(\mathbf{r})/\partial {N]}_{v}$ enables to derive analytical formulas for cDFT reactivity indices of higher order. The integral of $[\partial s(\mathbf{r})/\partial {N]}_{v}$ gives the meaningful positive value of $[\partial S/\partial {N]}_{v}$ that opens an access to the canonical global energy derivatives of the third order: the hyperhardness (Eq. 22a) and the hypersoftness (Eq. 22b):

22a $$\gamma {=\left[{\partial }^{3}E/\partial {N}^{3}\right]}_{v}=-{S}^{-2}[\partial S/\partial {N]}_{v}$$

22b $${{S}^{(2)}=\left[{\partial }^{3}\Omega /\partial {\mu }^{3}\right]}_{v}=S[\partial S/\partial {N]}_{v}$$

Calculated data for *γ* in atoms have generally reproduced the first suggested results obtained by the inconsistent extrapolation of the energy function *E*(*N*) for atoms; the difficulties and arbitrary assumptions of the former method have been avoided. The radial distribution of $[\partial s(r)/\partial {N]}_{v}$ derivative (integrated to positive global $[\partial S/\partial {N]}_{v}$) is characteristically different from the analogous function that has also been calculated: the dual descriptor *f*^(2)^ (integrated to zero). The detailed results are presented in the Supporting Material available with this paper.

Application of 1st and 2nd density derivatives $s(\mathbf{r})$ and $[\partial s(\mathbf{r})/\partial {N]}_{v}$ confirmed the vital role of the local softness *s*(**r**) for a response of the energy $(\Delta {E}_{N})$ and the chemical potential $(\Delta {\mu }_{N})$ of an object (at constant *N*) to perturbation by an external potential $\Delta v(\mathbf{r})$, and also for the electron flow between an atom and an external reservoir (at constant *μ*)—$\Delta {N}_{\mu }$. The tiny contributions from hypersoftness in these studies are well understood at the level of the alchemical analysis; it may play a role in studies focused on the outer space of an atom. Calculated results for the state functions represent the promising application for the results of the axiomatic approach.

## Supplementary Information

Below is the link to the electronic supplementary material.

Supplementary file1 The visual collection containing the radial distribution diagrams is available in form of the pdf presentation. It contains visualization of the derivatives: *s*(*r*),${\left[ds\left(r\right)/dN\right]}_v$ ,and   *f*(*r*), $f^{\left(2\right)}\;\left(r\right)$ for 36 atoms in rows 1÷4 of the periodic table; their cations and anions (selected di-cations and di-anions) have also been included. (PDF 4602 KB)

## Data Availability

No datasets were generated or analysed during the current study.

## Appendix

The third-order component to the energy change Δ^(3)^*E*_*N*_ under the rigor exposed in ref. 43 is:

A.1 $${\Delta }^{(3)}{E}_{N}=\int \int {\int \left[\frac{\delta \omega ({\mathbf{r}}_{1},{\mathbf{r}}_{2})}{\delta v({\mathbf{r}}_{3})}\right]}_{N}\Delta v({\mathbf{r}}_{1})\Delta v({\mathbf{r}}_{2})\Delta v({\mathbf{r}}_{3})d{\mathbf{r}}_{1}d{\mathbf{r}}_{2}d{\mathbf{r}}_{3}$$

Exploring the Berkowitz and Parr relation and the local approximation allows for transformation of Eq. A.1 leads to:

$${\Delta }^{(3)}{E}_{N}=-\int {\int \left[\frac{\delta s({\mathbf{r}}_{1})}{\delta v({\mathbf{r}}_{3})}\right]}_{N}{\left[\Delta v({\mathbf{r}}_{1})\right]}^{2}\Delta v({\mathbf{r}}_{3})d{\mathbf{r}}_{1}d{{\varvec{r}}}_{3}+$$

A.2 $$+\int \int {\int \left[\frac{\delta \{Sf({{\varvec{r}}}_{1})f{({\varvec{r}}}_{2})\}}{\delta v({{\varvec{r}}}_{3})}\right]}_{N}\Delta v({\mathbf{r}}_{1})\Delta v({\mathbf{r}}_{2})\Delta v({{\varvec{r}}}_{3})d{\mathbf{r}}_{1}d{\mathbf{r}}_{2}d{{\varvec{r}}}_{3}$$

The following expansions are available under the cDFT scrutiny for the derivatives to be integrated: [92]

A.3 $${\left[\frac{\delta s({\mathbf{r}}_{1})}{\delta v({\mathbf{r}}_{3})}\right]}_{N}=-s({\mathbf{r}}_{1}){\left[\frac{\partial s({\mathbf{r}}_{3})}{\partial N}\right]}_{v}+f({\mathbf{r}}_{1})s({\mathbf{r}}_{3}){\left[\frac{\partial S}{\partial N}\right]}_{v}+S{\left[\frac{\partial \omega ({\mathbf{r}}_{1},{\mathbf{r}}_{3})}{\partial N}\right]}_{v}$$

A.4 $$\begin{array}{c}{\left\{\frac{\delta [Sf({\mathbf{r}}_{1})f{(\mathbf{r}}_{2})]}{\delta v({\mathbf{r}}_{3})}\right\}}_{N}=-f({\mathbf{r}}_{1})f{(\mathbf{r}}_{2})S{\left[\frac{\partial s({\mathbf{r}}_{3})}{\partial N}\right]}_{v}+s({\mathbf{r}}_{3}){\left[\frac{\partial S}{\partial N}\right]}_{v}+\\ \qquad \qquad \qquad \qquad \quad+Sf{(\mathbf{r}}_{2}){\left[\frac{\partial \omega ({\mathbf{r}}_{1},{\mathbf{r}}_{3})}{\partial N}\right]}_{v}+Sf({\mathbf{r}}_{1}){\left[\frac{\partial \omega ({\mathbf{r}}_{2},{\mathbf{r}}_{3})}{\partial N}\right]}_{v}\end{array}$$

The integration of the derivative of the linear response function over *N* has been presented in our earlier work [35].

A.5 $$\int {\int \left[\frac{\partial \omega (\mathbf{r},\mathbf{r}^{\prime})}{\partial N}\right]}_{v}\Delta v(\mathbf{r})\Delta v(\mathbf{r}^{\prime})d\mathbf{r}d\mathbf{r}^{\prime}=\left\{\begin{array}{c}-\int {\left[\frac{\partial s(\mathbf{r})}{\partial N}\right]}_{v}{\left[\Delta v(\mathbf{r})\right]}^{2}d\mathbf{r}+\\ +\gamma {\left[\int s(\mathbf{r})\Delta v(\mathbf{r})d\mathbf{r}\right]}^{2}+\\ +2\eta \int {\left[\frac{\partial s(\mathbf{r})}{\partial N}\right]}_{v}\Delta v(\mathbf{r})d\mathbf{r}\int s(\mathbf{r}^{\prime})\Delta v(\mathbf{r}^{\prime})d\mathbf{r}^{\prime}\end{array}\right\}$$

With the use of Eqs. A.3, A.4, and A.5, the integration in Eq. A.1 contains the single integrals exclusively, with the integrands provided by the softness (local and global) and their derivatives over *N*, now avaiable by the method presented in this paper.
