# Supplementary material for: Hyperhardness and hypersoftness of atoms and their ions
Source: J Mol Model. 2024 Sep 21;30(10):344. doi: 10.1007/s00894-024-06136-3 (PMC11416423; doi:10.1007/s00894-024-06136-3)
Supplement: Supplementary file 1 — Supplementary file1 The visual collection containing the radial distribution diagrams is available in form of the pdf presentation. It contains visualization of the derivatives: s(r),${\left[ds\left(r\right)/dN\right]}_v$dsr/dNv ,and f(r), $f^{\left(2\right)}\;\left(r\right)$f2r for 36 atoms in rows 1÷4 of the periodic table; their cations and anions (selected di-cations and di-anions) have also been included. (PDF 4602 KB) [file 894_2024_6136_MOESM1_ESM.pdf]

Softness and hypersoftness of atoms and their ions

Graphical presentation of the radial distribution

H																	He
Li	Be											B	C	N	O	F	Ne
Na	Mg											Al	Si	P	S	Cl	Ar
K	Ca	Sc	Ti	V	Cr	Mn	Fe	Co	Ni	Cu	Zn	Ga	Ge	As	Se	Br	Kr

Click on the element symbol

Presentation pattern (all data in [a.u.])

$s(r)$ and $[\mathrm{d}s(r)/\mathrm{d}N]_v$ for a cation	$s(r)$ and $[\mathrm{d}s(r)/\mathrm{d}N]_v$ for an atom	$s(r)$ and $[\mathrm{d}s(r)/\mathrm{d}N]_v$ for an anion
$f(r)$ and $[\mathrm{d}f(r)/\mathrm{d}N]_v$ for a cation	$f(r)$ and $[\mathrm{d}f(r)/\mathrm{d}N]_v$ for an atom	$f(r)$ and $[\mathrm{d}f(r)/\mathrm{d}N]_v$ for an anion
Selected di-cations and di-anions have also been included on separate panels		

J. Zaklika, P. Ordon , L. Komorowski, Hyperhardness and hypersoftness of atoms and their ions,

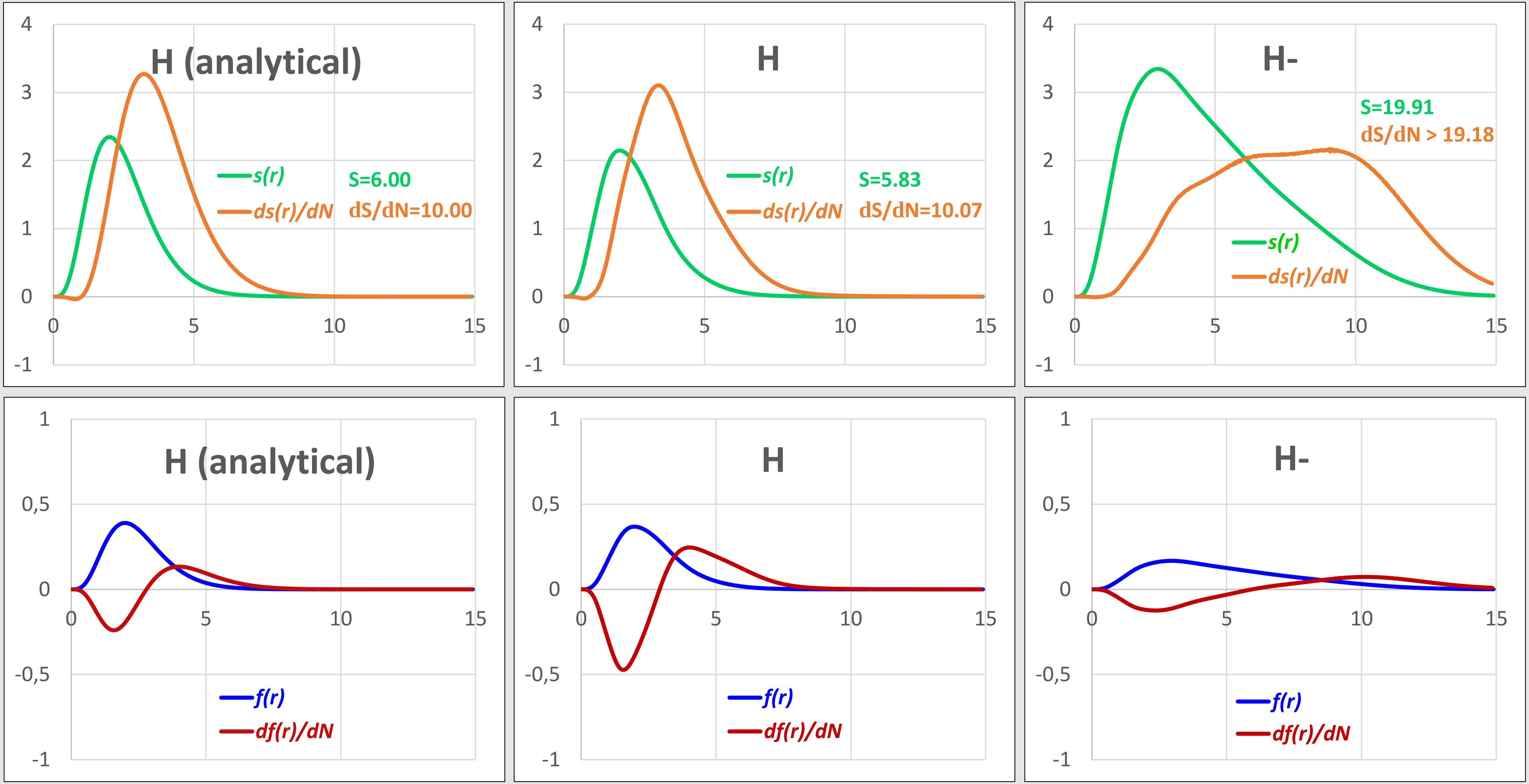

ELEMENTS

J. Zaklika, P. Ordon , L. Komorowski, Hyperhardness and hypersoftness of atoms and their ions,

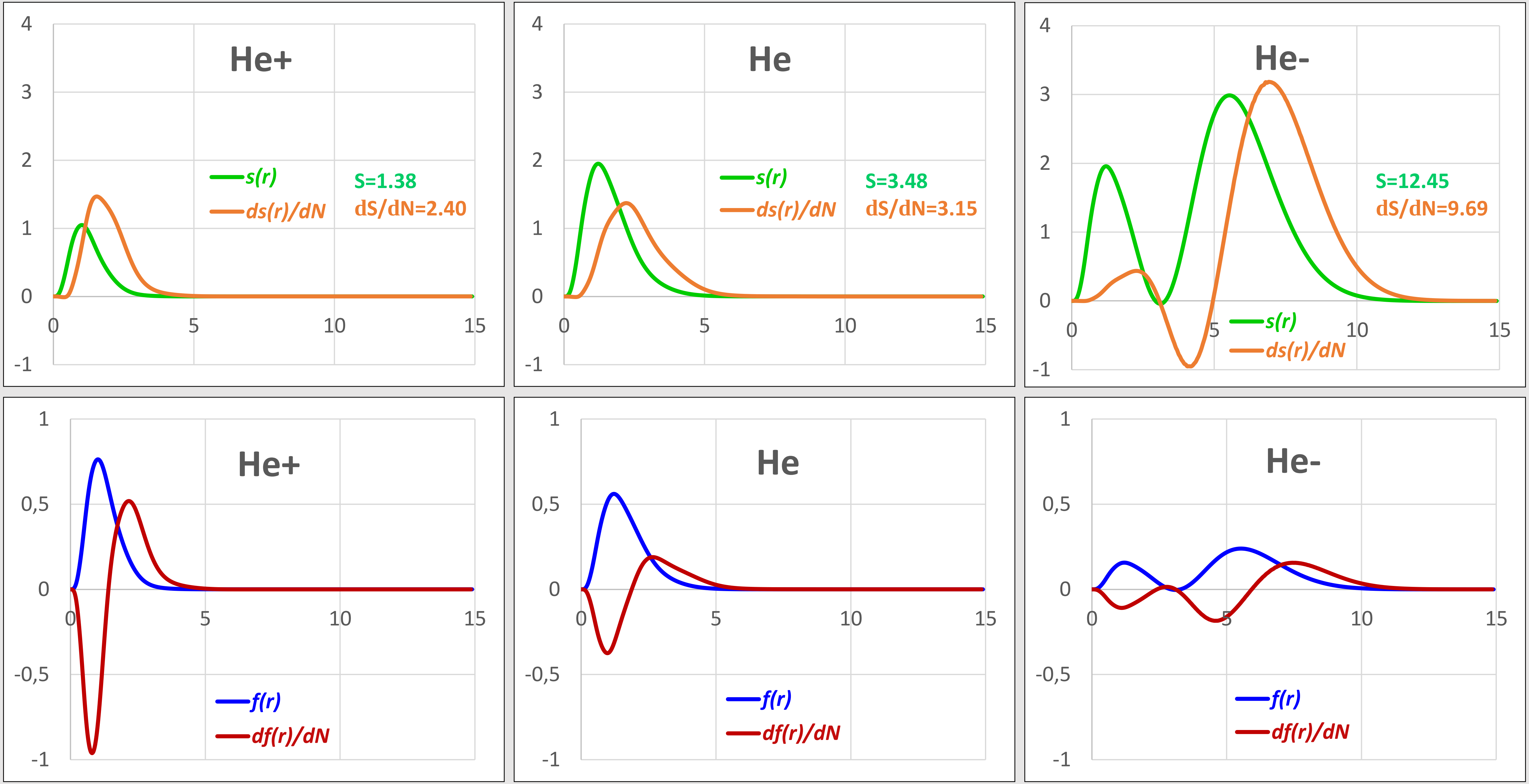
He+
s(r)
ds(r)/dN
S=1.38
dS/dN=2.40
He
s(r)
ds(r)/dN
S=3.48
dS/dN=3.15
He-
s(r)
ds(r)/dN
S=12.45
dS/dN=9.69
He+
f(r)
df(r)/dN
He
f(r)
df(r)/dN
He-
f(r)
df(r)/dN

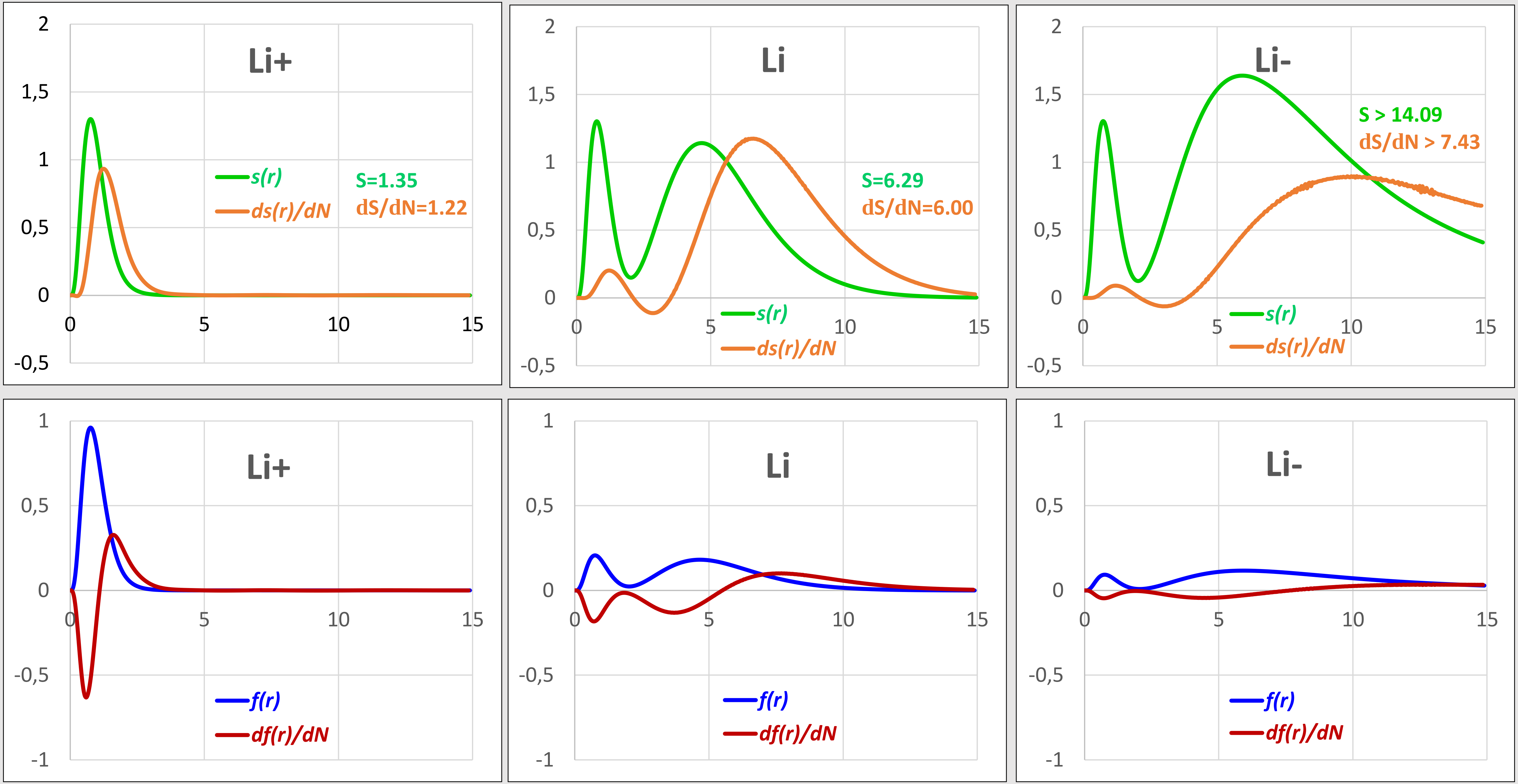
Li+
s(r)
ds(r)/dN
S=1.35
dS/dN=1.22
Li
s(r)
ds(r)/dN
S=6.29
dS/dN=6.00
Li-
S > 14.09
dS/dN > 7.43
s(r)
ds(r)/dN
Li+
f(r)
df(r)/dN
Li
f(r)
df(r)/dN
Li-
f(r)
df(r)/dN

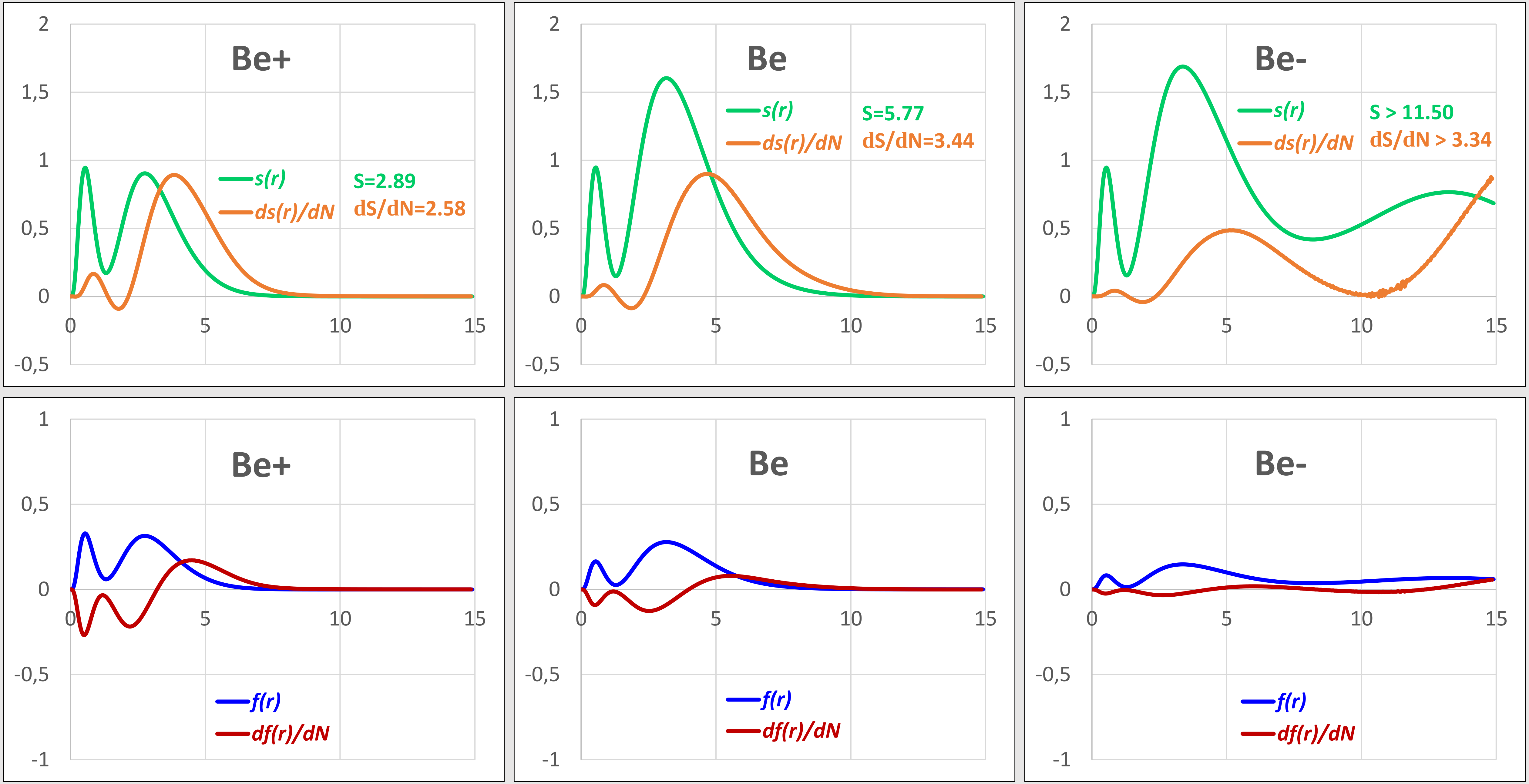
Be+
s(r)
ds(r)/dN
S=2.89
dS/dN=2.58
Be
s(r)
ds(r)/dN
S=5.77
dS/dN=3.44
Be-
s(r)
ds(r)/dN
S > 11.50
dS/dN > 3.34
Be+
f(r)
df(r)/dN
Be
f(r)
df(r)/dN
Be-
f(r)
df(r)/dN

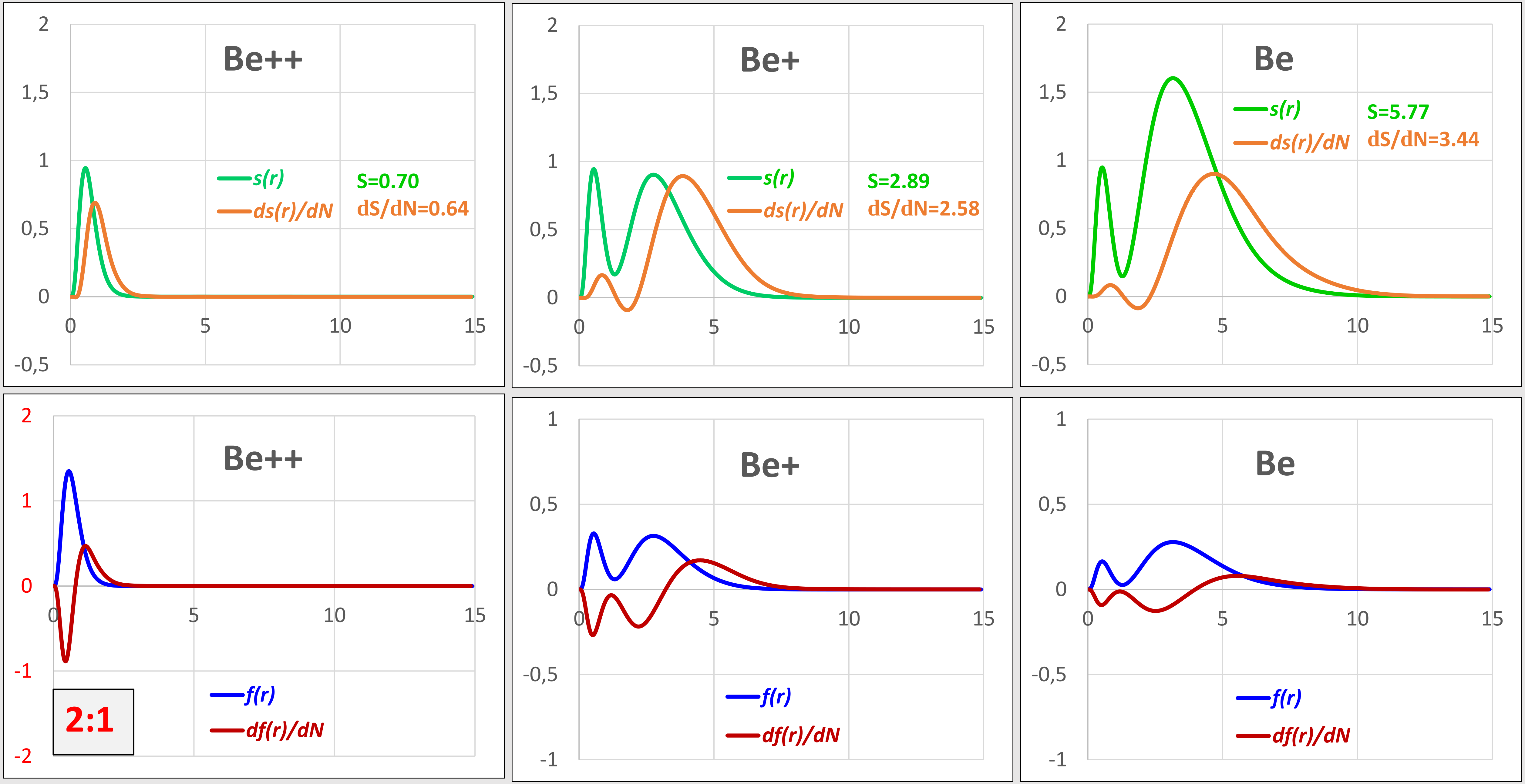

J. Zaklika, P. Ordon , L. Komorowski, Hyperhardness and hypersoftness of atoms and their ions,

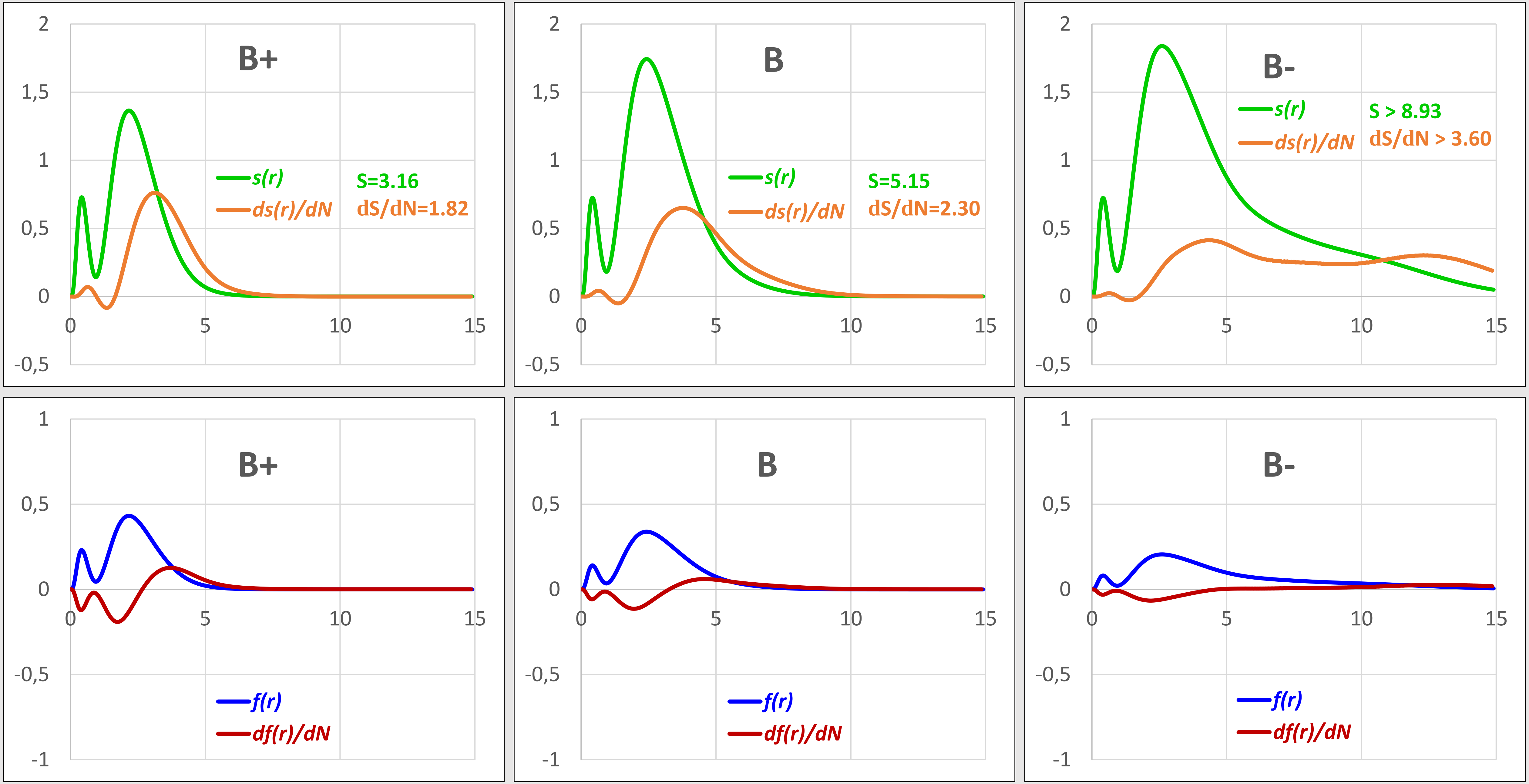
B+
s(r)
ds(r)/dN
S=3.16
dS/dN=1.82
B
s(r)
ds(r)/dN
S=5.15
dS/dN=2.30
B-
s(r)
ds(r)/dN
S > 8.93
dS/dN > 3.60
B+
f(r)
df(r)/dN
B
f(r)
df(r)/dN
B-
f(r)
df(r)/dN

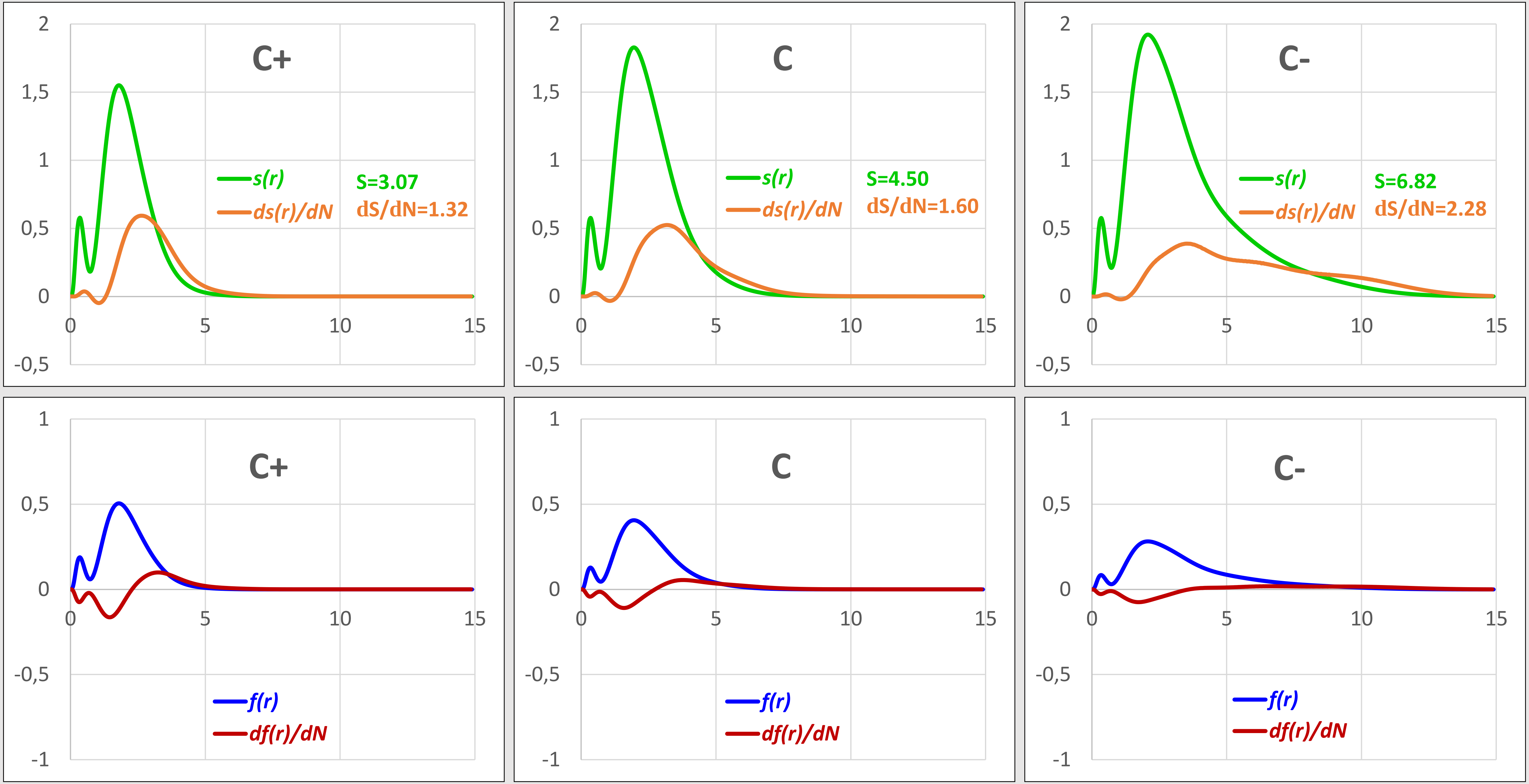

C+
s(r)
ds(r)/dN
S=3.07
dS/dN=1.32
C
s(r)
ds(r)/dN
S=4.50
dS/dN=1.60
C-
s(r)
ds(r)/dN
S=6.82
dS/dN=2.28
C+
f(r)
df(r)/dN
C
f(r)
df(r)/dN
C-
f(r)
df(r)/dN

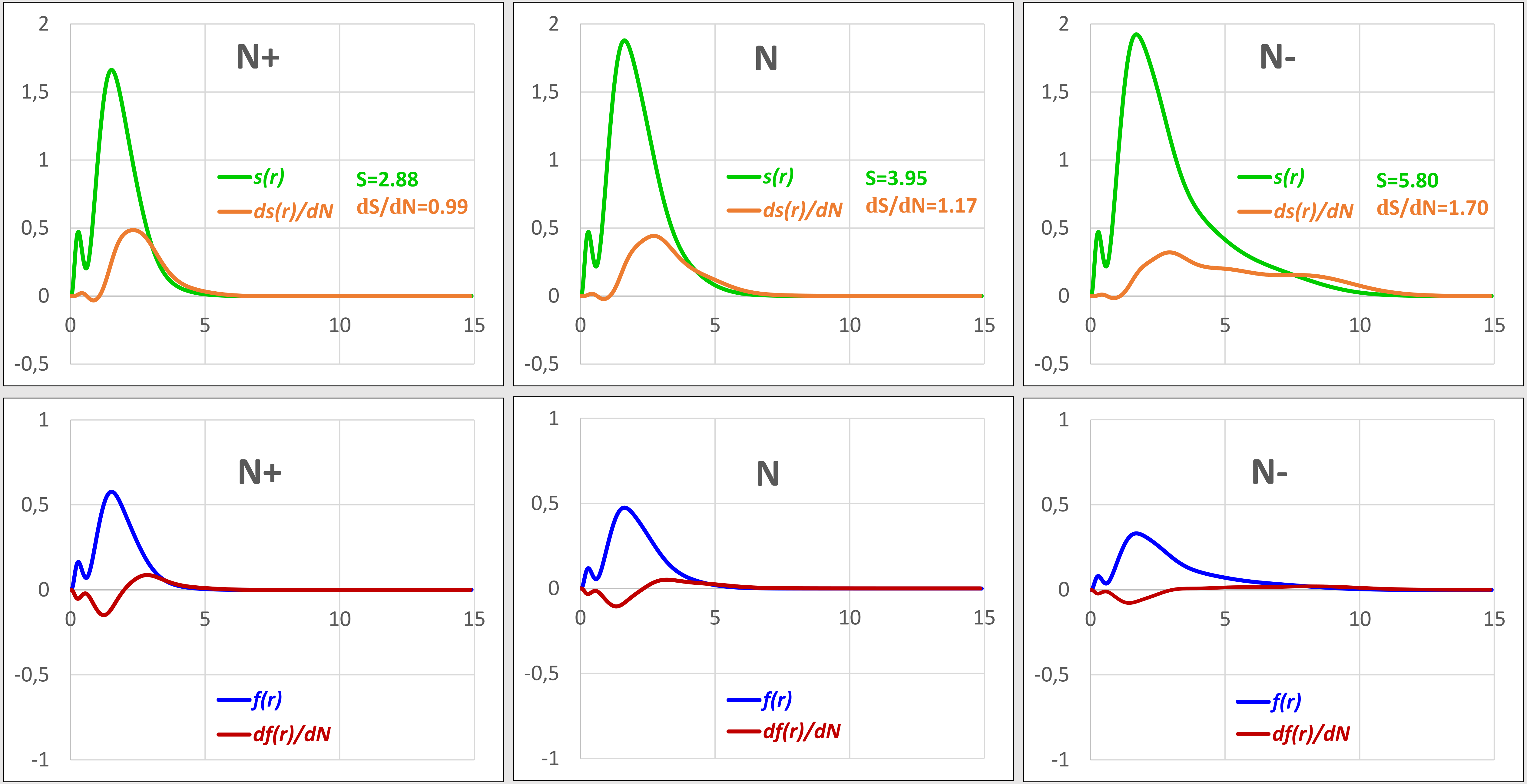

J. Zaklika, P. Ordon , L. Komorowski, Hyperhardness and hypersoftness of atoms and their ions

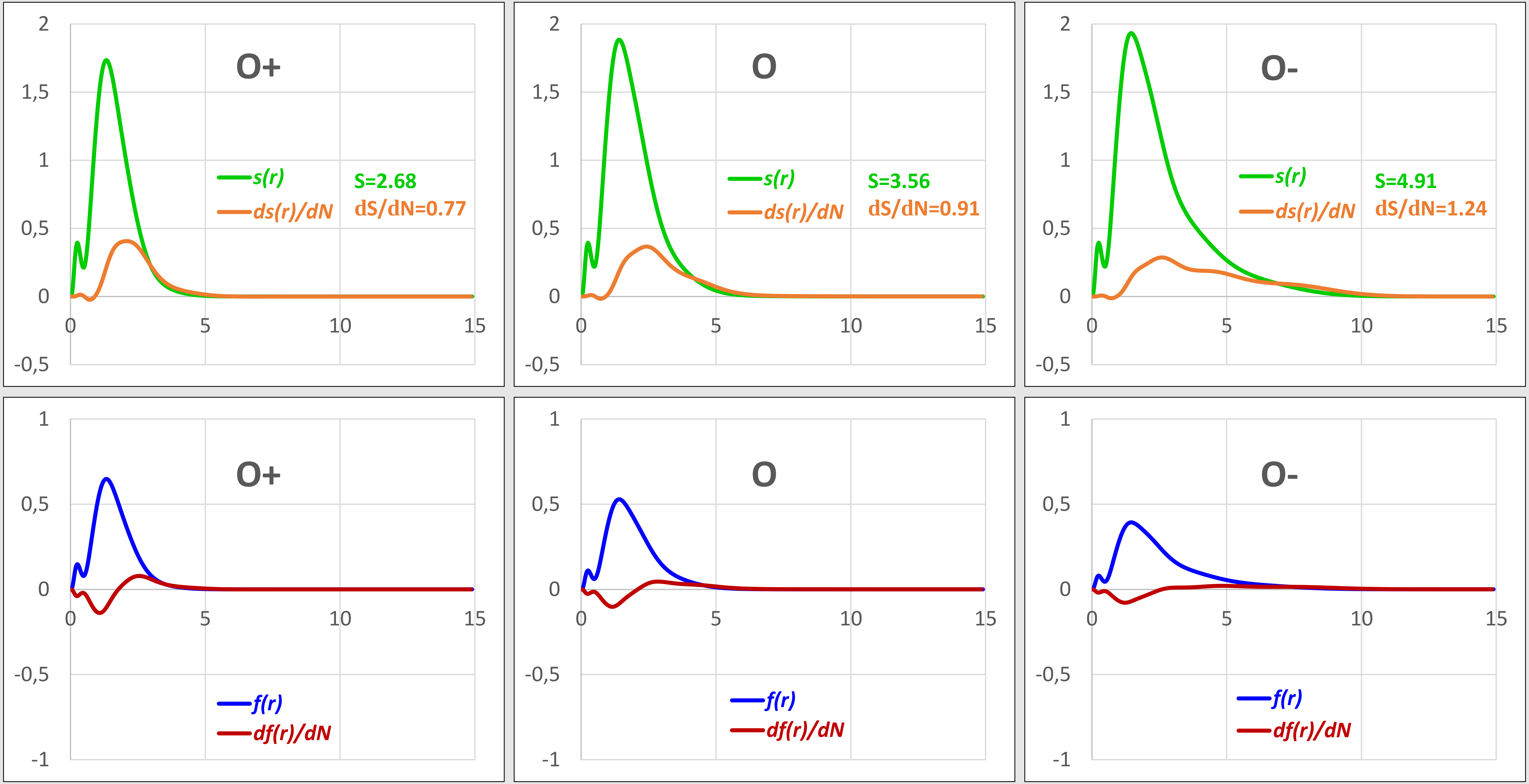
O+
s(r)
ds(r)/dN
S=2.68
dS/dN=0.77
O
s(r)
ds(r)/dN
S=3.56
dS/dN=0.91
O-
s(r)
ds(r)/dN
S=4.91
dS/dN=1.24
O+
f(r)
df(r)/dN
O
f(r)
df(r)/dN
O-
f(r)
df(r)/dN

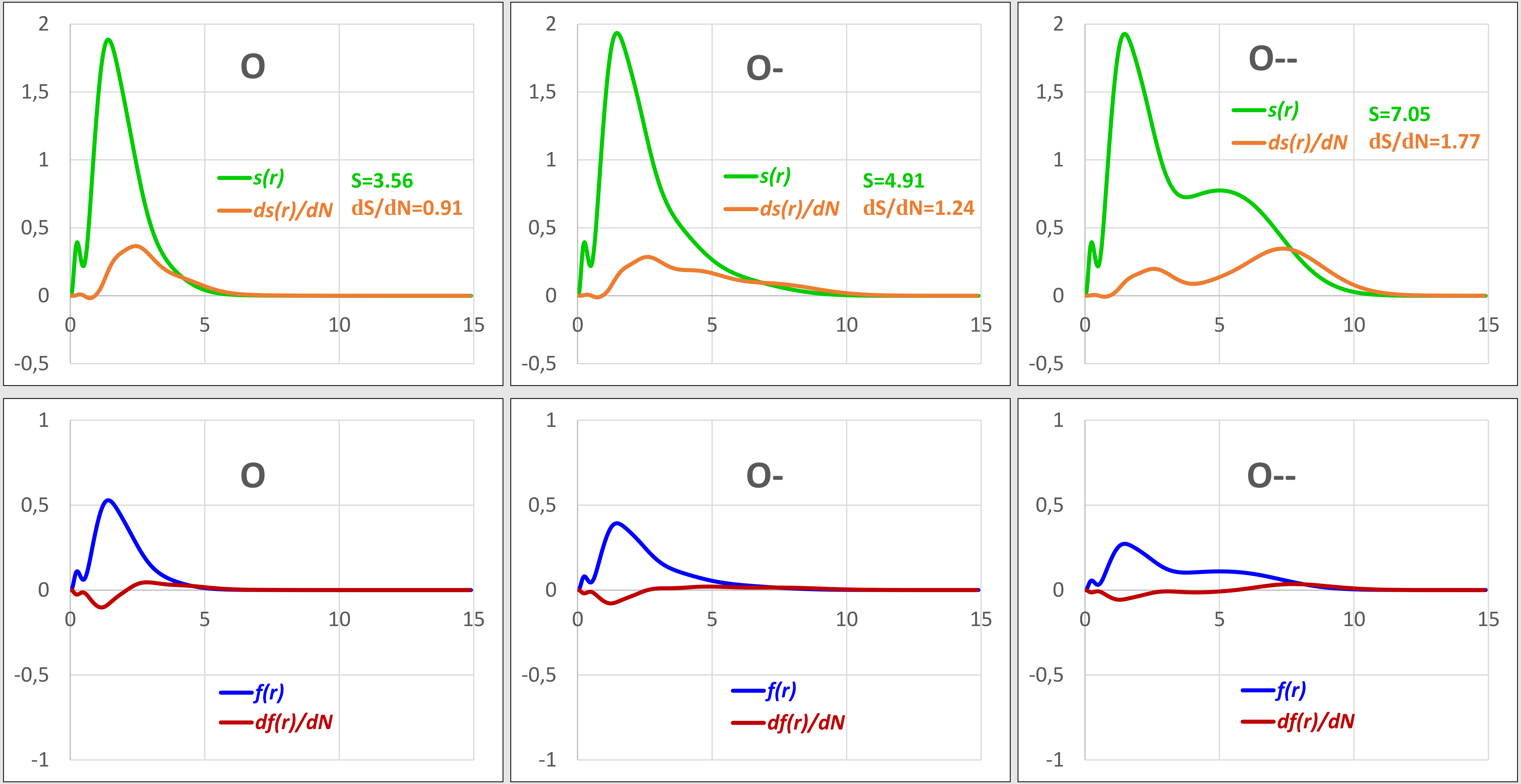

J. Zaklika, P. Ordon , L. Komorowski, Hyperhardness and hypersoftness of atoms and their ions,

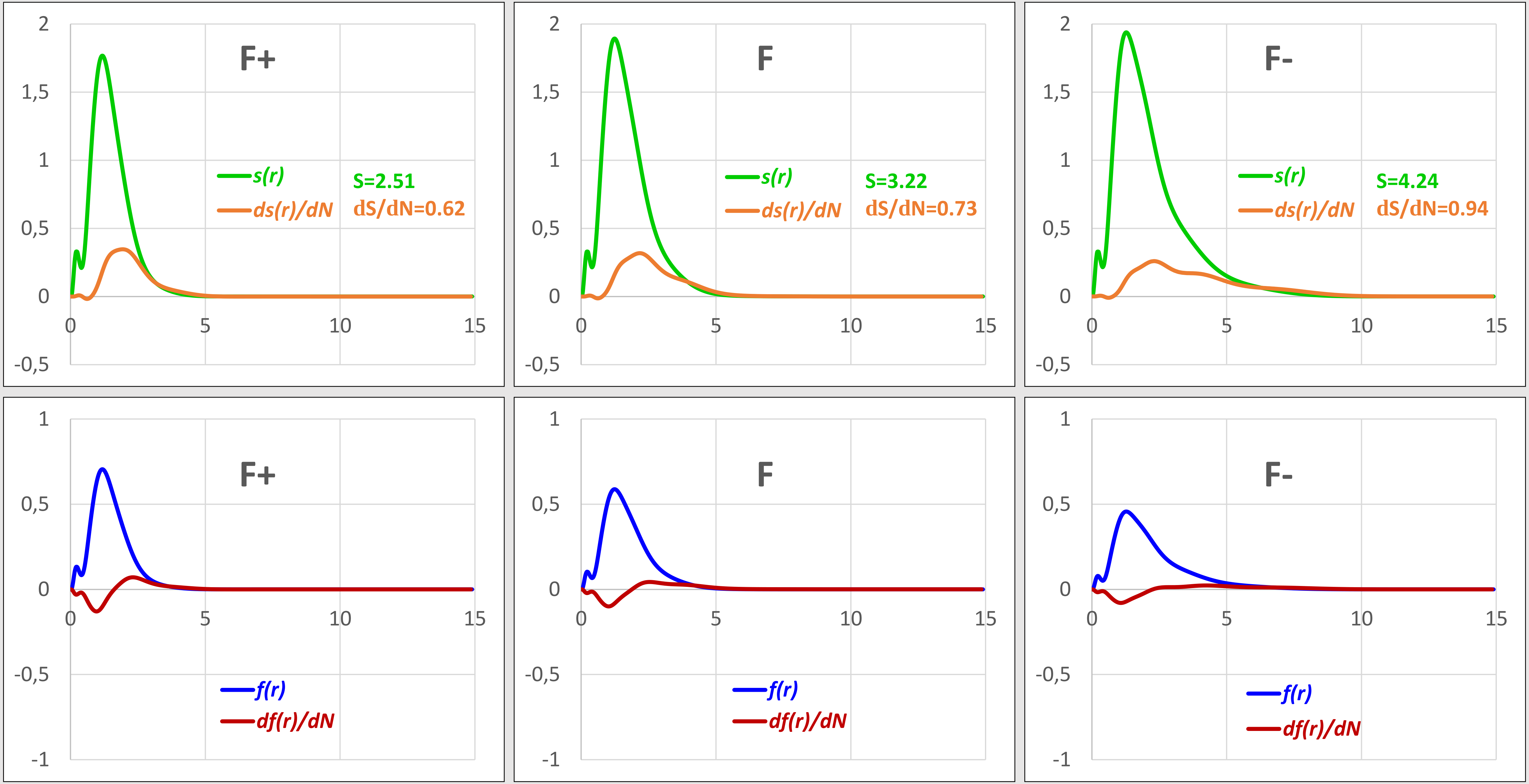

J. Zaklika, P. Ordon , L. Komorowski, Hyperhardness and hypersoftness of atoms and their ions,

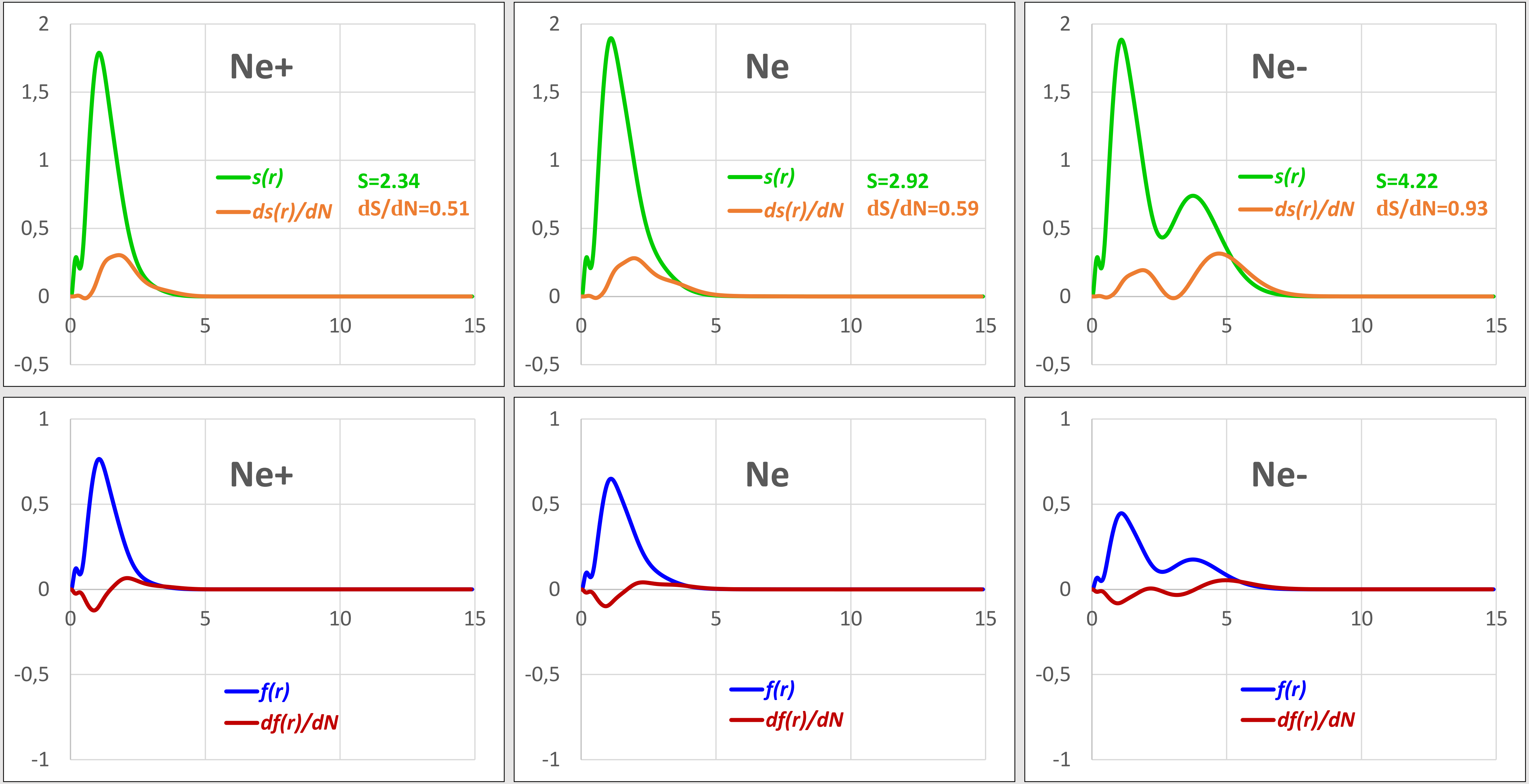

J. Zaklika, P. Ordon , L. Komorowski, Hyperhardness and hypersoftness of atoms and their ions,

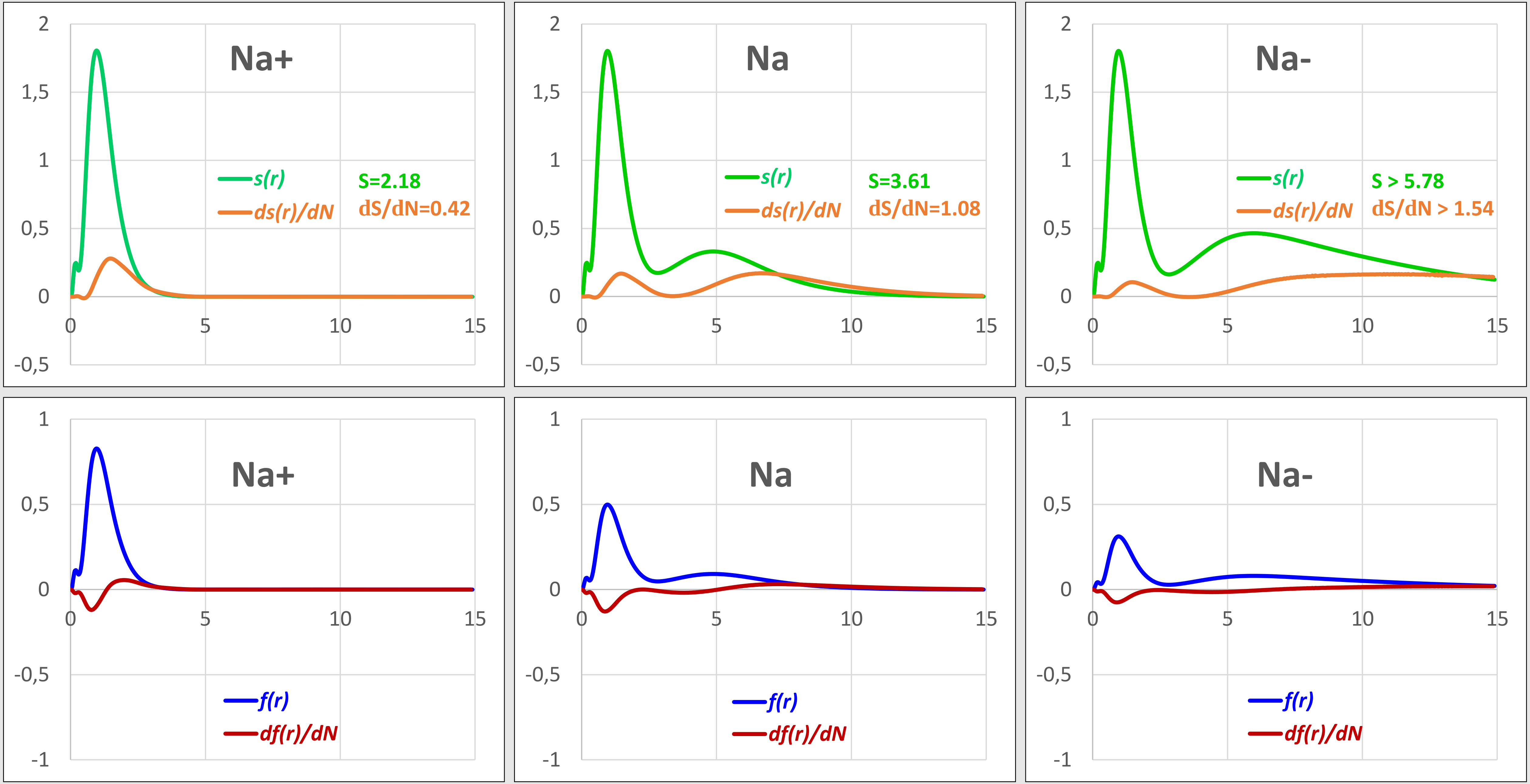

J. Zaklika, P. Ordon , L. Komorowski, Hyperhardness and hypersoftness of atoms and their ions,

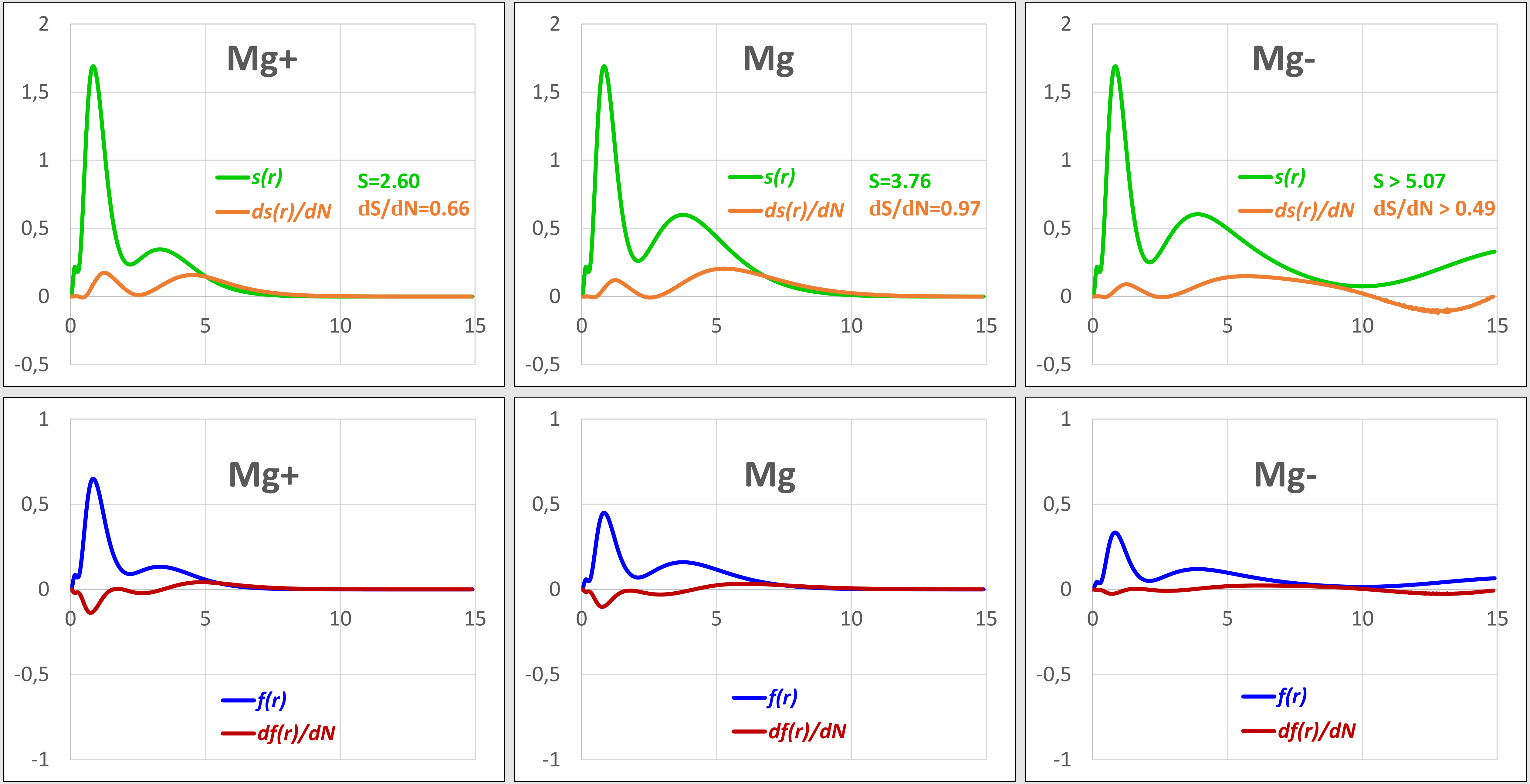

J. Zaklika, P. Ordon , L. Komorowski, Hyperhardness and hypersoftness of atoms and their ions,

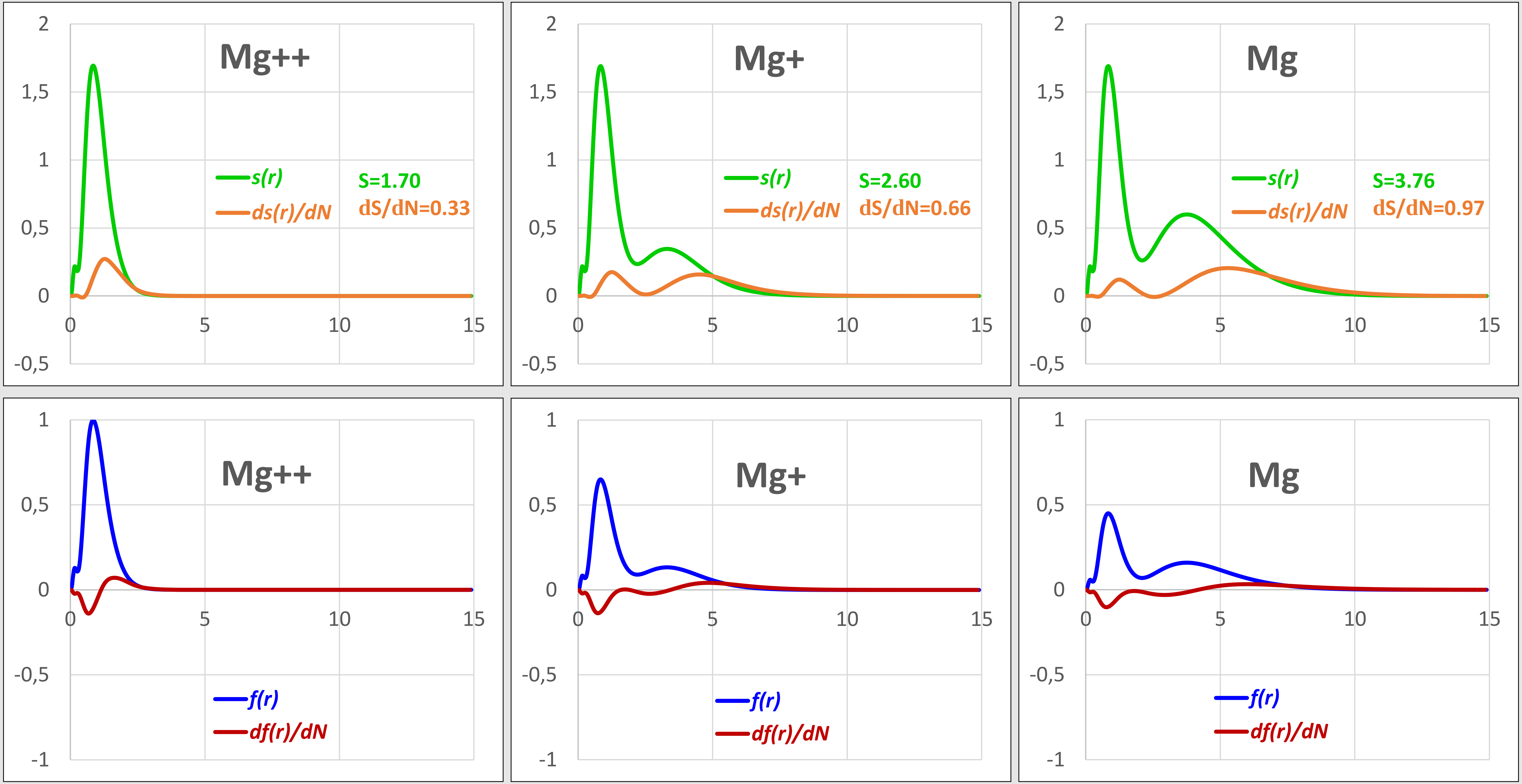

J. Zaklika, P. Ordon , L. Komorowski, Hyperhardness and hypersoftness of atoms and their ions,

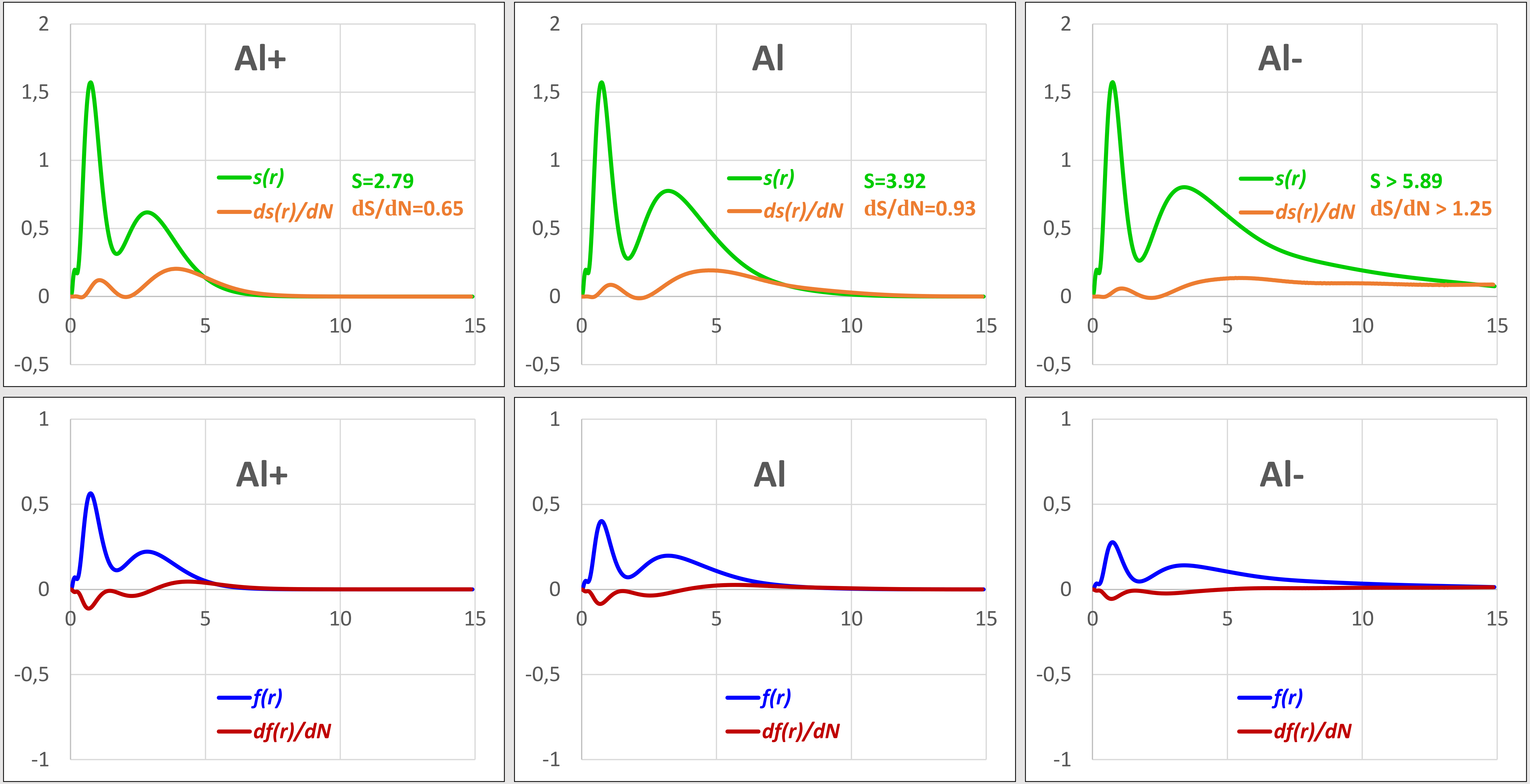

ELEMENTS

J. Zaklika, P. Ordon , L. Komorowski, Hyperhardness and hypersoftness of atoms and their ions,

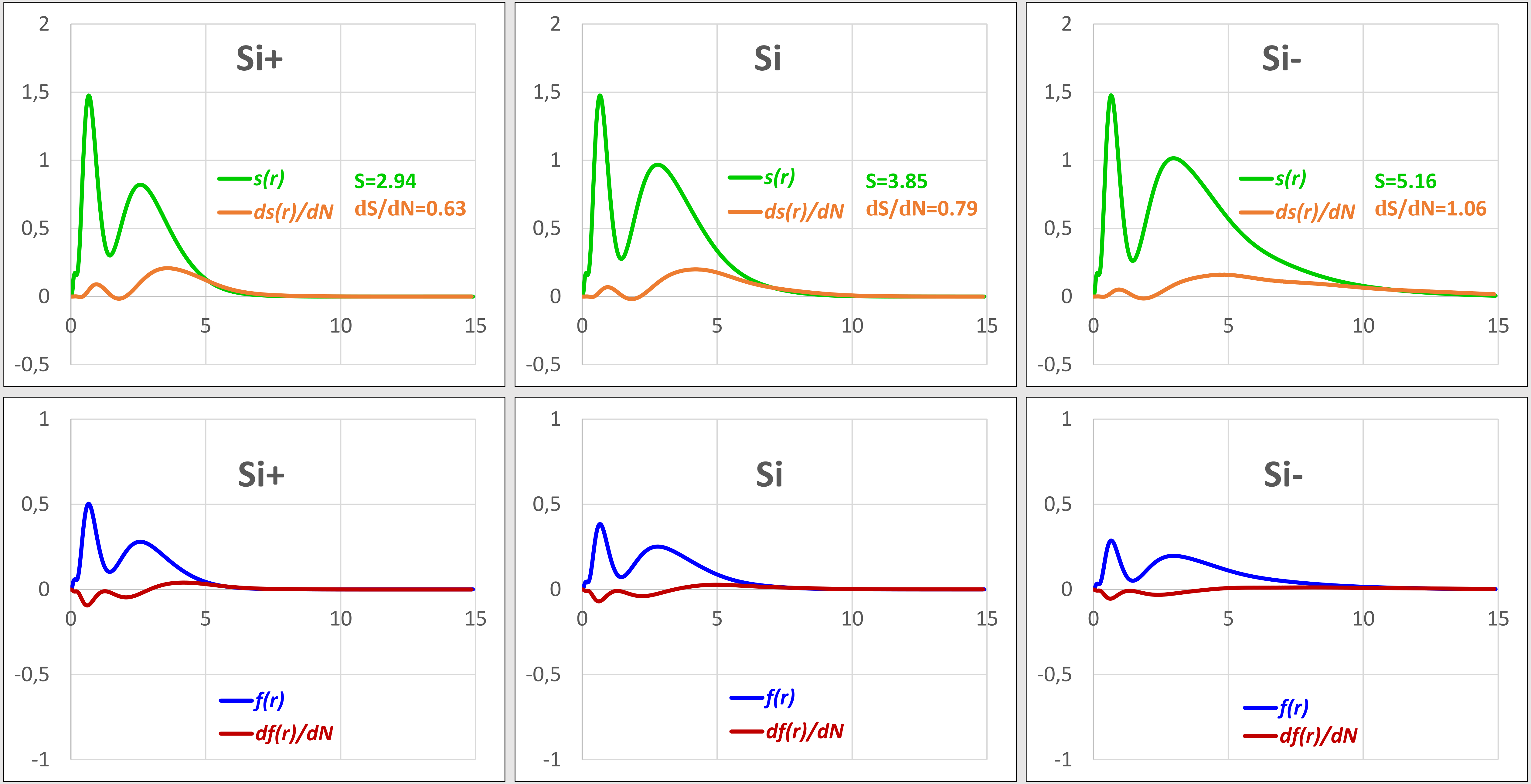
Si+
s(r)
ds(r)/dN
S=2.94
dS/dN=0.63
Si
s(r)
ds(r)/dN
S=3.85
dS/dN=0.79
Si-
s(r)
ds(r)/dN
S=5.16
dS/dN=1.06
2
1,5
1
0,5
0
-0,5
0
5
10
15
Si+
f(r)
df(r)/dN
Si
f(r)
df(r)/dN
Si-
f(r)
df(r)/dN
1
0,5
0
-0,5
-1

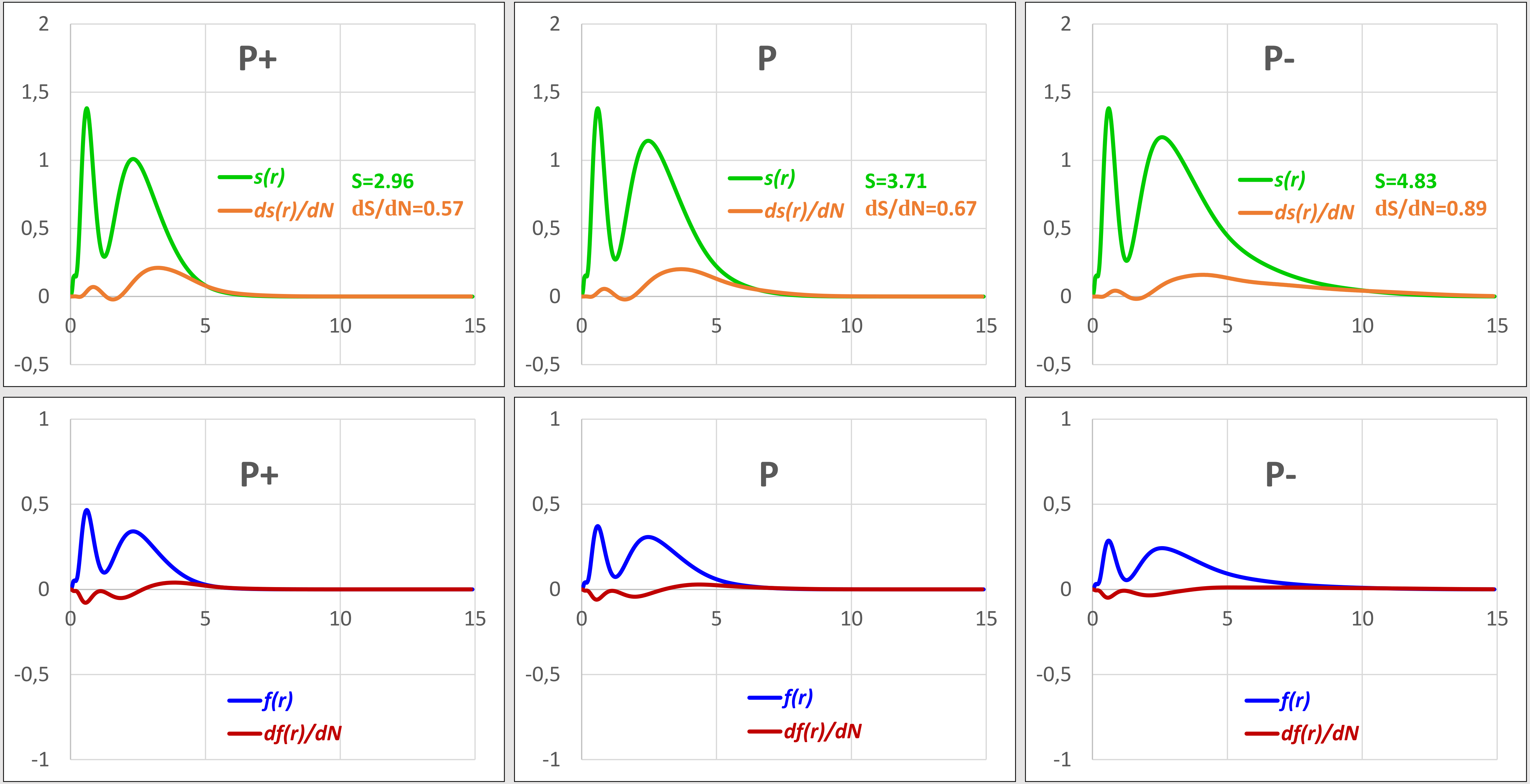

J. Zaklika, P. Ordon , L. Komorowski, Hyperhardness and hypersoftness of atoms and their ions,

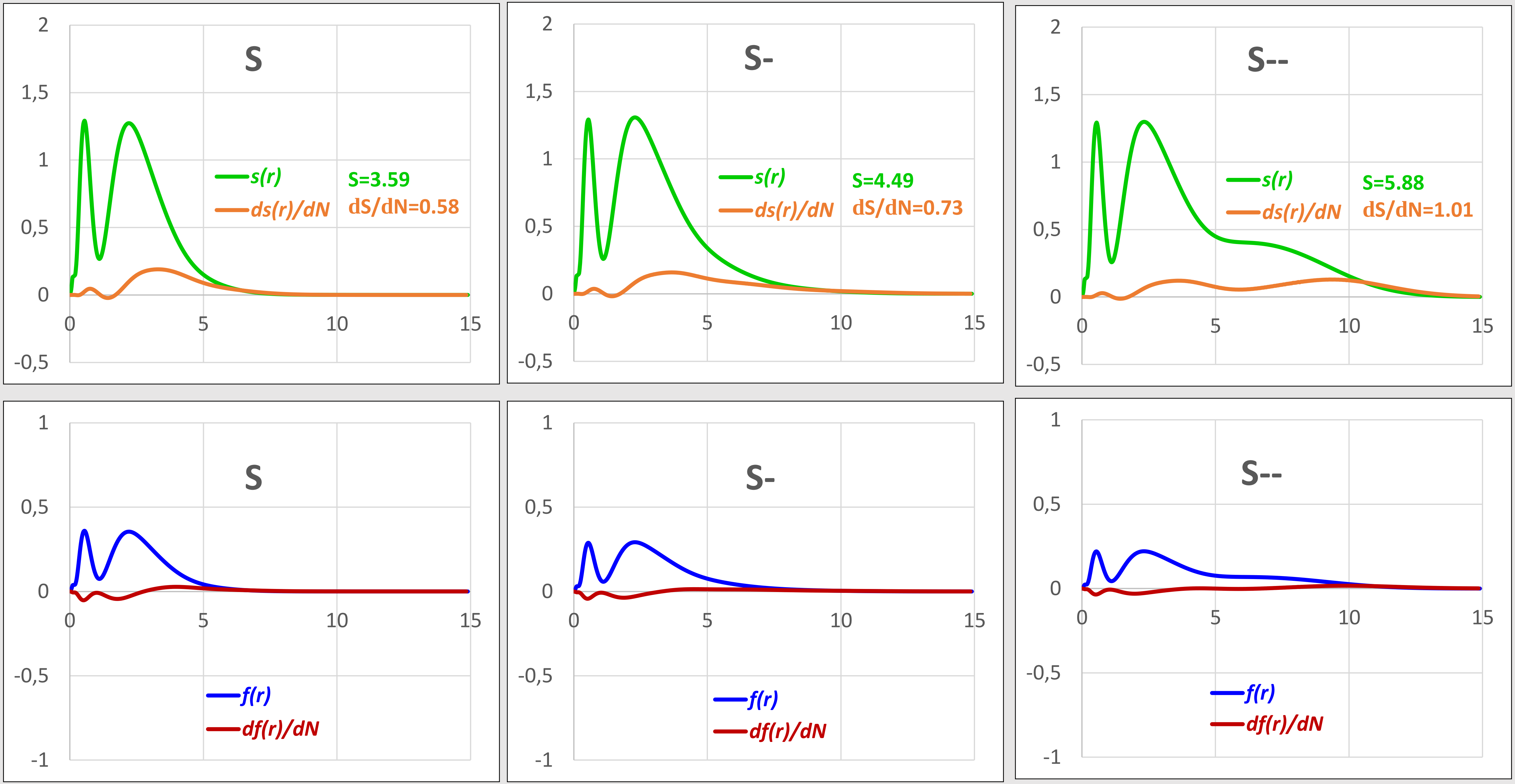

J. Zaklika, P. Ordon , L. Komorowski, Hyperhardness and hypersoftness of atoms and their ions,

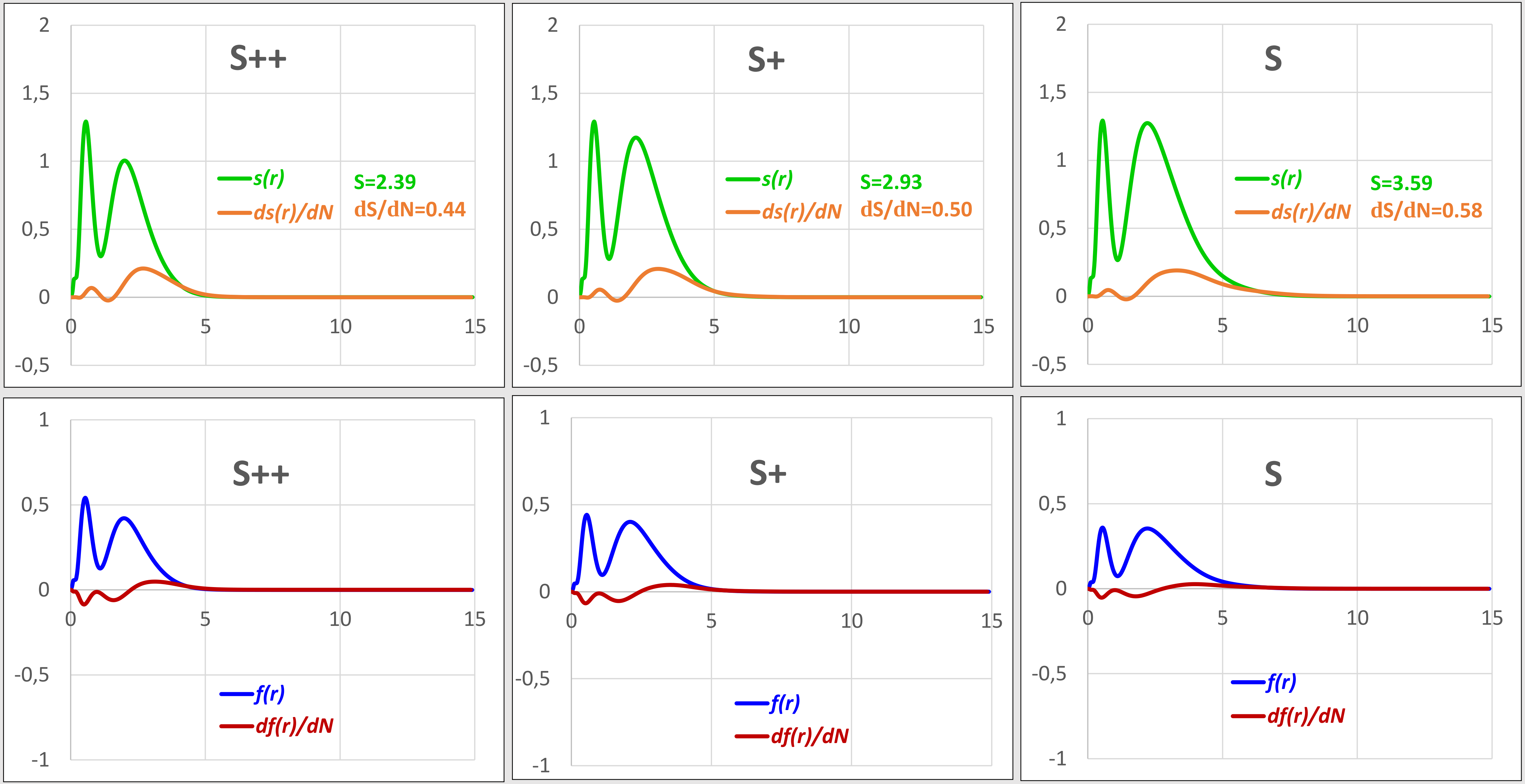
S++
s(r)
ds(r)/dN
S=2.39
dS/dN=0.44
S+
s(r)
ds(r)/dN
S=2.93
dS/dN=0.50
S
s(r)
ds(r)/dN
S=3.59
dS/dN=0.58
S++
f(r)
df(r)/dN
S+
f(r)
df(r)/dN
S
f(r)
df(r)/dN

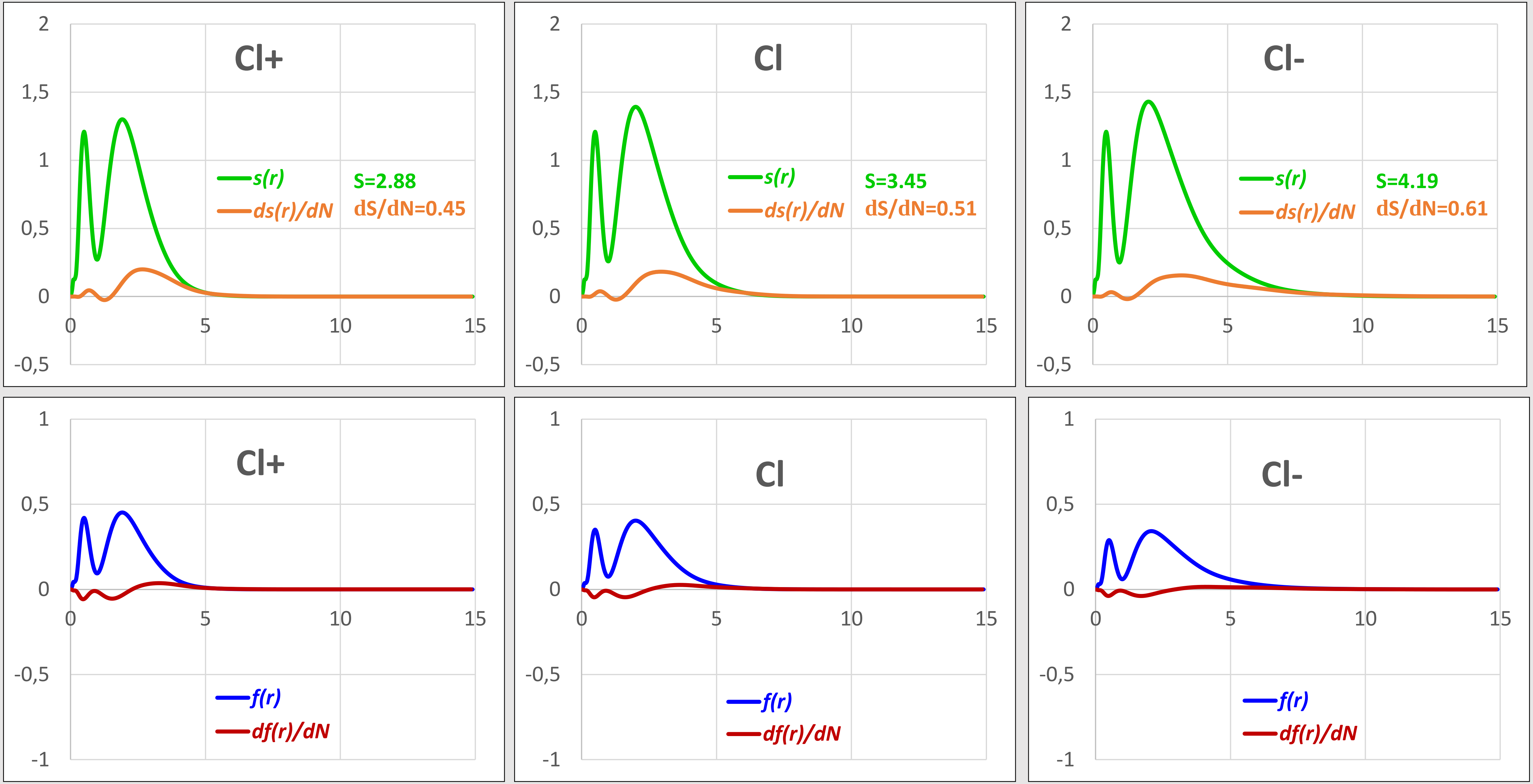

J. Zaklika, P. Ordon , L. Komorowski, Hyperhardness and hypersoftness of atoms and their ions,

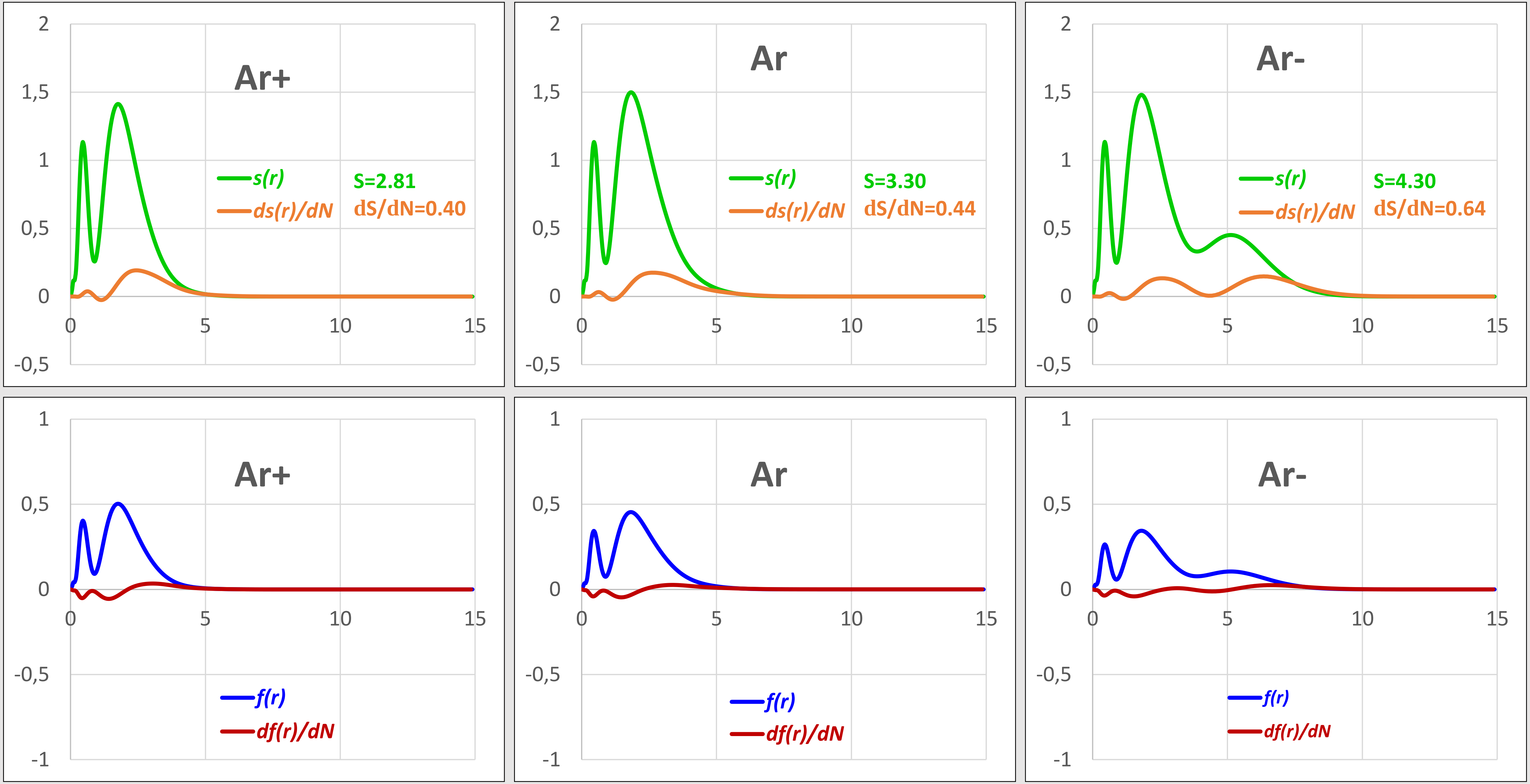

J. Zaklika, P. Ordon , L. Komorowski, Hyperhardness and hypersoftness of atoms and their ions,

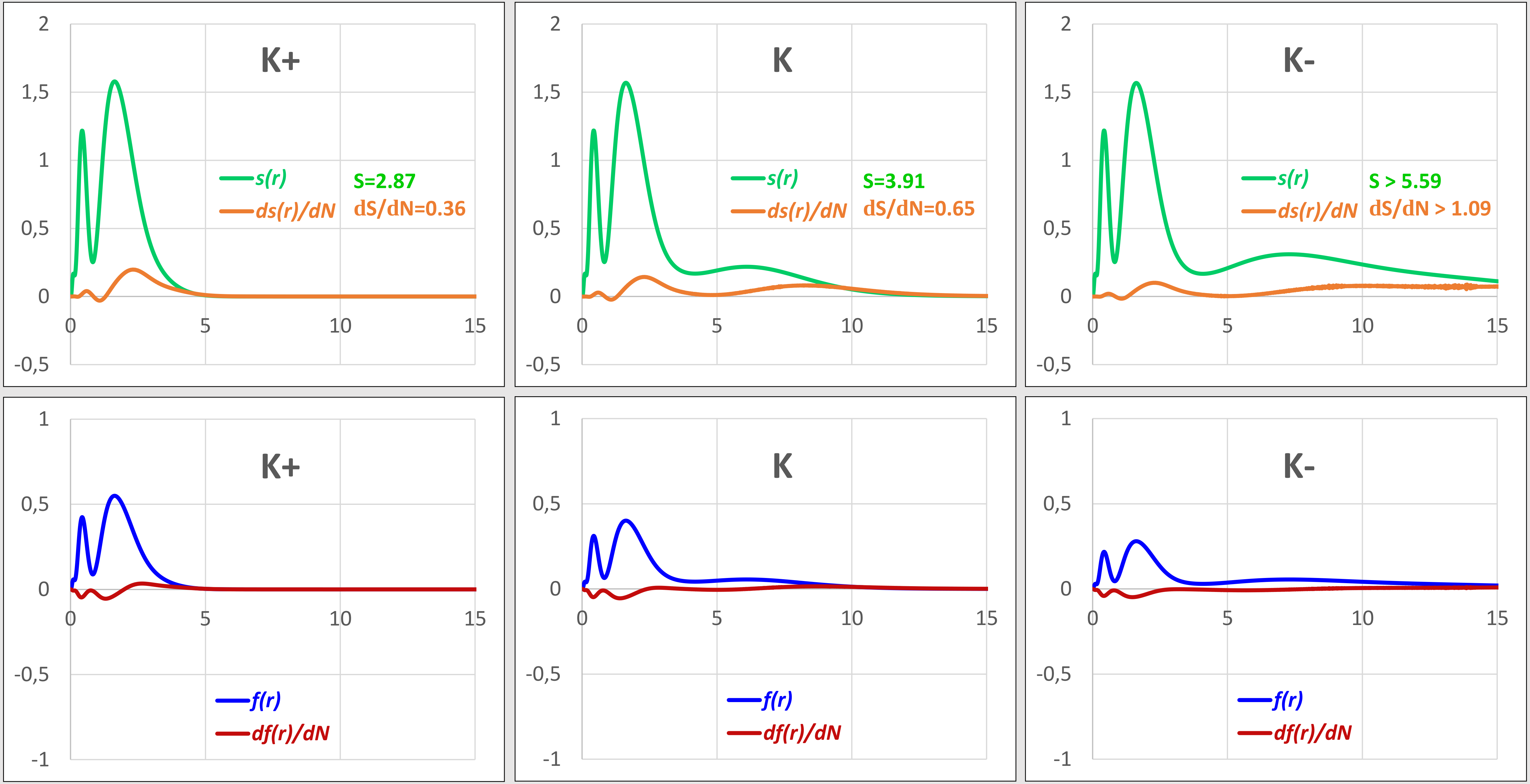

J. Zaklika, P. Ordon , L. Komorowski, Hyperhardness and hypersoftness of atoms and their ions,

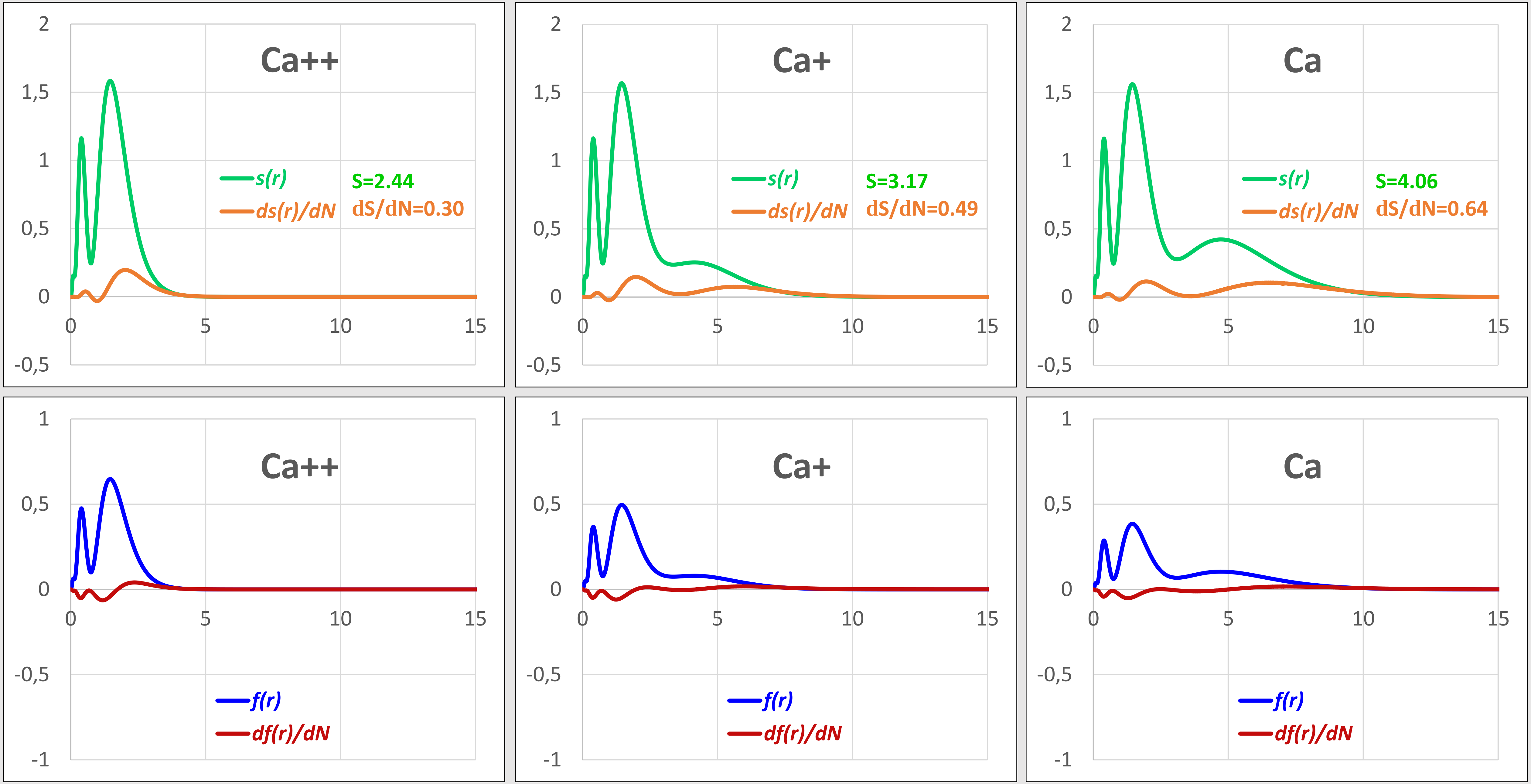

J. Zaklika, P. Ordon , L. Komorowski, Hyperhardness and hypersoftness of atoms and their ions,

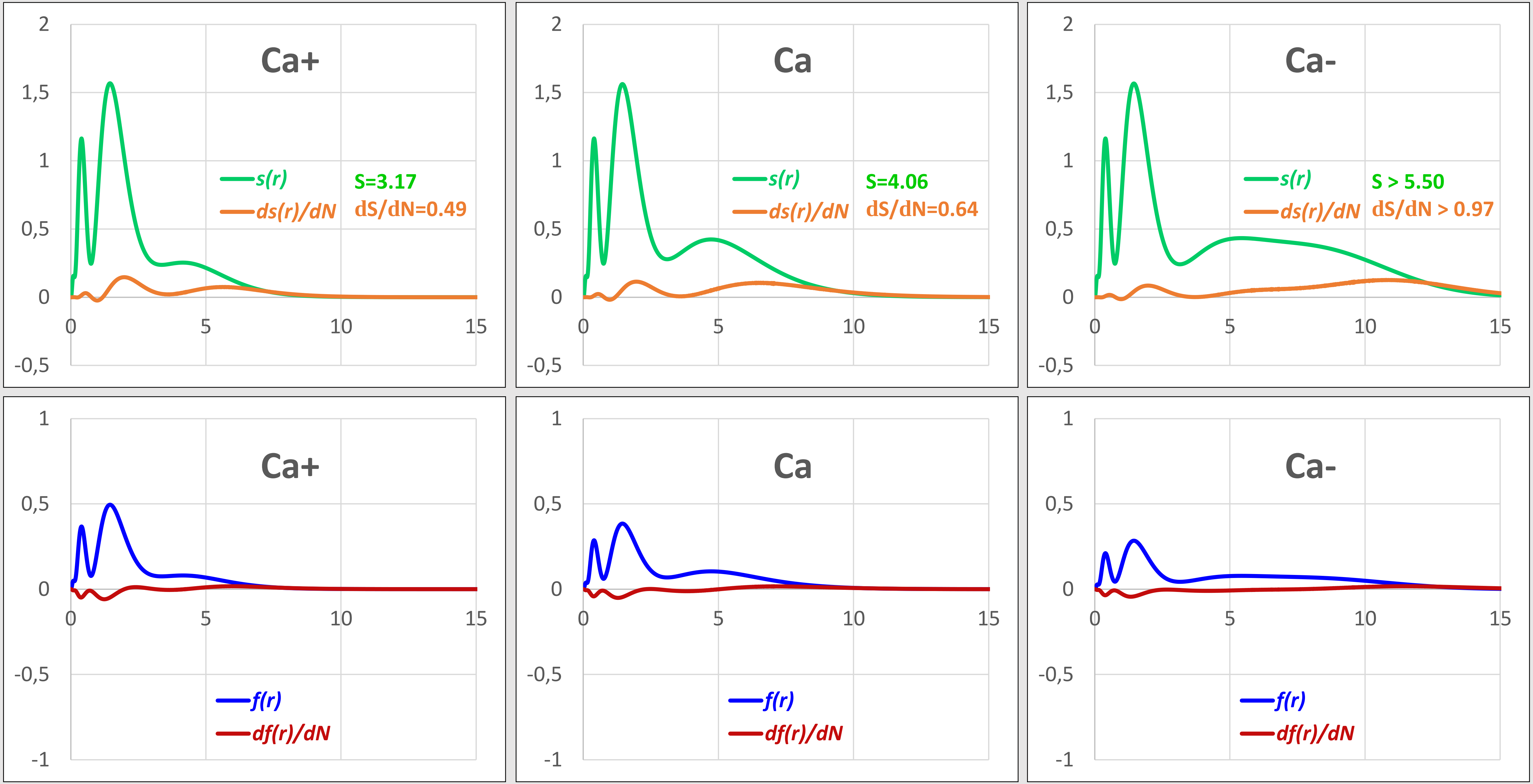

ELEMENTS

J. Zaklika, P. Ordon , L. Komorowski, Hyperhardness and hypersoftness of atoms and their ions,

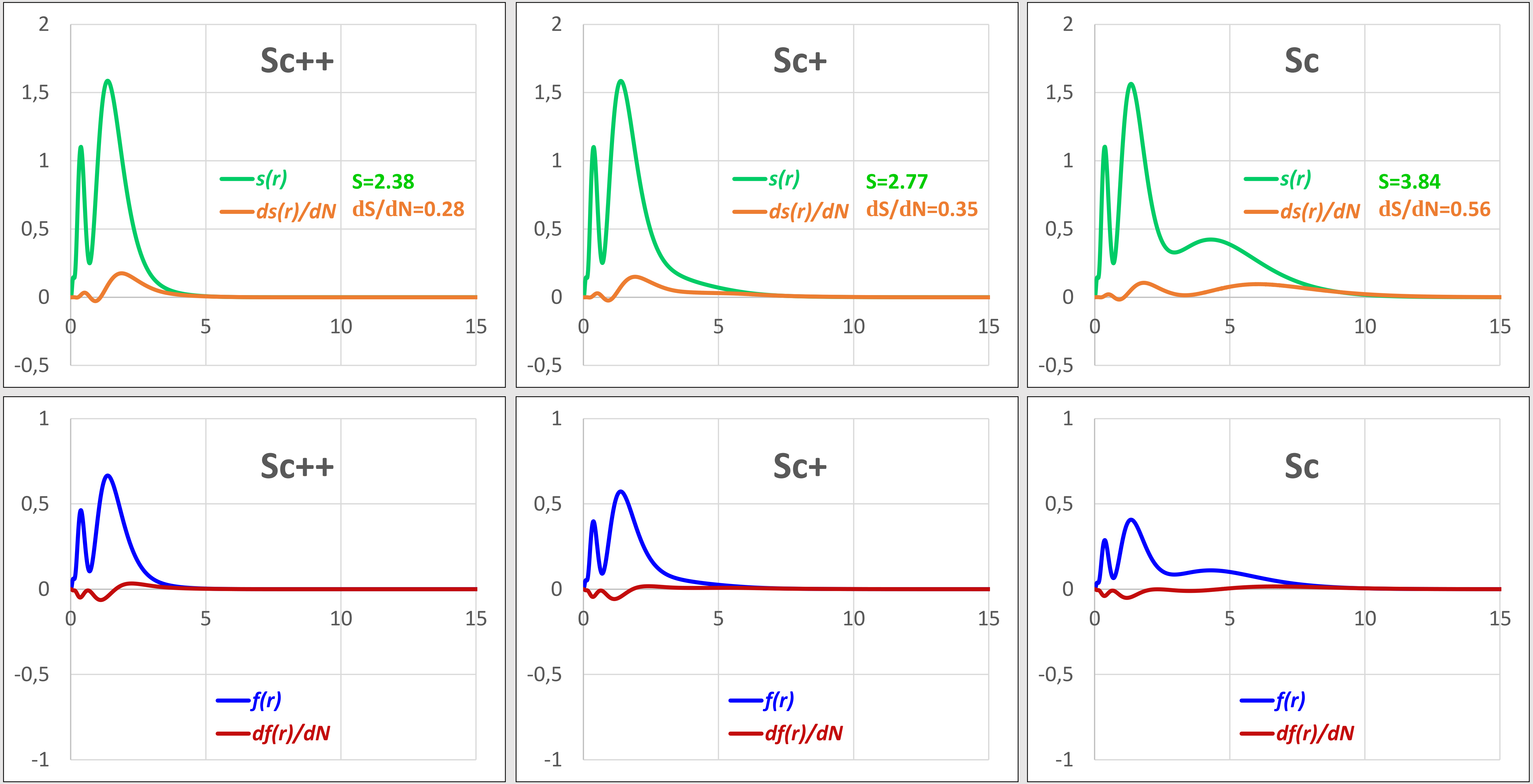

Sc++
s(r)
ds(r)/dN
S=2.38
dS/dN=0.28
Sc+
s(r)
ds(r)/dN
S=2.77
dS/dN=0.35
Sc
s(r)
ds(r)/dN
S=3.84
dS/dN=0.56
Sc++
f(r)
df(r)/dN
Sc+
f(r)
df(r)/dN
Sc
f(r)
df(r)/dN

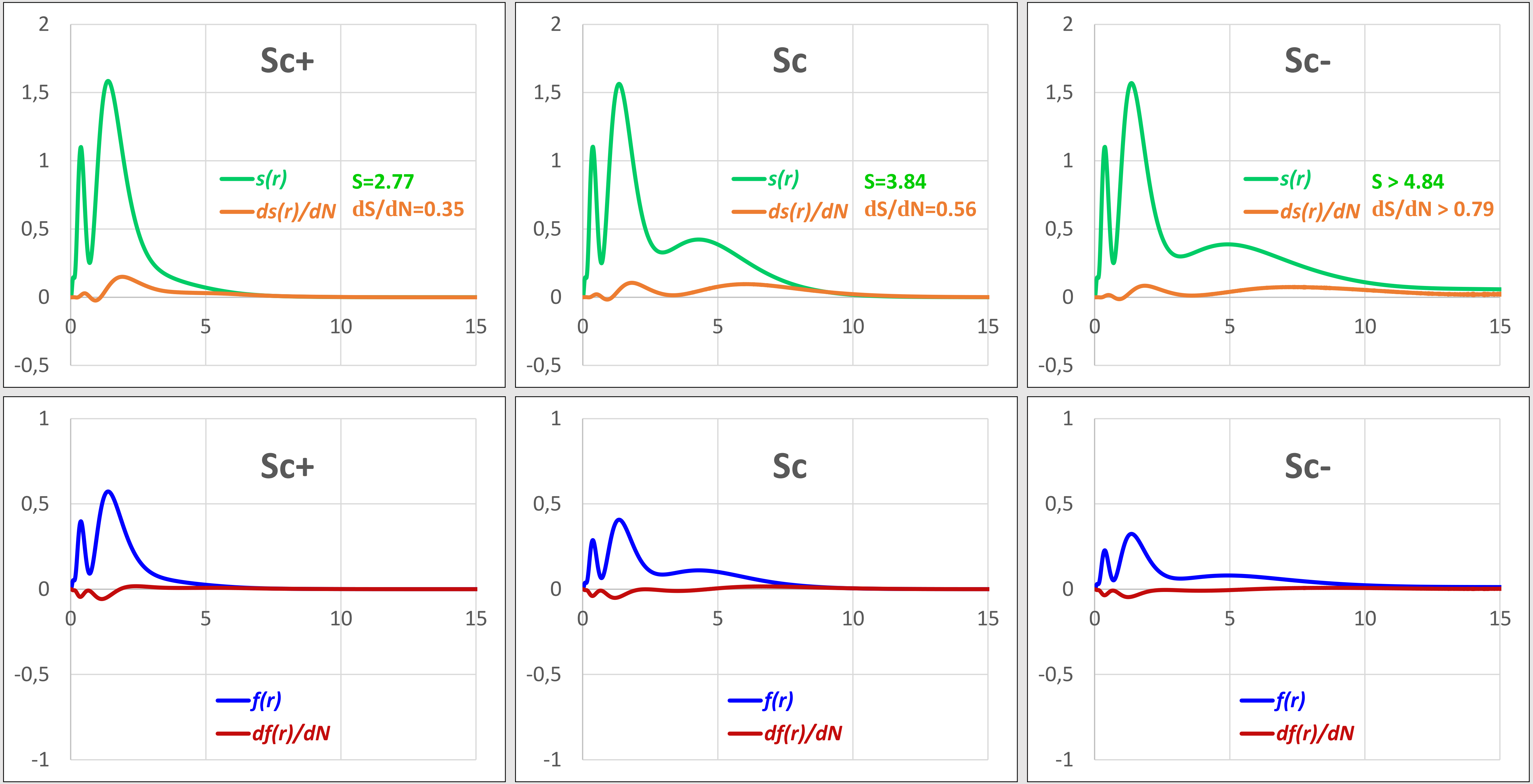

J. Zaklika, P. Ordon , L. Komorowski, Hyperhardness and hypersoftness of atoms and their ions,

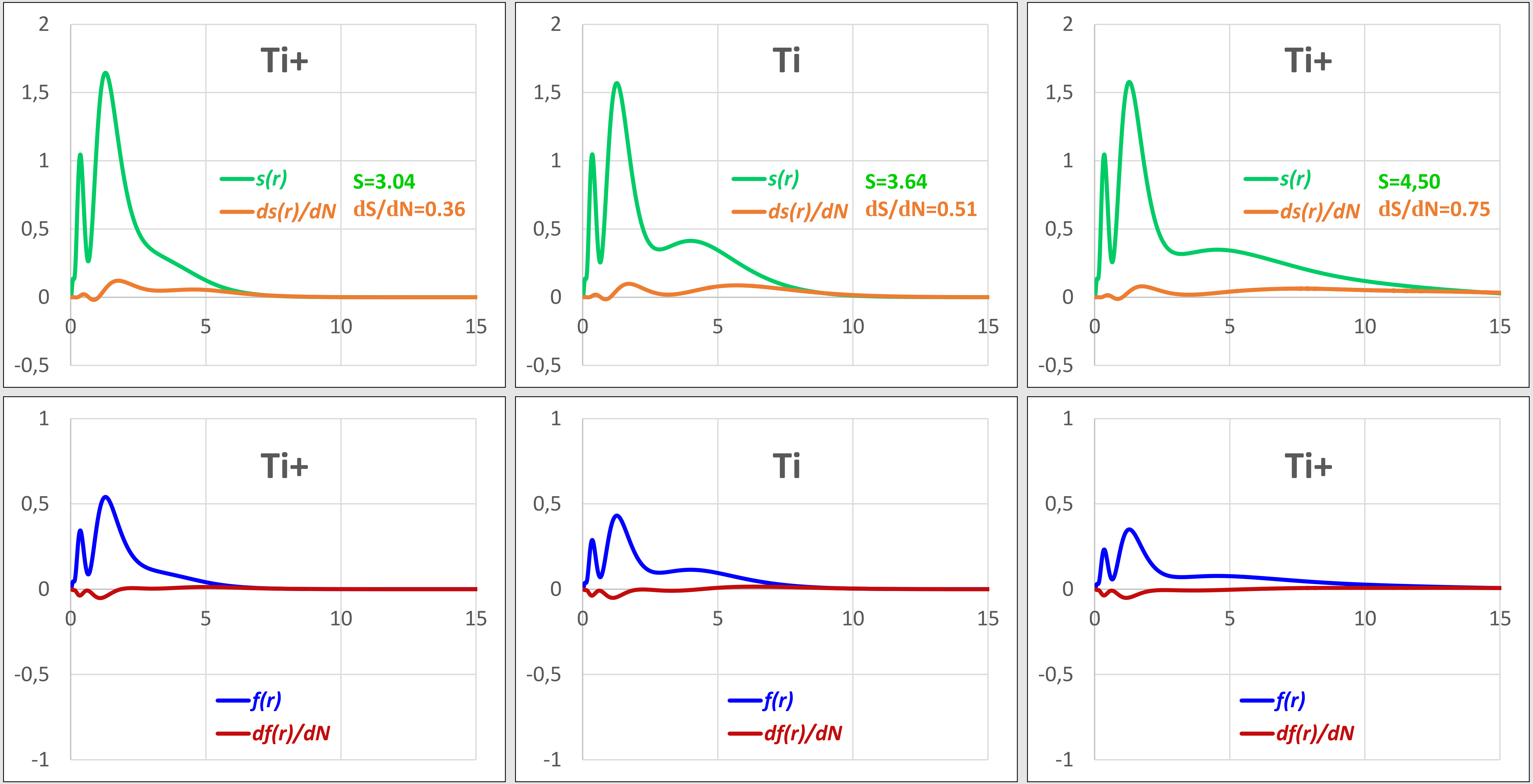

Ti+
s(r)
ds(r)/dN
S=3.04
dS/dN=0.36
Ti
s(r)
ds(r)/dN
S=3.64
dS/dN=0.51
Ti+
s(r)
ds(r)/dN
S=4,50
dS/dN=0.75
Ti+
f(r)
df(r)/dN
Ti
f(r)
df(r)/dN
Ti+
f(r)
df(r)/dN

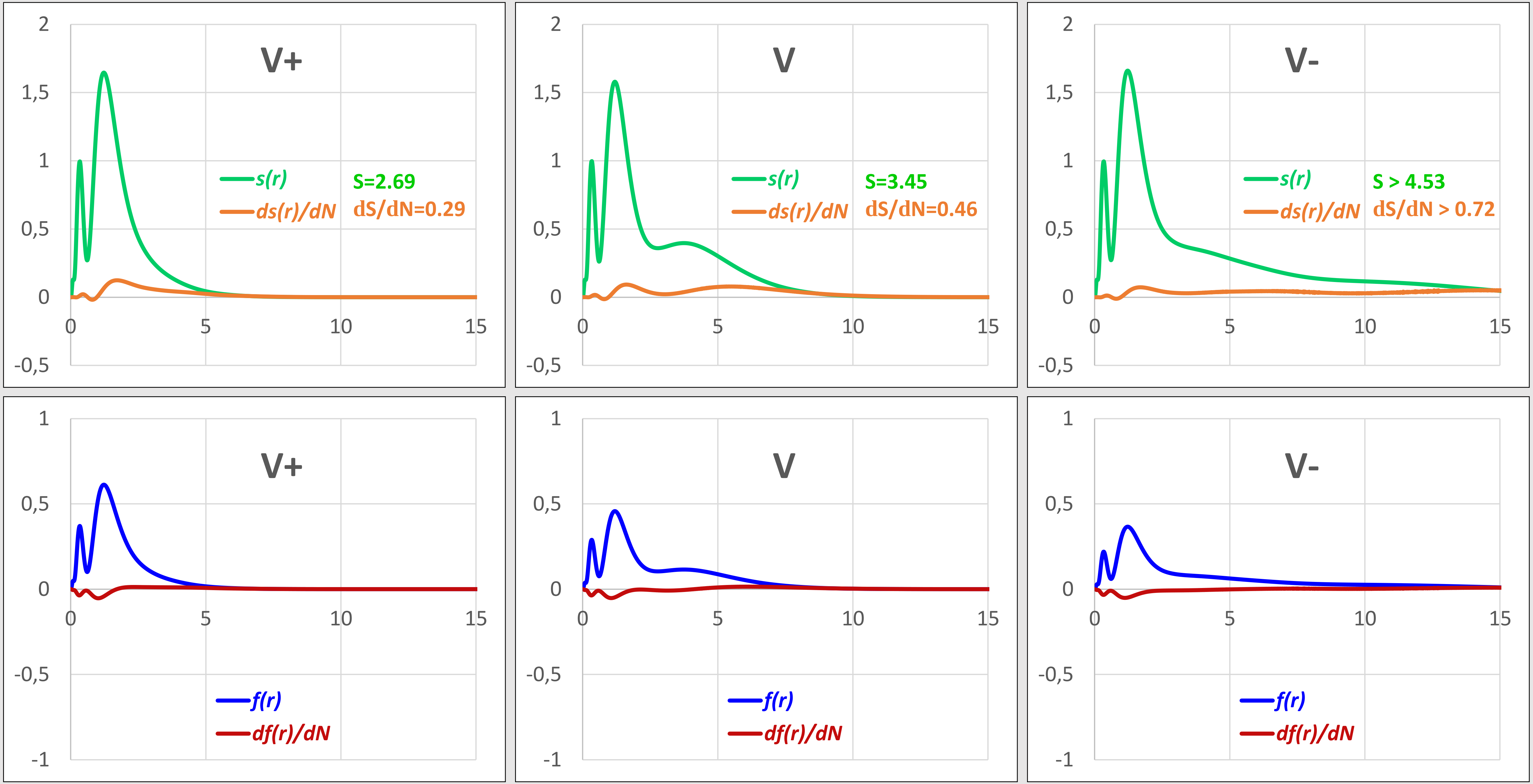

ELEMENTS

J. Zaklika, P. Ordon , L. Komorowski, Hyperhardness and hypersoftness of atoms and their ions,

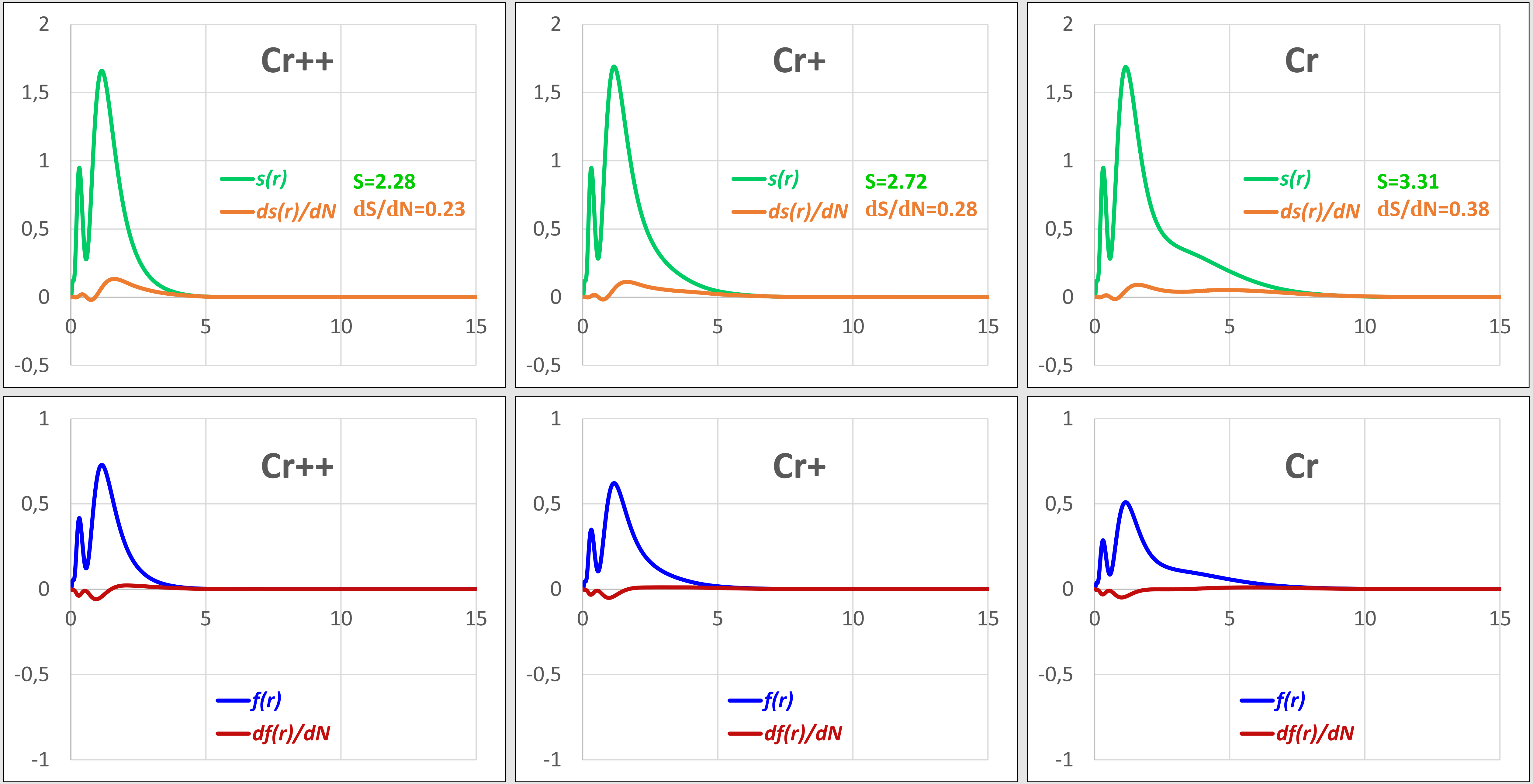
Cr++
s(r)
ds(r)/dN
S=2.28
dS/dN=0.23
Cr+
s(r)
ds(r)/dN
S=2.72
dS/dN=0.28
Cr
s(r)
ds(r)/dN
S=3.31
dS/dN=0.38
Cr++
f(r)
df(r)/dN
Cr+
f(r)
df(r)/dN
Cr
f(r)
df(r)/dN

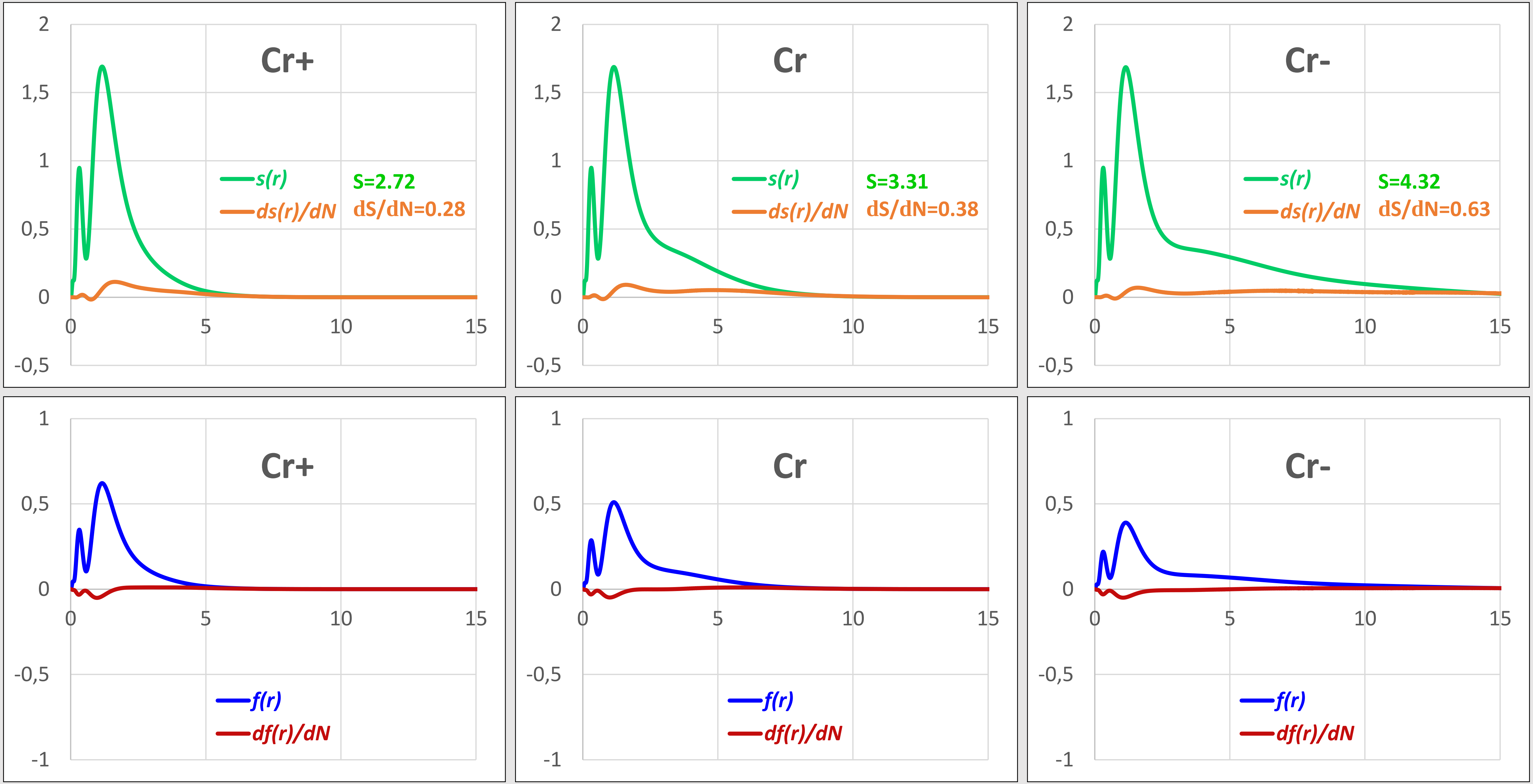

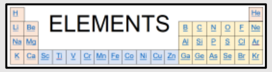

J. Zaklika, P. Ordon , L. Komorowski, Hyperhardness and hypersoftness of atoms and their ions,

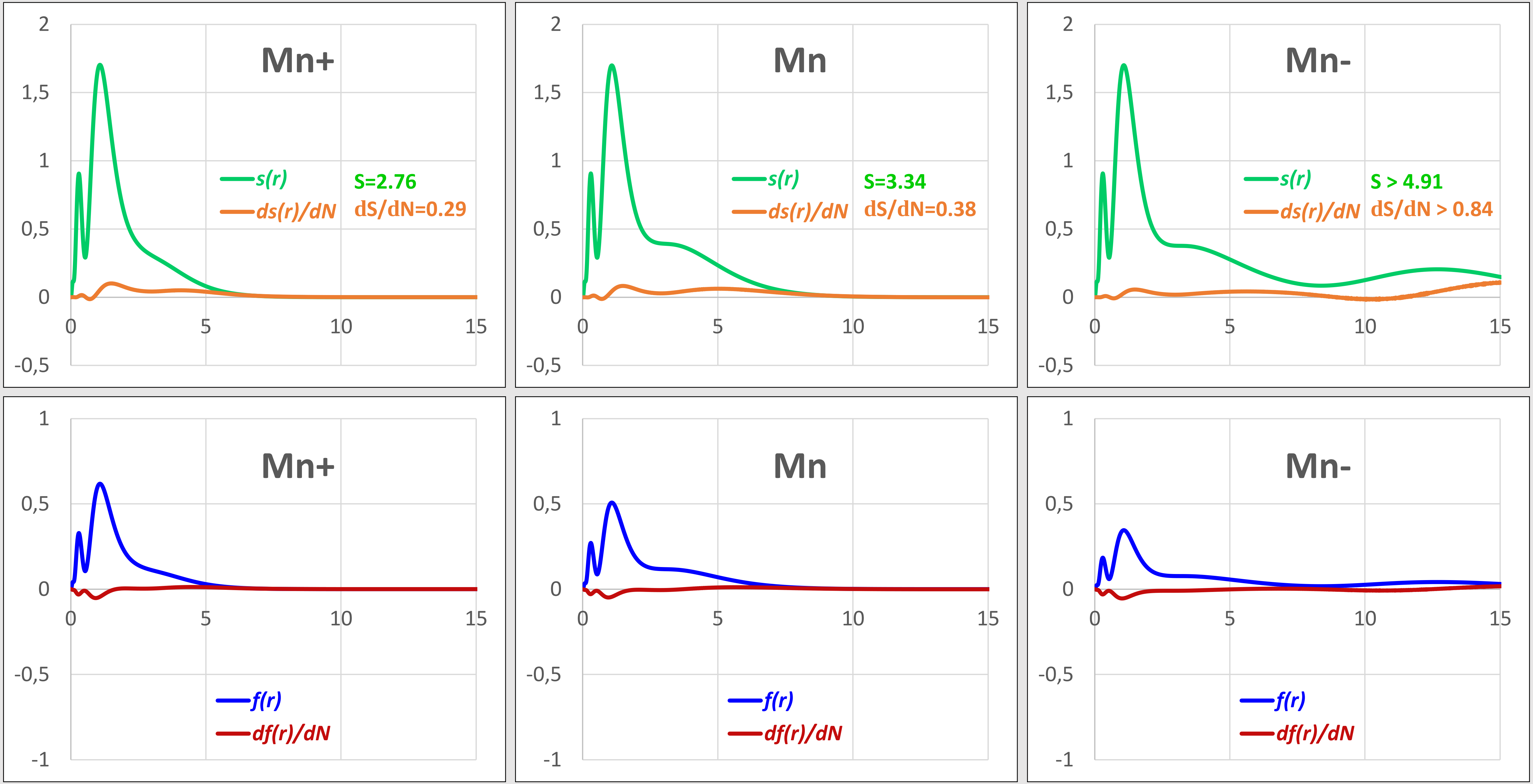

ELEMENTS

J. Zaklika, P. Ordon , L. Komorowski, Hyperhardness and hypersoftness of atoms and their ions,

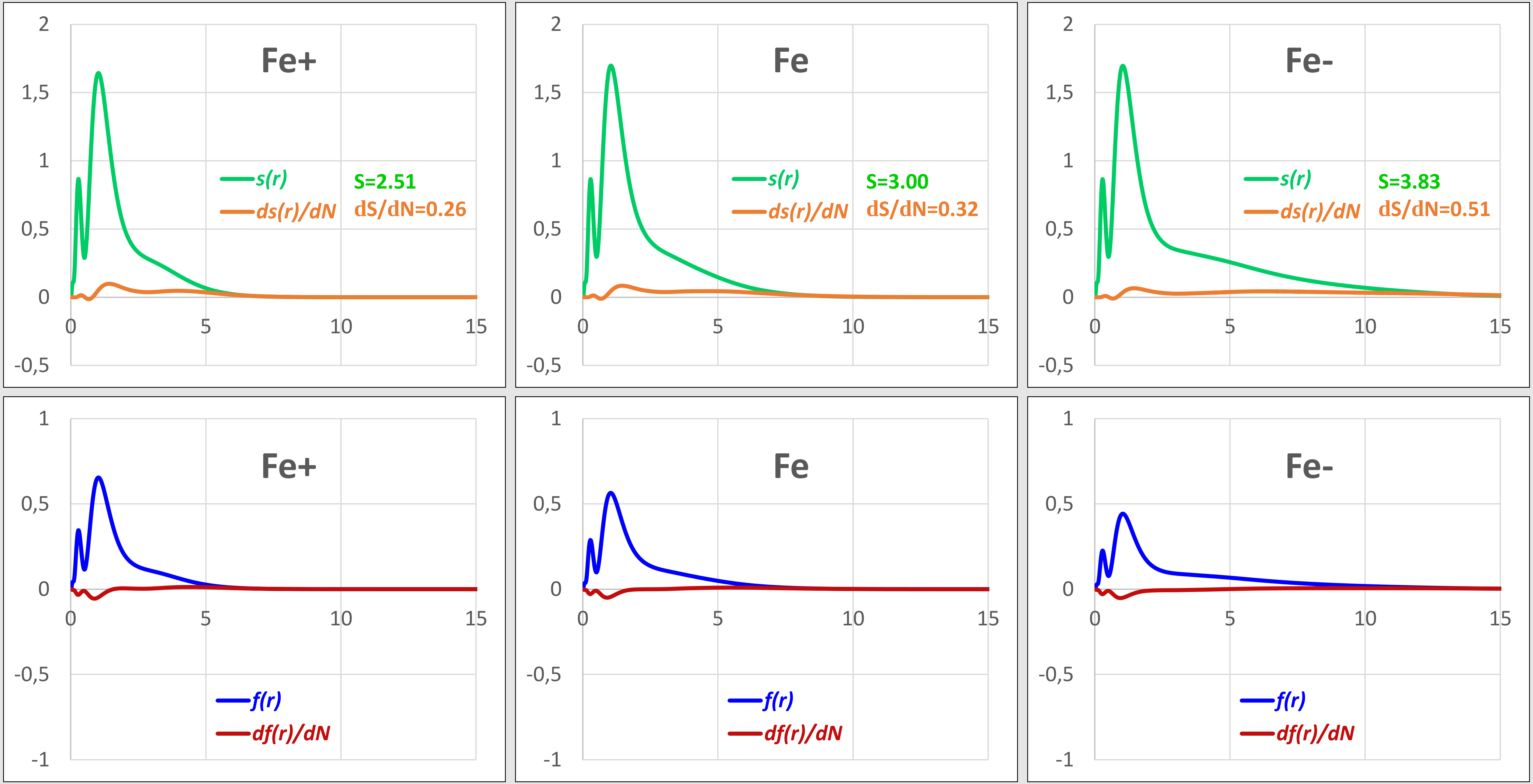

J. Zaklika, P. Ordon , L. Komorowski, Hyperhardness and hypersoftness of atoms and their ions,

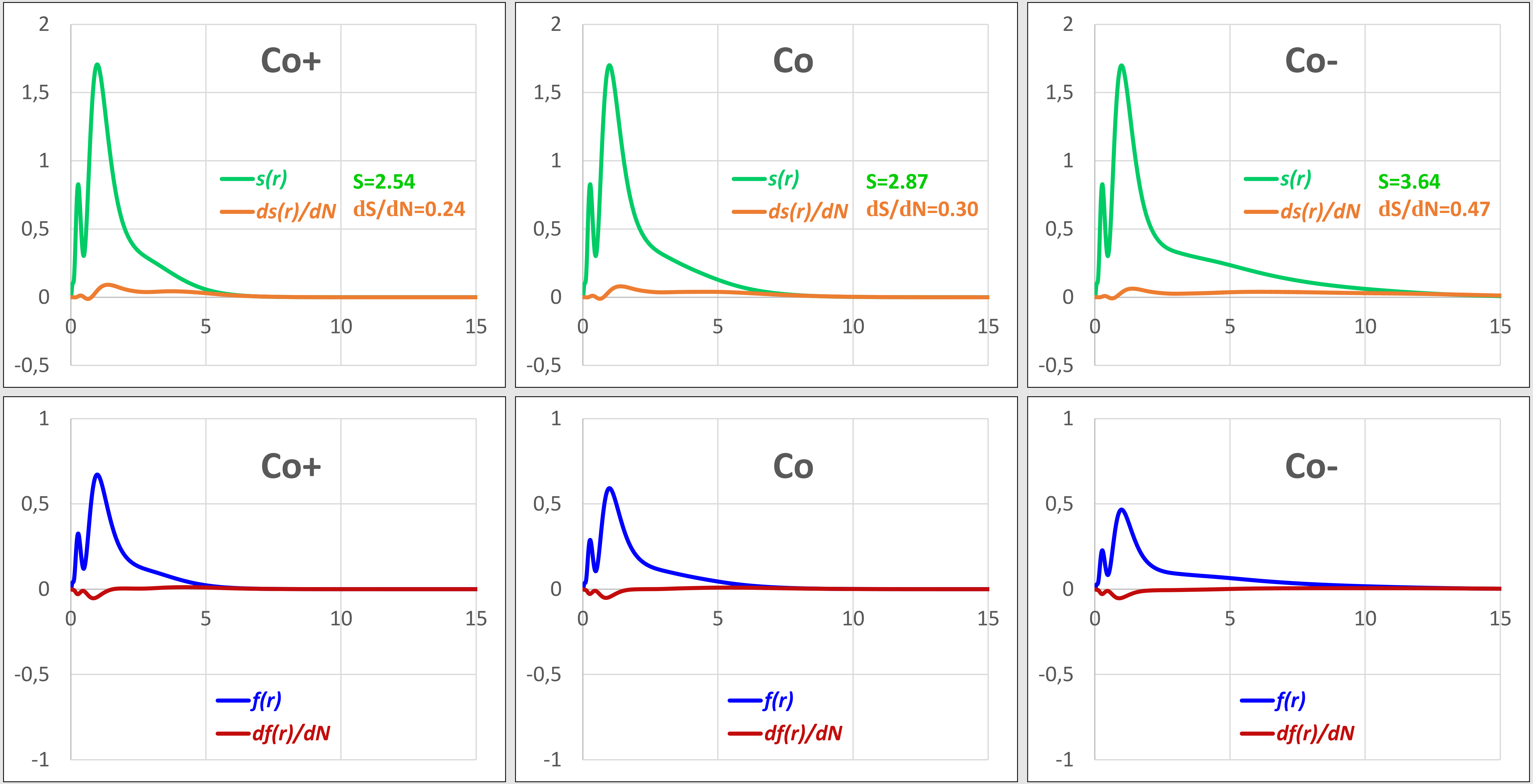

J. Zaklika, P. Ordon , L. Komorowski, Hyperhardness and hypersoftness of atoms and their ions,

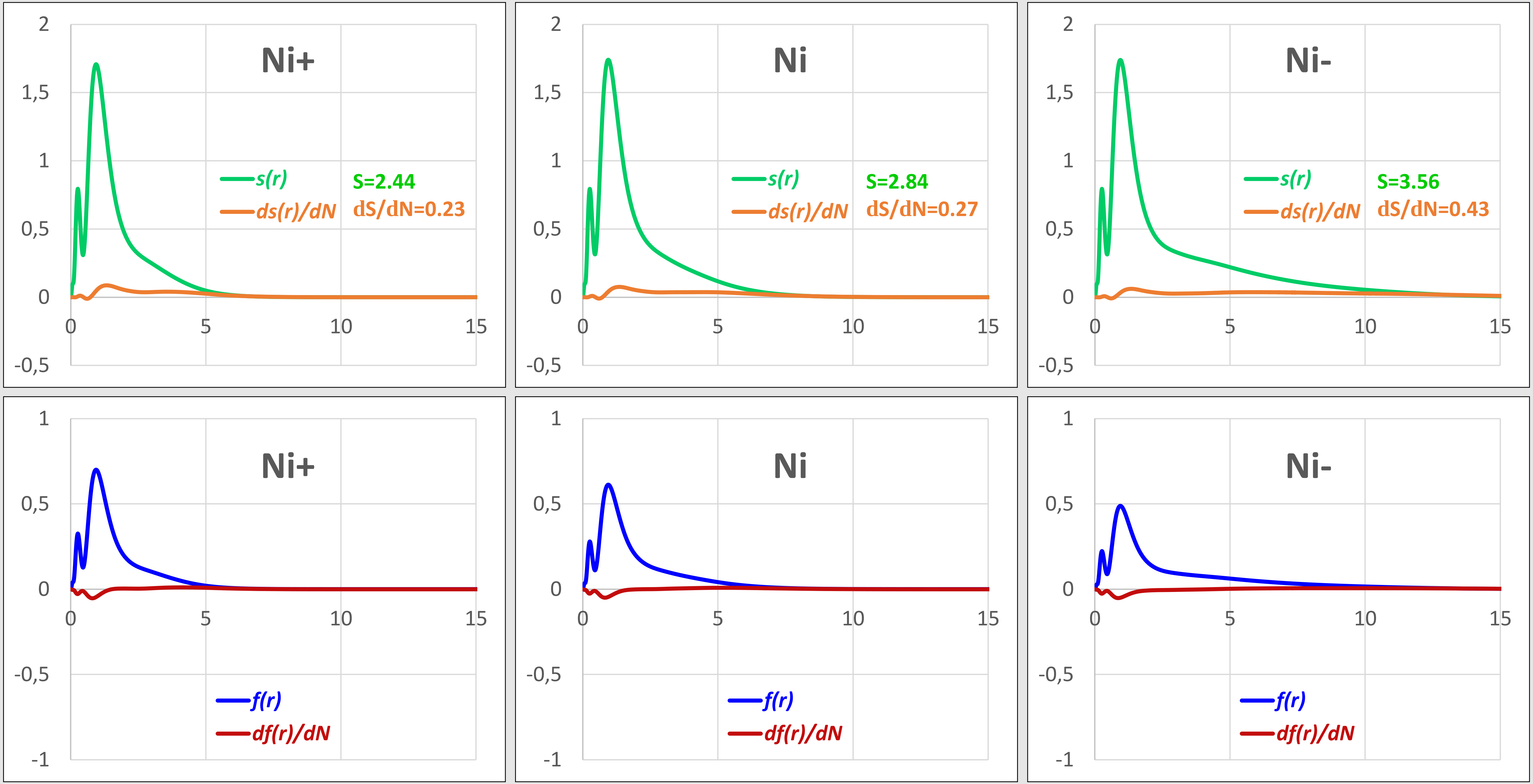

J. Zaklika, P. Ordon , L. Komorowski, Hyperhardness and hypersoftness of atoms and their ions,

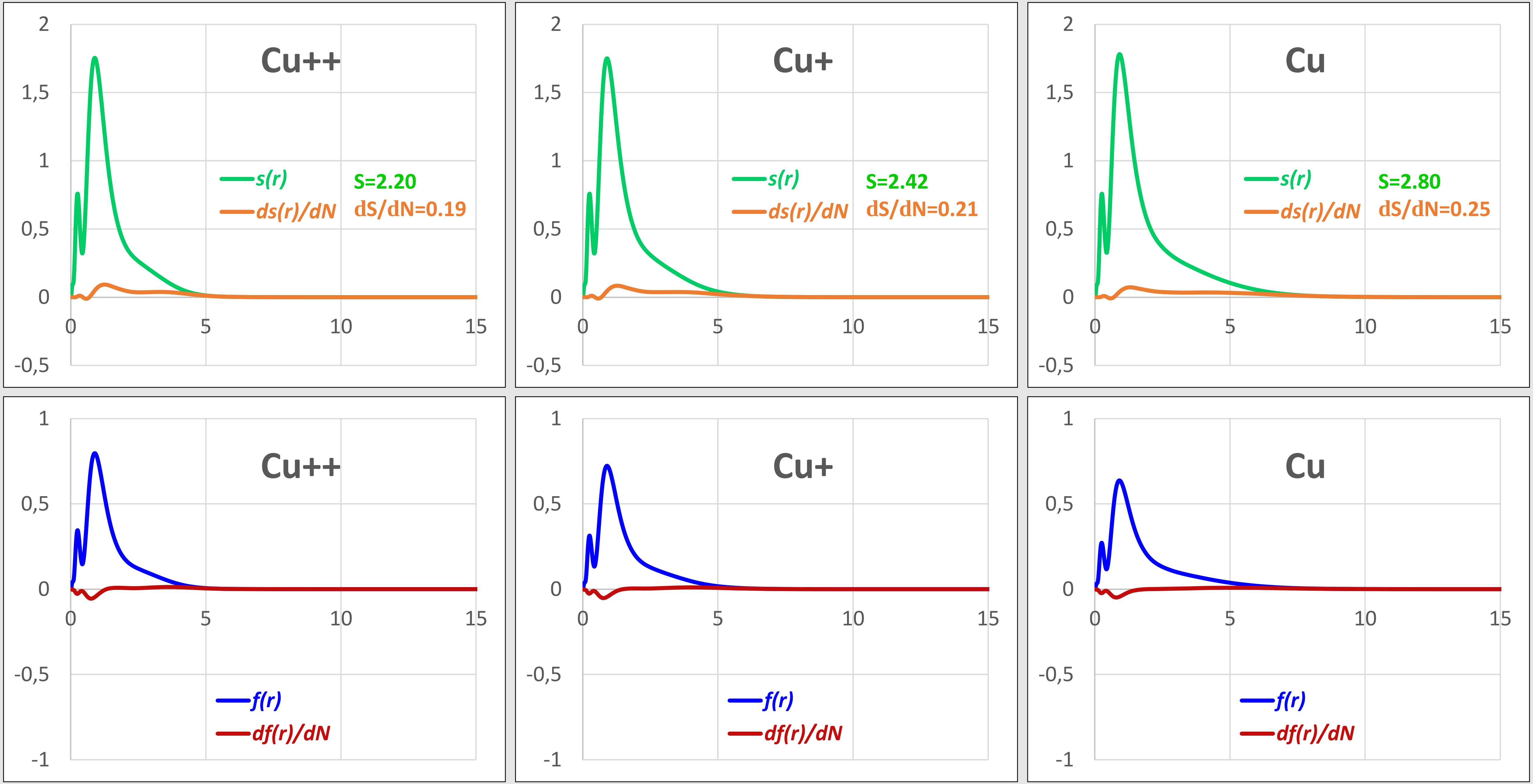

J. Zaklika, P. Ordon , L. Komorowski, Hyperhardness and hypersoftness of atoms and their ions,

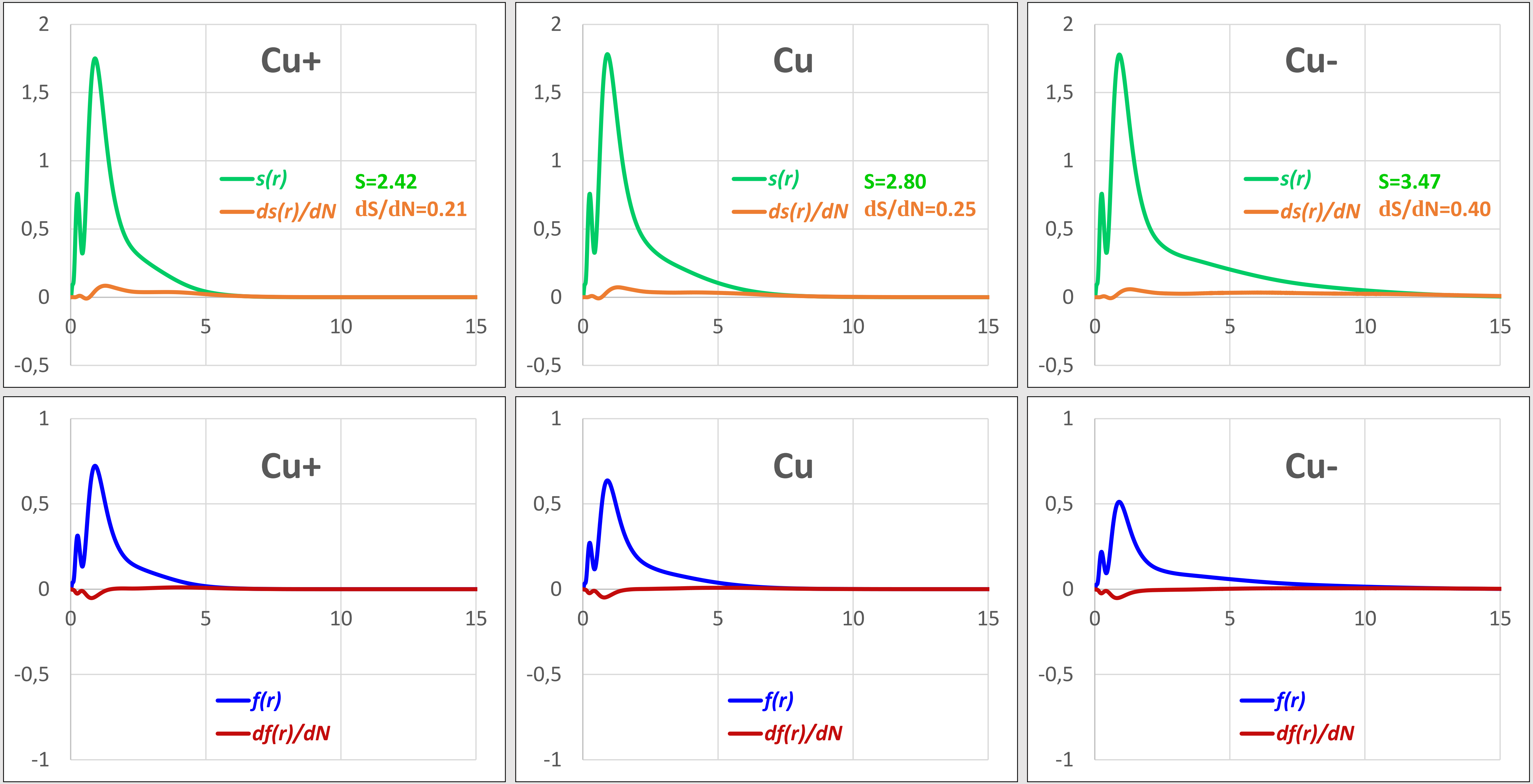
Cu+
s(r)
ds(r)/dN
S=2.42
dS/dN=0.21
Cu
s(r)
ds(r)/dN
S=2.80
dS/dN=0.25
Cu-
s(r)
ds(r)/dN
S=3.47
dS/dN=0.40
Cu+
f(r)
df(r)/dN
Cu
f(r)
df(r)/dN
Cu-
f(r)
df(r)/dN

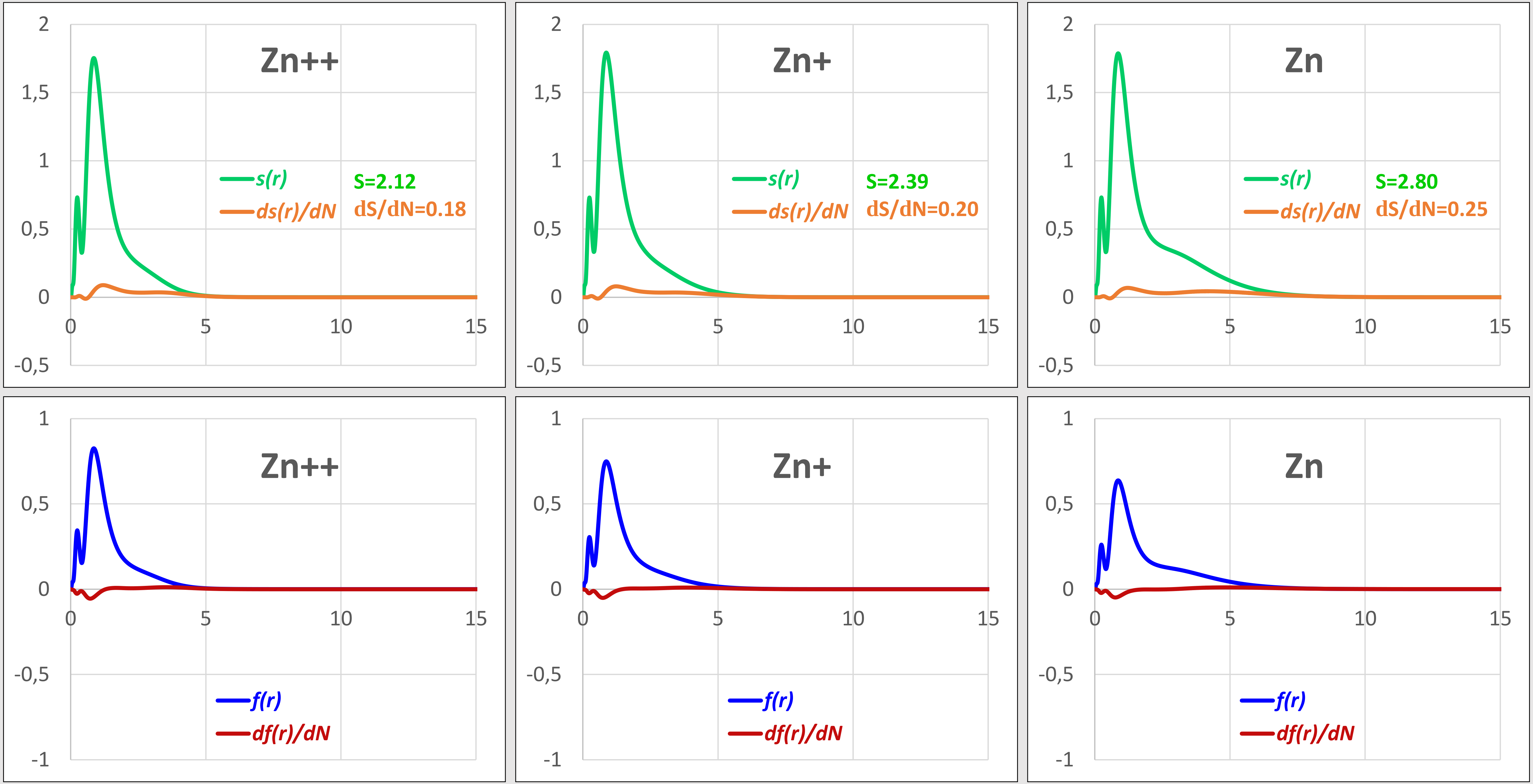

J. Zaklika, P. Ordon , L. Komorowski, Hyperhardness and hypersoftness of atoms and their ions,

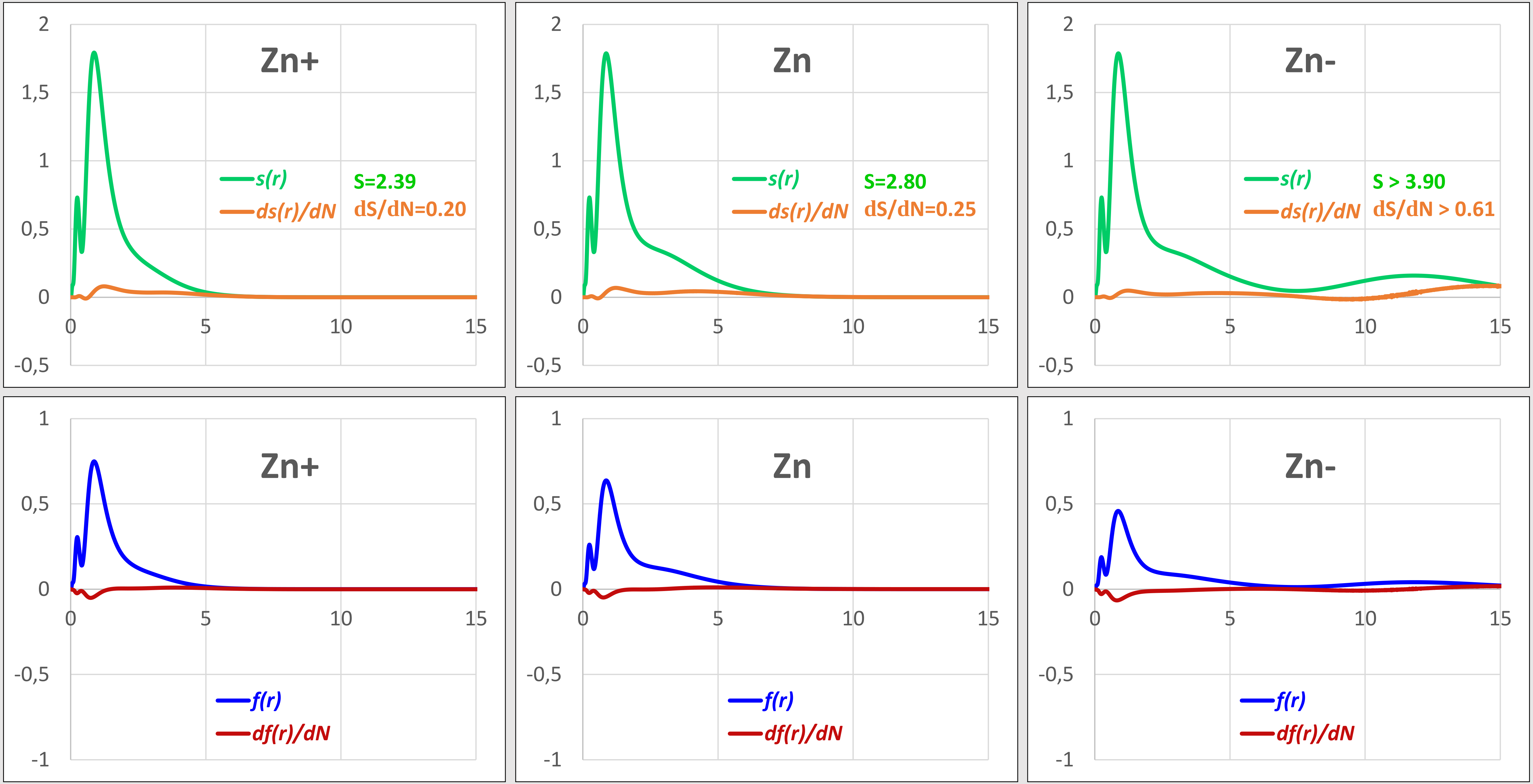

J. Zaklika, P. Ordon , L. Komorowski, Hyperhardness and hypersoftness of atoms and their ions,

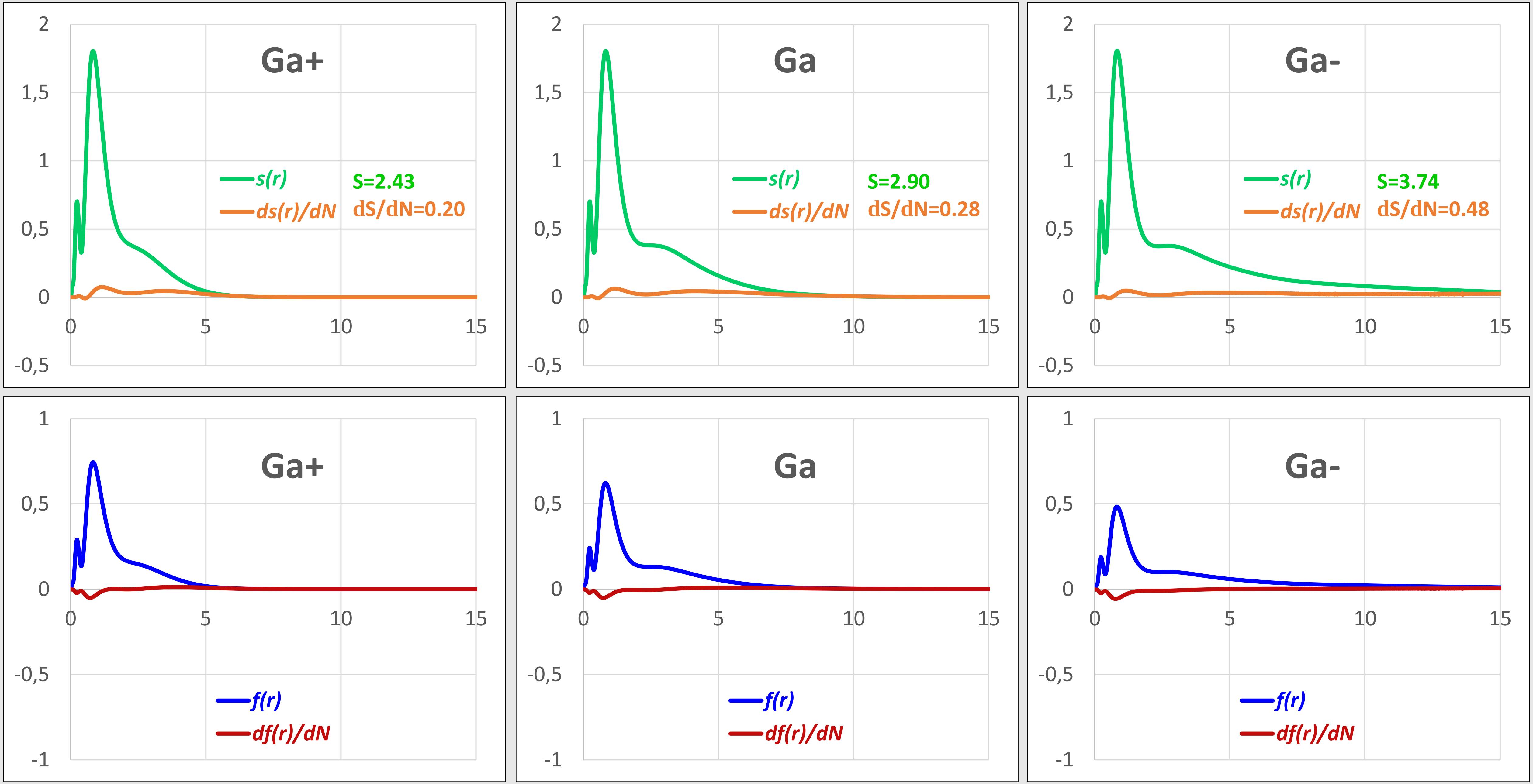
Ga+
Ga
Ga-
s(r)
ds(r)/dN
S=2.43
dS/dN=0.20
S=2.90
dS/dN=0.28
S=3.74
dS/dN=0.48
f(r)
df(r)/dN

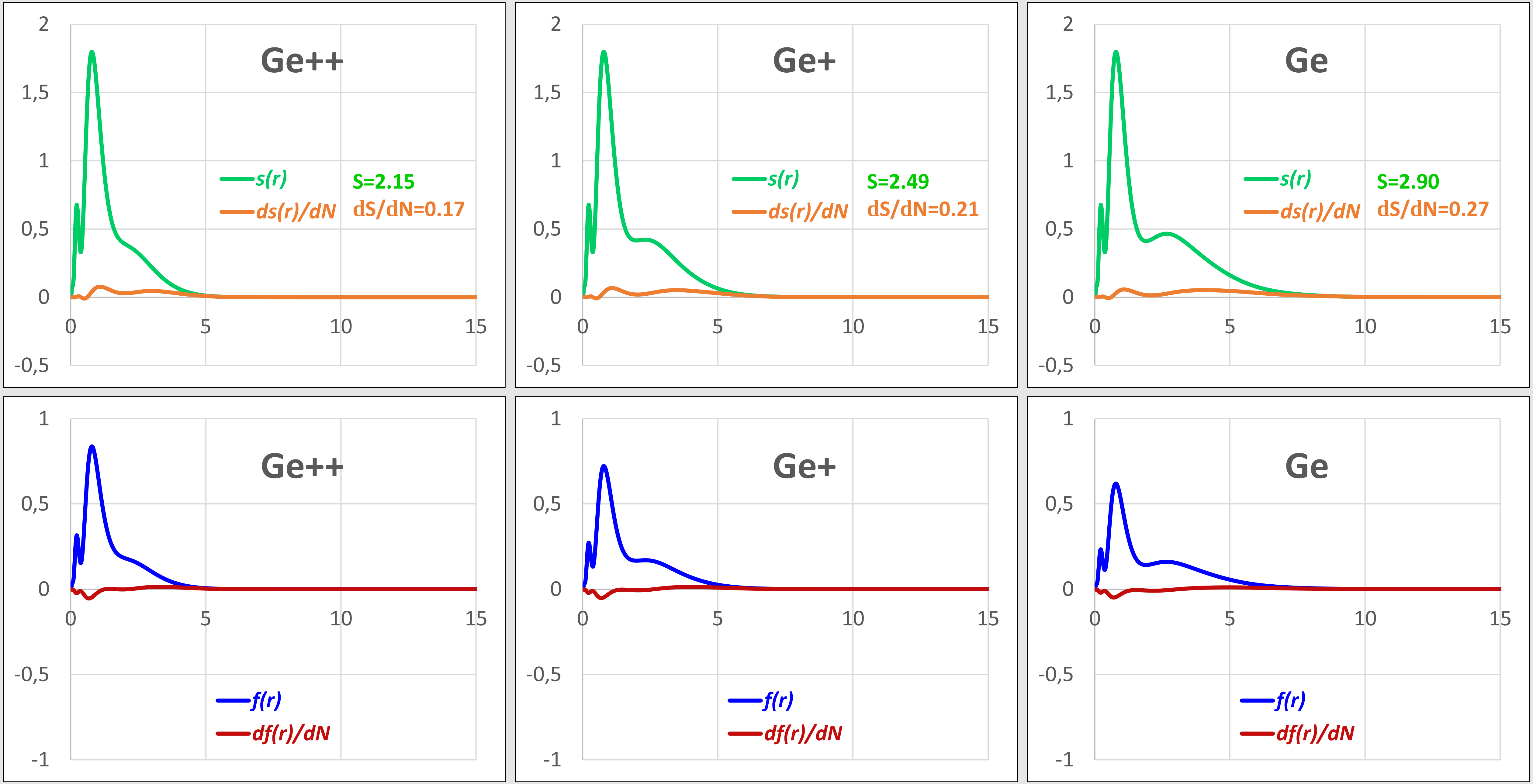

J. Zaklika, P. Ordon , L. Komorowski, Hyperhardness and hypersoftness of atoms and their ions,

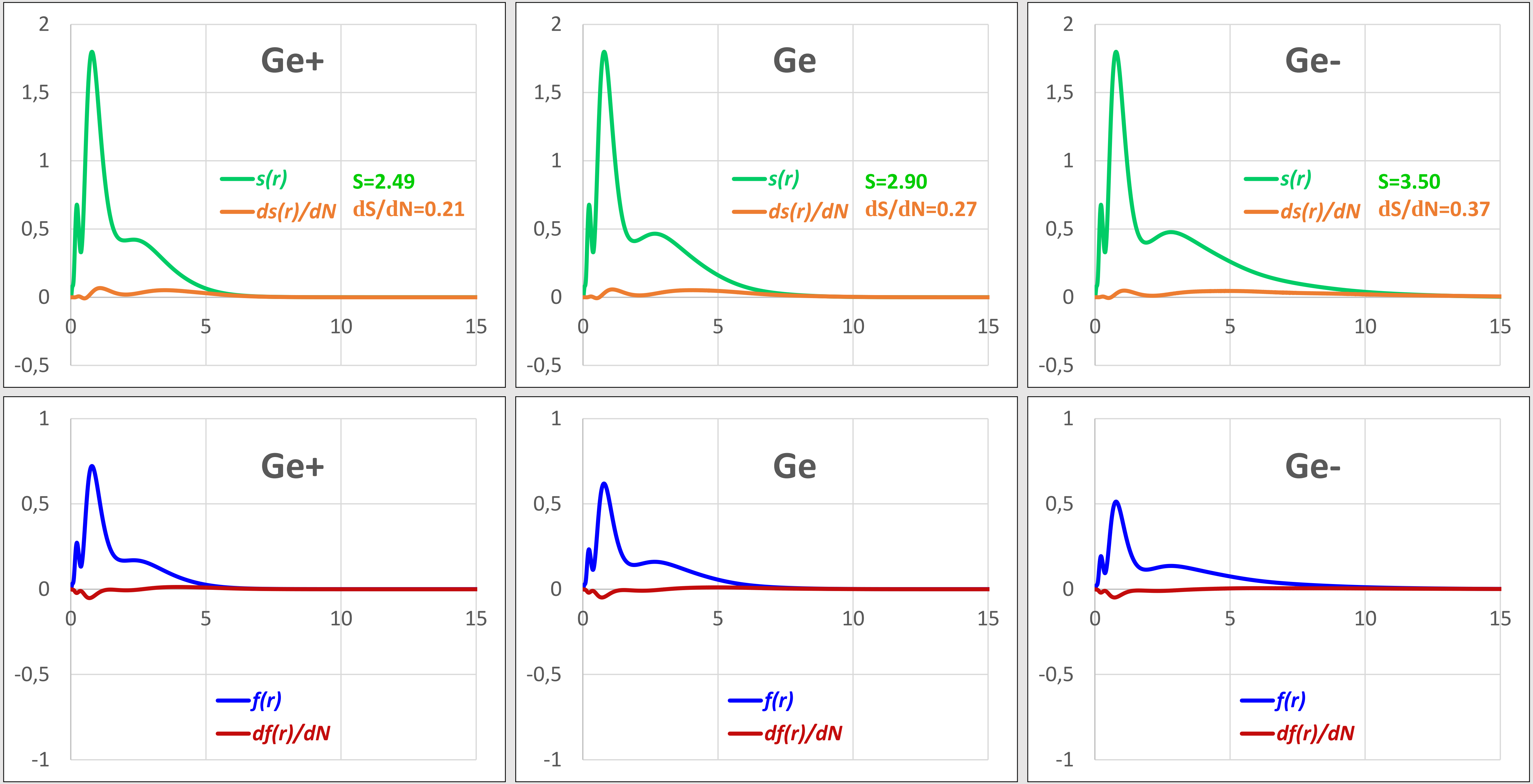

ELEMENTS

J. Zaklika, P. Ordon , L. Komorowski, Hyperhardness and hypersoftness of atoms and their ions,

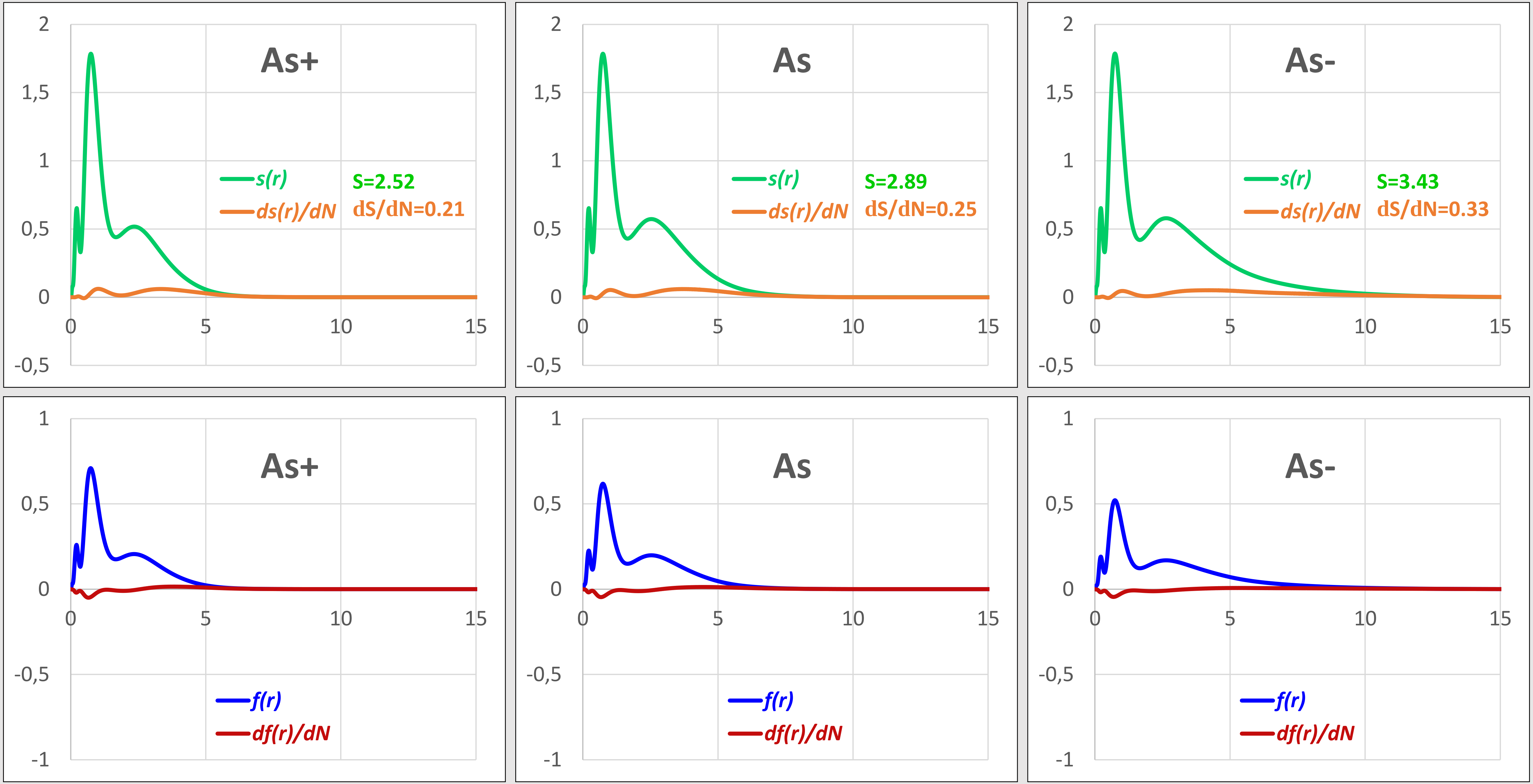

J. Zaklika, P. Ordon , L. Komorowski, Hyperhardness and hypersoftness of atoms and their ions,

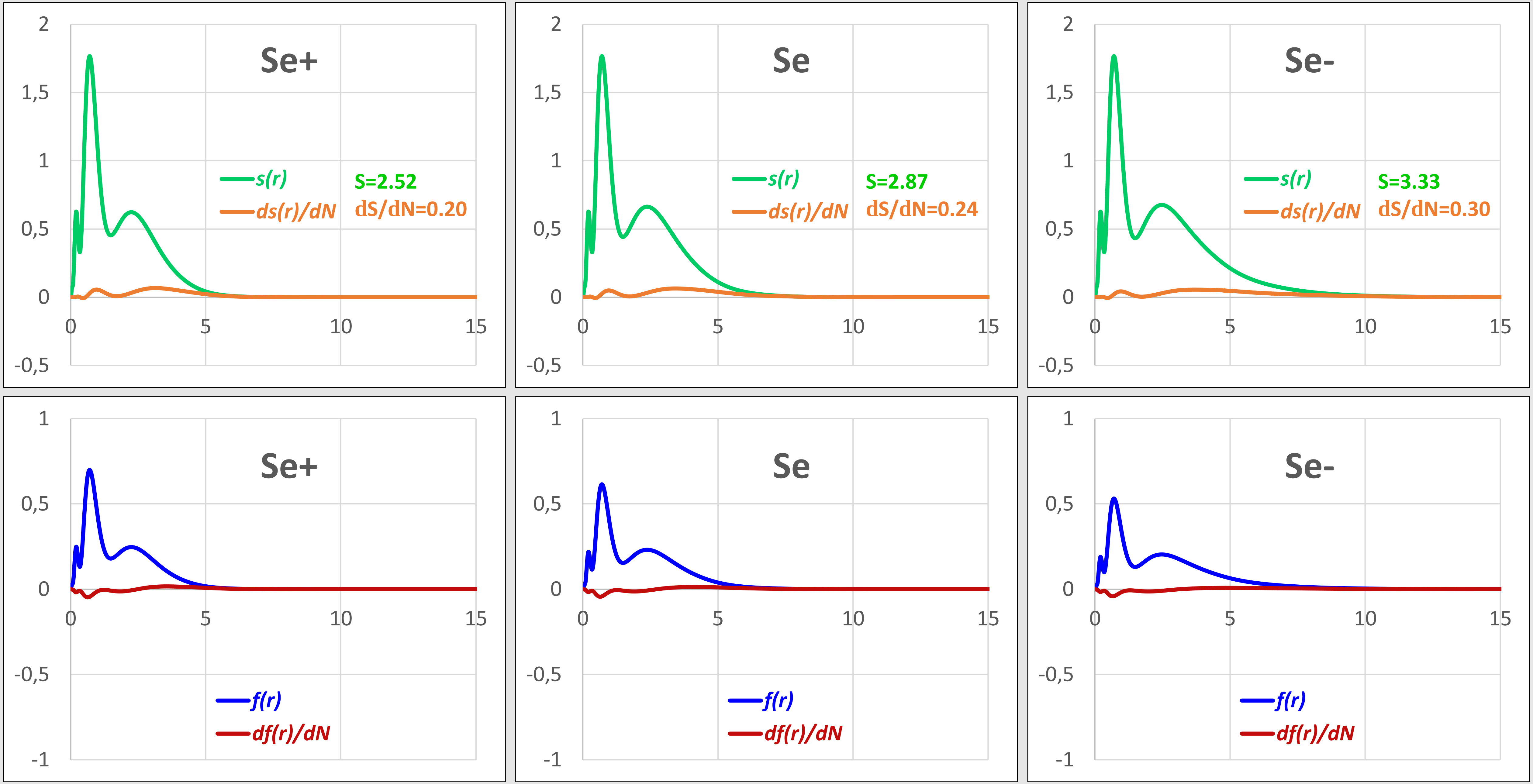

J. Zaklika, P. Ordon , L. Komorowski, Hyperhardness and hypersoftness of atoms and their ions,

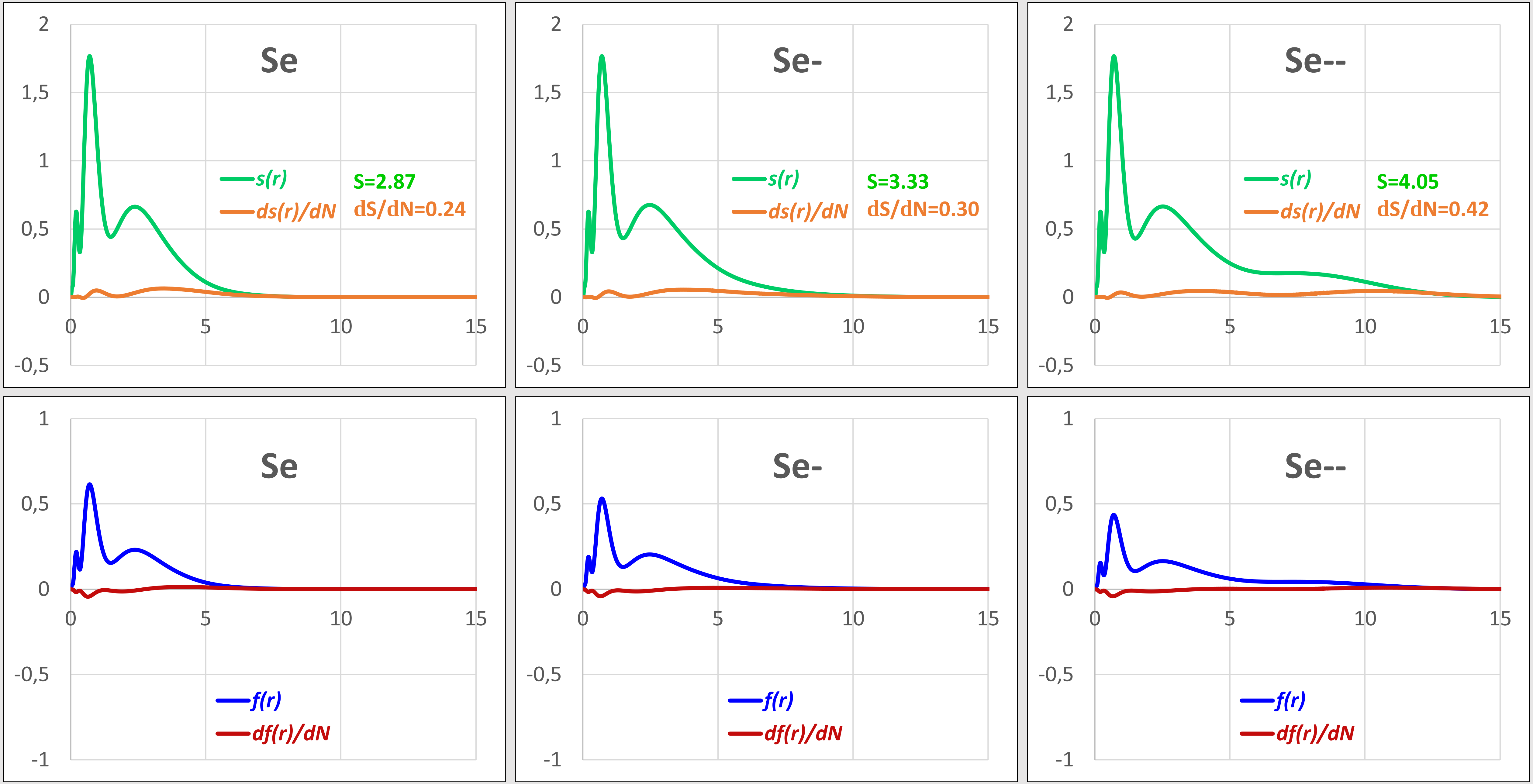

J. Zaklika, P. Ordon , L. Komorowski, Hyperhardness and hypersoftness of atoms and their ions,

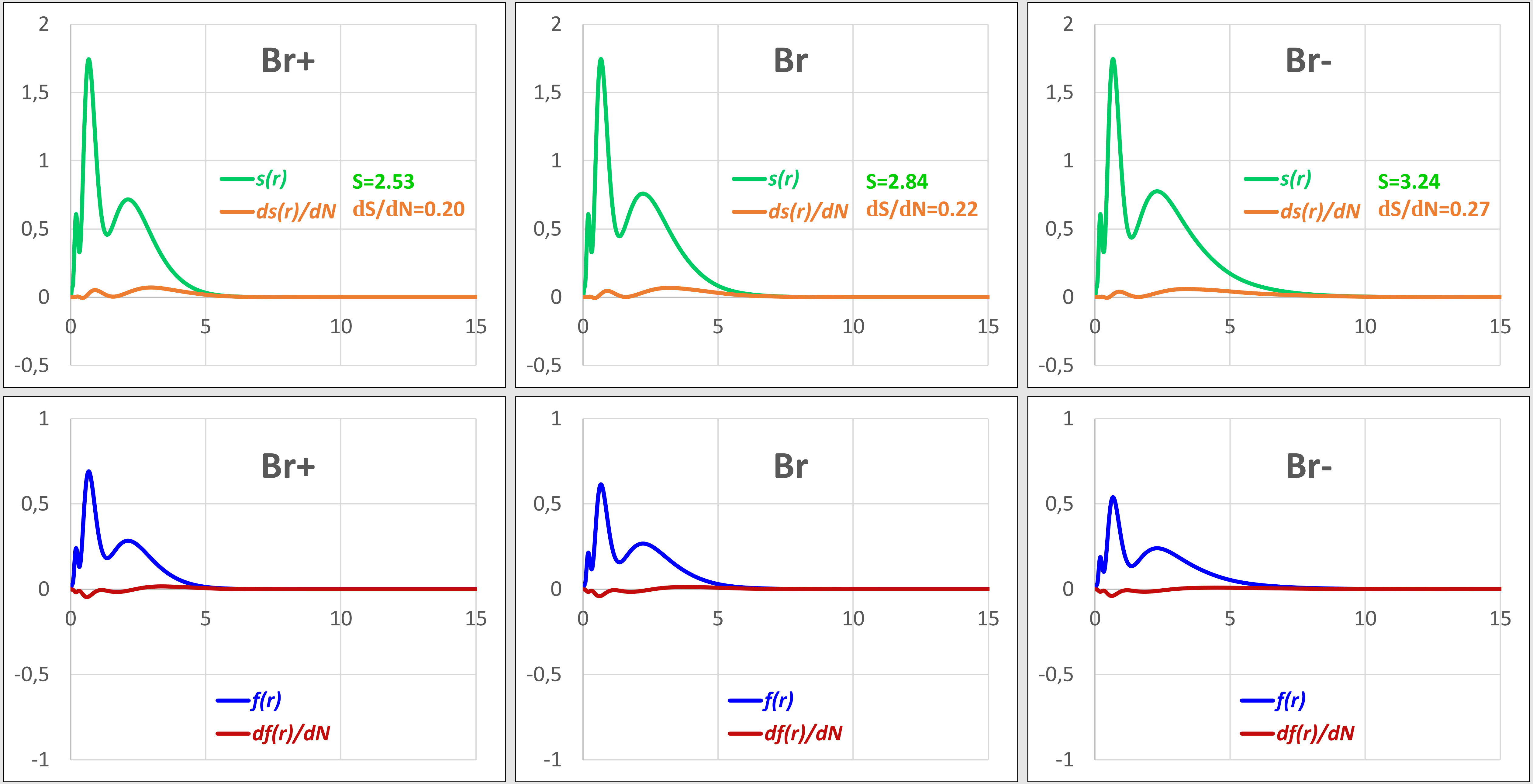

J. Zaklika, P. Ordon , L. Komorowski, Hyperhardness and hypersoftness of atoms and their ions,

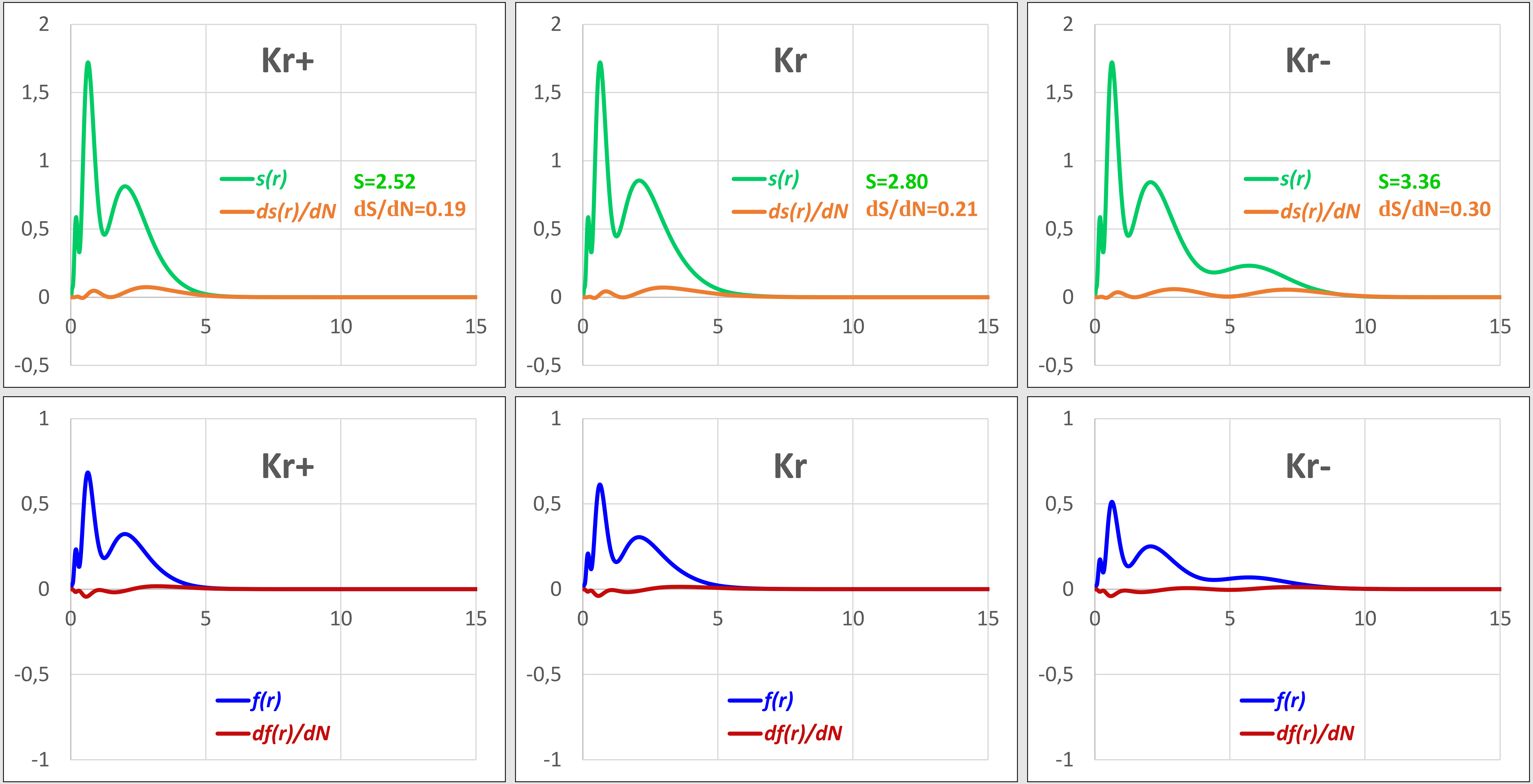

ELEMENTS

J. Zaklika, P. Ordon , L. Komorowski, Hyperhardness and hypersoftness of atoms and their ions,
